# Metabolic and Redox Signaling of the Nucleoredoxin-Like-1 Gene for the Treatment of Genetic Retinal Diseases

**DOI:** 10.3390/ijms21051625

**Published:** 2020-02-27

**Authors:** Emmanuelle Clérin, Myriam Marussig, José-Alain Sahel, Thierry Léveillard

**Affiliations:** 1 Department of Genetics, Sorbonne Université, INSERM, CNRS, Institut de la Vision, 17 rue Moreau, F-75012 Paris, France; emmanuelle.clerin@inserm.fr (E.C.); J.sahel@gmail.com (J.-A.S.); 2 SparingVision, 55 rue de Lyon, 75012 Paris, France; myriam.marussig@sparingvision.com; 3 CHNO des Quinze-Vingts, DHU Sight Restore, INSERM-DGOS CIC 1423, 28 rue de Charenton, F-75012 Paris, France

**Keywords:** retinitis pigmentosa, cone photoreceptor, central vision, rod-derived cone viability factor, aerobic glycolysis, thioredoxin signaling, gene therapy, adeno-associated viral vector, chemical manufacturing, clinical trial

## Abstract

The loss of cone photoreceptor function in retinitis pigmentosa (RP) severely impacts the central and daily vision and quality of life of patients affected by this disease. The loss of cones follows the degeneration of rods, in a manner independent of the causing mutations in numerous genes associated with RP. We have explored this phenomenon and proposed that the loss of rods triggers a reduction in the expression of rod-derived cone viability factor (RdCVF) encoded by the nucleoredoxin-like 1 (*NXNL1*) gene which interrupts the metabolic and redox signaling between rods and cones. After providing scientific evidence supporting this mechanism, we propose a way to restore this lost signaling and prevent the cone vision loss in animal models of RP. We also explain how we could restore this signaling to prevent cone vision loss in animal models of the disease and how we plan to apply this therapeutic strategy by the administration of both products of *NXNL1* encoding the trophic factor RdCVF and the thioredoxin enzyme RdCVFL using an adeno-associated viral vector. We describe in detail all the steps of this translational program, from the design of the drug, its production, biological validation, and analytical and preclinical qualification required for a future clinical trial that would, if successful, provide a treatment for this incurable disease.

## 1. Introduction

Inherited retinal degenerations (IRDs) are genetically and clinically heterogeneous blinding diseases that are now emerging as clinical targets for gene therapies [1,2,3]. The fervor of these ongoing efforts is motivated by the recent successful treatment of the *RPE65*-related form of Leber congenital amaurosis (LCA), a severe blinding disease, by a recombinant adeno-associated viral (AAV) vector that corrects the genetic deficit in those patients [4]. RPE65 is a success story, biblical in impact [5]. RPE65 was discovered in 1993 as a protein of 65 kDa expressed specifically by the retinal pigmented epithelium (RPE) [6]. The same group reported in 1997 that loss of function mutations in the gene encoding for RPE65 cause certain forms of LCA (*LCA2*) [7], and in 1998 that RPE65 is essential for the trans-isomerization of all-trans retinal esters, as they accumulate in the RPE of the *Rpe65-/-* mouse [8]. The booster of the story was the existence of a large animal model of *LCA2*, a briard dog discovered the same year [9]. A consortium of veterinarians, clinicians, and gene therapists reported the benefit of the subretinal injection of an AAV serotype 2 encoding for RPE65 in that dog model in 2001 [10]. Lancelot, one briard dog whose vision in the treated eye was restored, was photographed in front of the American congress during a meeting that restored the hope of future success of gene therapy after years of doubt [11,12,13]. This encouraged three independent research groups to initiate first in man studies that led to success during phases I of clinical trials, as reported on 2008 [14,15,16]. Nevertheless, the benefit of the treatment over long periods was controversial [17]. The group led by Jean Bennett made critical improvement of the treatment by showing the absence of advert effects associated with the treatment of the contralateral eye of the patients [18], while the two other groups reported the absence of benefit for the treated patients on the long-term [19,20,21]. The lack of persistence of the benefit of the treatment in some patients was interpreted in light of the natural history of the disease [22,23]. The absence of conversion of all-*trans*-retinol to 11-*cis*-retinal due to a mutation in the *RPE65* gene interrupts the resupply of functional, light-sensitive opsin proteins in photoreceptors [24]. The administration of a functional copy of *RPE65* by corrective gene therapy restores this deficit for vision. However, *LCA2* patients have retinal degeneration and loss of photoreceptors starting in the first decade of life [25]. The aggregation of short-wavelength cone opsins likely caused rapid cone degeneration through an endoplasmic reticulum stress pathway in an animal model with a genetic deficit of lecithin-retinol acyltransferase (LRAT), another LCA gene involved in the recycling of 11-*cis*-retinal in the RPE [26]. Consequently, if the disease has progressed to a certain stage, the restoration of the function of RPE65 is not able to stop photoreceptor degeneration. The results of a randomized phase 3 trial funded by Spark Therapeutics about the benefits of the subretinal injection of AAV2-hRPE65v2 developed by Jean Bennett were reported on July 2017. That study includes patients for which vision was preserved for three years [27]. The U.S. Food and Drug Administration (FDA) approved gene therapy using AAV2-hRPE65v2 (Luxturna^®^) for the treatment of patients with biallelic *RPE65* mutations in December 2017, followed by the European Medicines Agency (EMA) on November 2018 [4]. Spark Therapeutics announced that the company will follow the treated patients over 15 years.

Jean Bennet and her team were first of the rope [28]. Many other recessive inherited retinal degenerations could be treated by restoring the gene function altered by the mutations by introducing a normal copy of that gene, and this is what is happening [3]. The only negative argument against this approach is the very high cost for the society of having to correct more than one hundred genes one by one [29]. Issues of cost versus benefits will become increasingly important as a wave of costly new therapies tested in small patient populations are developed and approved (see [30] and Supplementary Table S1).

For dominant forms of IRDs, the situation is more complex like for the dominant mutations of the splicing factor gene *PRPF31* [31]. Surprisingly, most familial pedigrees contain affected and non-affected carrier individuals. This is explained by variable haploinsufficiency [32]. Those families are composed of members of different genetic compositions. For one subject, the dominant mutation in *PRPF31* on one chromosome is paired with a wild-type allele on the second chromosome with expression above a threshold. The residual protein level is sufficient for normal retinal function and this subject is asymptomatic. For another member of this pedigree, the dominant mutation in *PRPF31* on one chromosome is paired with a wild-type allele on the second chromosome with expression below this threshold. The residual protein level is not sufficient for normal retinal function and this subject is symptomatic. In this case the administration in the retina of a normal copy of the *PRPF31* gene should allow crossing this threshold of expression transforming patients into asymptomatic carriers.

The situation is even much more complex in the case of mutations in the rhodopsin gene, the first gene identified to cause retinitis pigmentosa (RP) [33]. Dominant mutations result in either gain-of-function or dominant negative functions [34]. They were stratified into six classes based on the molecular properties, with deficits of transport to the outer segment (class I), folding (class II), endocytosis (class III), protein stability (class IV), or alternatively increased activation rate for transducin (class V) and constitutive activation (class VI). Stimulating the unfolded protein response was shown to benefit rod function in a dominant rat model carrying a class II mutation [35]. The first therapeutic attempt in a rodent model of the disease used an engineered ribozyme, a catalytic RNA that specifically triggers the destruction of the mRNA encoding a class II P23H mutant rhodopsin mRNA [36]. Hammerhead ribozymes are able to induce site-specific cleavage of RNA, with ribozymes including two different oligoribonucleotides with regions of complementarity [37]. One obvious limitation of such approach is that the catalytic ribozyme is designed to target a specific mutation, while more than 150 point mutations in rhodopsin have been shown to cause RP [38]. Within the United States, the majority of rhodopsin mutations causing the disease is attributable to a single sequence variation at codon 23, suggesting a founder effect, but it is rare in Europe [39,40,41]. Diseases associated with mutations in this codon result in a relatively mild clinical phenotype [42,43]. To circumvent the large number of mutations, RNA interference-based mutation-independent approach was validated in various models RP with dominant mutations in the rhodopsin gene [44,45]. The strategy combines silencing the expression of the rhodopsin gene, both the mutant and normal rhodopsin genes, and simultaneously providing mRNA that encodes for the wild-type rhodopsin that is engendered to be resistant to the interfering RNA used by using the genetic code degeneration [46]. Botta et al. used the ability of the transcription factor Krüppel-like factor 15 (KLF15) to recognize a DNA element within the rhodopsin promoter to silence the expression of autosomal dominant mutant rhodopsin after delivery of an AAV vector–mediated ectopic expression of KLF15 in rod photoreceptors [47].

Recent progress in genome editing using clustered regularly interspaced short palindromic repeat/Cas9 (CRISPR/Cas9) technology led to the development of therapeutic approaches of RP by in vivo genome editing [48,49]. Targeting in vivo the neural retina-specific leucine zipper (*NRL*) gene in rods using CRISPR-Cas9 technology was also used to repress rod differentiation to circumvent the deleterious effect of mutations of genes expressed specifically by rods [50]. Other mutation-independent approaches were studied aiming at preventing an event common to all forms of rod cell death, starting by targeting apoptosis since it is the final fate for rods in animal models and for patients suffering of RP [51,52]. Overexpression of histone deacetylase 4 (HDAC4) or even its N-terminal domain suppresses multiple cell death and prolongs rod survival in an autosomal recessive model of RP, the *rd1* mice [53]. Administration of ciliary neurotrophic factor (CNTF) protects photoreceptors of murine models of RP [54], but was not confirmed in the first clinical trial [55]. Downregulation of microglia activation using either the microRNA miR-204 [56] or fractalkine, a soluble form of the chemokine CX3CL1 prevent and photoreceptor cell death in several mouse models of RP [57]. Oxidative damage was successfully targeted by delivering the transcription factor nuclear factor, erythroid 2 like 2 (NFE2L2), known as NRF2, that regulates hundreds of genes that preserve photoreceptors against oxidative stress [58]. Iron may be involved in oxidative damage since intraperitoneally-injected transferrin prevents photoreceptor degeneration in *rd10* mice [59]. Other approaches have been used to sustain photoreceptor metabolic needs [60,61].

One main limitation of these approaches targeting rods is that patients suffering of RP are often identified by ophthalmologists after consultation at a stage where rod photoreceptors are already dead. Rod degeneration leads to night blindness so, for patients living in an urban setting, night vision problems may not be apparent until the retinal disease is in an advanced stage. Consequently, preventing the degeneration of rods will not be beneficial to these patients.

## 2. Rod-Derived Cone Viability Factor

The diagnosis of RP has probably existed historically for hundreds, if not thousands of years, but was recognized as a complicated form of night blindness only after the invention of the ophthalmoscope in 1851. In RP, there is a progression from night blindness which originates from the death of rod photoreceptors by apoptosis, to the dysfunction of cone photoreceptors concentrated at the center of the retina, the fovea. This secondary event leads to complete blindness [62,63]. On the other hand, congenital stationary night blindness (CSNB) does not lead to the loss of central vision. The most remarkable CSNB pedigree comprising 2121 people goes back to Jean Nougaret, a butcher of the 17th century (1637–1719) of Vendémian in the Hérault. In 1831, a fifth-generation descendant demanded an exemption from military service because of his poor night vision. Unfortunately for him, the examination found that the young man could read by candlelight, so in photopic conditions that involve the function of cones, and the doctor concluded by a simulation and found it good for seven years of service. It was only in 1838 that the family transmission of his night blindness was demonstrated by Belgian ophthalmologist Florent Cunier, freeing him from military duty [64]. In 1996, a missense mutation in the gene encoding the rod transducin alpha subunit (*GNAT1*) responsible for the pathology of the Nougaret family was identified in eleventh-generation members [65]. This entertaining story has its importance today since some persons within this large pedigree, uncertain of being affected, were shown to be as night-blind as their relatives through the same *GNAT1* mutation [66]. The night vision disturbance of CSNB is overlooked because it is highly subjective especially for individuals living in an urban or well-lit environment [67]. This led Professor Alan Wright to declare that keeping the cones alive will help some 1.5 million people worldwide to see, as well as Jean Nougaret and his descendants [68]. More recently Professor Henry Kaplan restated that if therapy can prevent or reverse the onset of cone degeneration within the macula most patients would be immeasurably helped and able to live normal lives [69]. This strategy is medically applicable since, in patients and in a dog model of RP, visual discriminance can be achieved with a very limited number of functional cones [70,71].

What causes the dysfunction and ultimately the death of cones remained obscure for years [72]. The identification of the first mutation causing RP in human left the scientific community perplexed. After having localized the gene on a short interval of the long arm of chromosome 3, a dominant mutation in the gene encoding for the rhodopsin, the visual pigment of rods, was identified [73,74,75]. Electroretinogram (ERG) measurements of affected members of this pedigree confirmed that RP progresses from an early abnormal rod response to a subsequent altered cone response. In advanced cases, RP is associated with a complete loss of rods, with a few remaining foveal cones with shortened and disorganized outer segments [76]. The reason why a mutation in a gene expressed exclusively in rods can lead to widespread degeneration of both rods and cones in these patients was puzzling [77]. It was observed that cone outer segment function remains normal until >75% of the rod outer segment are lost [78].

This two-phase degeneration sequence was initially described in retinal degeneration (*rd1*) mice by Carter-Dawson et al. [79]. The *rd1* mouse carries a recessive mutation of the cGMP-dependent phosphodiesterase beta subunit gene, *Pde6b*, selectively expressed by rods [80]. Mutations in the human orthologue gene, *PDE6B*, are causing RP [81]. Specific ablation of rod photoreceptors in transgenic mice results in the failure of cone cells in elaborating their outer segments [82,83]. It was observed in various animal models of RP including rodents, pigs, dogs, and even toads [83,84,85,86]. The analysis of the kinetics of rod and cone cells death in several mouse models of RP excludes that dying rods are toxically damaging the cones [87]. Transplantation of a wild-type photoreceptor layer (97% rod photoreceptors in the mouse) in the subretinal space of the *rd1* mouse slows down the secondary degeneration of cones [88,89]. We showed that the effect is mediated by soluble molecules originating from the neural retina and not from the adjacent RPE [90]. Partial characterization of this trophic interaction demonstrated that the activity is carried by molecules having the apparent molecular weight compatible with that of proteins [91]. In the same study, we showed that the fraction is active on cone-enriched culture made from chicken embryo, a model originally developed by Ruben Adler [92]. The existence of cone-protecting proteins in the extracellular matrix surrounding the photoreceptors has been suggested using this biological system, where the photoreceptors spontaneously degenerate between seven and 10 days in culture [93]. The degeneration of rods in the *rd1* retina could result in the loss of expression of secreted protein factors necessary for cone survival. In RP patients, the secondary cone dysfunction leads to blindness since the cones are responsible not only for the vision of the colors, but also for all useful functions (perception of details, reading) of daylight vision [94].

The identification of proteins able to prevent the spontaneously cell degeneration in the cone-enriched culture was performed by high content screening [95]. The screened factors were produced by transient transfection of a retinal cDNA library in COS-1 cells. The conditioned media of the equivalent of 210,000 clones containing, among others, the secreted proteins were applied to primary cones from chicken and the viability was scored after seven days. A cDNA with an open reading frame of 109 amino acids was identified and the protein was named rod-derived cone viability factor (RdCVF). RdCVF is a splicing variant of the nucleoredoxin-like-1 (*NXNL1*) gene corresponding to intron retention and a conserved in-frame stop codon (OMIM # 608791) translated from a ~7 kb messenger RNA [96]. *NXNL1* expression is rod-dependent in the mouse and human retina and its promoter is regulated by the homeoprotein CRX [97,98,99]. The injection of recombinant RdCVF protein protects the cones of the *rd1* mouse from degenerating [95]. In a transgenic model of autosomal dominant RP with expression of the mutant rhodopsin protein under the control of a rod promoter, we showed that a synthetic human RdCVF protein prevents secondary cone degeneration that could be scored using photopic ERG which scores cone function [100]. The protection, observed over a period of three months, was associated with the preservation of the outer segments of the cones, the subcellular structure that carries the light sensitive molecules, the opsins [101,102]. Similar results were obtained by the administration of RdCVF through gene therapy [102]. More recently, we showed that cone function can be preserved in the *rd10* mouse, a second model of recessive RP, by delivering RdCVF using a recombinant AAV vector [103]. The effect of RdCVF is independent of the genetic origin of the disease, so a therapy aimed at preventing central vision loss should benefit to all RP patients whatever the mutation they carry in any of the 65 genes known to date [104].

A cell surface receptor for RdCVF was identified by far-Western blotting [96]. The single-pass transmembrane domain protein basigin (BSG) is expressed in two different isoforms. The protein BSG2 possesses two extracellular immunoglobulin (Ig) domains and is expressed widely in many organs where it is involved in addressing lactate transporters to the membrane. The protein BSG1, produced by alternative splicing, possesses a third Ig domain, and its expression is restricted to photoreceptors [104]. We found that RdCVF interacts specifically with a complex made of BSG1 and the glucose transporter GLUT1 (SLC2A1) at the surface of the cones. The increased glucose entry into the cones is metabolized by aerobic glycolysis [105]. The bifurcation from oxidative phosphorylation takes place after the production of pyruvate by glycolysis. The pyruvate (PYR) can be transported to the mitochondria by the mitochondrial pyruvate carrier (MPC) where it is metabolized by the tricarboxylic acid cycle (TCA) that is connected with the mitochondrial respiratory chain to produced ATP in aerobic conditions. The whole process is known as oxidative phosphorylation (OXPHO). The pyruvate can alternatively be metabolized to produce lactate (LACT) by lactate dehydrogenase (LDH). Lactate is then transported out of the cell by lactate transporters (MCTs). RdCVF stimulates aerobic glycolysis to provide triglycerides that are used by cones for the renewal of the outer segments. For reasons that are only partially known, aerobic glycolysis favors the diversification at mid-course of the glycolytic reaction of carbons from glucose at the level of dihydroxyacetone phosphate (DHAP). A certain proportion of DHAP is converted into glycerol-3-phosphate (G3P) by glycerol-3-phosphate dehydrogenase (G3PDH). G3P is the precursor of the hydrophilic head of phospholipids made with fatty acids, among which poly-unsaturated fatty acids (PUFA) are derived from nutrition. Recently, it was demonstrated experimentally that, for rods, aerobic glycolysis is necessary for rod outer segment renewal, a phenomenon parallel to that described for cones [106].

## 3. The Thioredoxin RdCVFL

Interestingly, the splicing of the unique intron of the *NXNL1* gene produces a second messenger RNA encoding for an enzymatically active thioredoxin, RdCVFL [107,108]. Both rods and cones of the mouse carrying a homologous recombination of the *Nxnl1* gene are affected, a deficit that progresses with age [109]. The lack of expression of the thioredoxin RdCVFL leads to increased oxidative damage. We observed a higher concentration of molecular adducts produced by lipid peroxidation in the retina of these mice. The phototransduction is initiated in the outer segment of photoreceptors made of stacks of lipid bilayers where the opsin molecule is enchased. These membranes are particularly enriched in PUFA which are prone to oxidation [110]. The retina of a mouse with a specific deletion of the *Nxnl1* in cones is more susceptible to oxidative damages since *Nxnl1* is also expressed by cones (3% of all photoreceptors in the mouse) [111]. Contrarily to the rods, there is no intron retention of the *Nxnl1* mRNA in the cones that express only the thioredoxin RdCVFL.

Reactive oxygen species (ROS) are produced in physiological conditions by leakage from the mitochondrial respiratory chain and inhibits key enzymes in the glycolytic pathway [112,113]. The thioredoxin system reduces oxidized thiol groups of cysteines in proteins among which glycolytic enzymes and restores their function [114]. The thioredoxin enzymes, among which RdCVFL must be reduced by thioredoxin reductase (TXNRD) since the catalytic cysteines become oxidized when the enzyme reduces its substrate [115]. TXNRD requires its co-factor NADPH produced by the pentose phosphate pathway (PPP) in its reduced form to function. Consequently, the reducing power of RdCVFL in cones is proportional to the uptake of glucose and the rederivation of the glycolytic flux to the PPP. ROS inhibition of glycolysis leads to the accumulation of glucose-6-phosphate (G6P), the first metabolite produced from glucose in the cell, which is redirected to the PPP producing two molecules of NADPH by reduction for TXNRD activity [116,117]. In this metabolic and redox signaling, the reducing power of RdCVFL relies on the metabolism of glucose by cones whose uptake is stimulated by RdCVF [118].

The combination of the non-cell autonomous action of RdCVF on metabolism with the cell-autonomous activity of RdCVFL on redox homeostasis is the rational of a current translational program aimed at preventing the loss of central vision, resulting from cone dysfunction, in RP patients independently of the causative mutations.

## 4. Design of the Therapeutic Molecule

**Figure 1:**
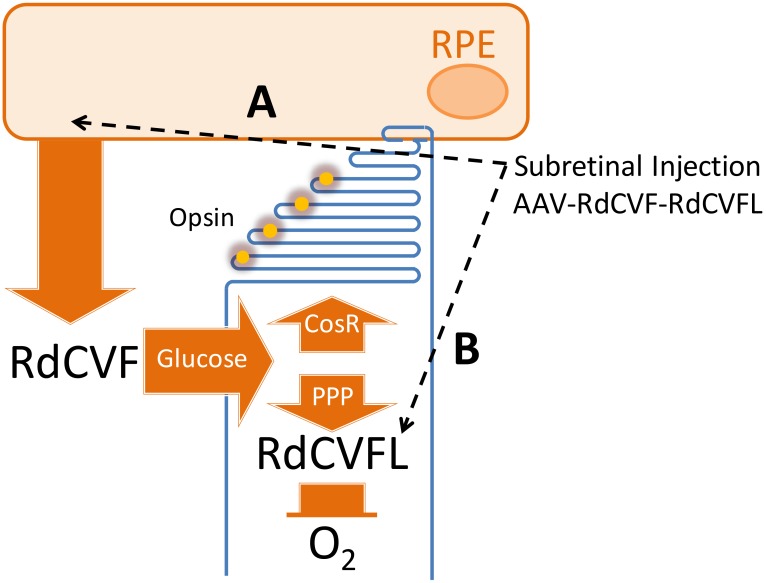
Subretinal injection of AAV-RdCVF-RdCVFL to prevent the loss of function of cones in retinitis pigmentosa, (**A**) Transduction of the drug (recombinant AAV) to the retinal pigmented epithelium, expression and secretion of RdCVF to stimulate glucose entry, cone outer segment renewal (CosR) and the pentose phosphate pathway (PPP). (**B**) Transduction of the drug (recombinant AAV) to the cones and stimulation of redox homeostasis through NADPH, reduced by the metabolism of glucose through the pentose phosphate pathway.

The actions of both RdCVF and RdCVFL seem to be required for the maintenance of cone function throughout life, so that repeated injections of the recombinant proteins are precluded. As in the case of Luxturna^®^, the development of an AAV vector encoding for RdCVF and RdCVFL would provide a long-term protection of central vision in patients. These two therapeutic products are made by alternative splicing of the *NXNL1* gene in the rods, but our current knowledge of the regulation of splicing of this gene makes the use of a genomic construct hazardous. The combination of two distinct human cDNAs encoding for RdCVF (109 amino acids) and RdCVFL (212 amino acids) would be a rational approach. The configurations of two cassettes on opposite strands, with 5′ or 3′ ends pointing the external invert terminal repeats (ITRs), could result in the formation of palindromes that could interfere with encapsidation of the single strand DNA into the viral particle and are thought to be at risk for the development of the drug [119]. In a safer configuration, the two cDNA are arranged tandemly on the same strand. The reduction of tandem repeats sequence of the common part of the two cDNAs is obtained by modifying the used codons in order to make the vector more stable during its production [120]. Notice that this is a deviation of codon optimization since this method applies a codon optimization algorithm to a human cDNA sequence for making the translation of the protein more efficient in human cells [121]. The expression of these cDNAs is regulated by a promoter. Since RdCVF is acting in a non-cell autonomous manner, the cytomegalovirus (CMV) promoter fused to the chicken β-actin (CBA) promoter that drives the expression of the transgene in most cells is particularly suited [122,123] (Figure 1A). Such ubiquitous promoter has been used successfully in experiments aimed at protecting the cones in vivo [96,103]. The protective action of the thioredoxin RdCVFL is cell autonomous, so that its cDNA should be expressed by the cells that are protected, the photoreceptors, rods, and cones [111,124]. Since our strategy is to restore the metabolic and redox signaling in the retina of patients having lost their rods, a cone specific promoter appears as the most effective solution. In previous work, we have successfully used a hybrid promoter made of the locus control region (LCR) of the cone’s red (L) and green (M) promoter fused to the proximal promoter region of the red opsin gene (OPN1L/MW) to deliver RdCVFL to the cones [125,126] (Figure 1B). In order to reduce the contribution of the promoter located 5′ in the construct to the expression of the transgene located in the 3′ position, it is advisable to introduce a transcription terminator element that will isolate both cassettes [127,128]. It is important to maintain the entire size of the construct including the external ITRs in the limit of what can be encapsidated efficiently in the AAV particle of 25 nm [129]. AAV can package a vector larger than its genome size, up to 5.2 kb, the packaging of larger or smaller loads is suboptimal and the transduction of cells less efficient [130,131,132]. AAV vector genomes never exceeding 5.2 kb in length can accommodate the RdCVF-RdCVFL expression cassettes. The two cassettes are introduced in a proviral plasmid whose size is largely superior to 5.2 kb in order to avoid reverse packaging of vector sequences during the production of the viral particles by the use of a long stuffer sequence [133]. In order to assure that this stuffer sequence is biologically inert, researchers in the field have used a fragment of the genome of bacteriophage lambda, while this sequence contains several short open reading frames [134]. These elements combined with possible reverse packaging may be viewed as a risk by regulatory authorities. One way to circumvent this possible problem is to identify inert sequences in the human genome; sequences that do not contain any signal or structural particularities. The possible encapsidation of prokaryotic sequences during recombinant adeno-associated virus production questions the resistance gene used to propagate the proviral plasmid [135]. To date, the use of the β-lactam antibiotic family, including ampicillin, is not acceptable for regulatory authorities, in order to avoid concerns for patients showing allergic reactivity to β-lactam antibiotics. Therefore, the kanamycin gene is commonly used. The proviral plasmid should be entirely sequenced on both strands after its assembly. Special care is devoted to the sequence of the ITRs because of their role in the production of active AAV particles, their instability, and the difficulties to access to the sequence due to the presence of DNA secondary structure [136]. The construct should be then validated by analyzing the expression of the two transgenes after transient transfection and viral infection of cells in vitro. This presents no difficulty for RdCVF that is expressed under the control of a ubiquitous promoter, but requires more effort for RdCVFL that is regulated by a cone-specific promoter [137].

## 5. Chemical Manufacturing

As a prerequisite to the production of AAV particle to be used in a clinical trial, all plasmids must be produced under conditions consistent with current good manufacturing practice (GMP) [138]. They have to be free of transmissible spongiform encephalopathy and bovine spongiform encephalopathy. They must have a very low level of endotoxin, of residual host cell protein, and be of genomic DNA. Recombinant AAV particles are most commonly produced by co-transfection of three distinct plasmids in mammalian cells vectors: (1) the proviral plasmids containing RdCVF and RdCVFL and their regulatory sequences; (2) the AAV rep and *cap* genes provided in trans; and (3) helper functions from adenovirus for efficient replication of the recombinant genome [139]. The serotype of the AAV particles can be modulated by the use of a plasmid encoding the capsid proteins VP1, 2, and 3. While the transgene is more commonly constructed with ITR sequences from AAV serotype 2, the capsid proteins provided in trans can specify a distinct serotype of the particles, for example, serotype 5 [140,141]. Since RdCVF is acting in a non-cell autonomous manner, its administration does not need to target specific cells in the retina and it can even be administrated in the animal in systemic ways [96,103]. Thioredoxin RdCVFL acts in a cell-autonomous manner of photoreceptors, so the potent serotypes are those reported to transduce cones in the case of RP, as serotypes 5, 8, and 9 [111,142,143]. As an alternative to mammalian cells, a protocol using invertebrate cell lines from *Spodoptera frugiperda* (Sf9) infected with the baculovirus *Autographa californica* was also developed [144,145]. Since Sf9-produced AAV have altered capsid compositions and lower biological potencies, we will not discuss their use further [146]. The most established method for the production of AAV vectors is plasmid tri-transfection of human embryonic kidney (HEK)293 cells [147]. The HEK293 cell line has been generated by transfection of cultures of normal human embryonic kidney cells with sheared adenovirus 5 DNA containing the adenovirus E1A and E1B genes [148]. HEK293 cells express the oncogenic protein E1A that binds to the retinoblastoma protein encoding by the tumor suppressor gene *RB1* [149]. Consequently, the clinical batches of recombinant AAV must be checked for the absence of E1A DNA [150]. A subclone of HEK293 cells has been generated by transfection with a plasmid encoding a temperature-sensitive mutant of the simian virus (SV)40 [151]. SV40 large T antigen (SV40T) binds to the tumor suppressor p53 protein, and this association contributes to oncogenic transformation by the virus [152]. Thus, even if the productivity of AAV particles is higher in HEK293T than in HEK293 cells, it is advisable to use HEK293 to avoid additional potent problems with the presence of trace of SV40T DNA [153]. HEK293 are usually grown as adherent cells in the presence of fetal bovine serum that must be purchased from countries exempted of spongiform encephalopathy [154]. Since the productivity of AAV particles depends not only on the cells used but also of the sequence of the transgene, it is necessary to develop the manufacturing process by varying the conditions (plasmid quantities and ratio, duration of transfection, time of harvest, etc.) to define the conditions of what is called the upstream process to optimize the yield.

The downstream process is the purification of the AAV particles after their production by HEK293 or HEK293T cells. Isopycnic ultracentrifugation on cesium chloride (CsCl) gradient allows the separation of the particles with single-strand DNA packaged in it (full particles) from those without DNA (empty particles) due to the difference in their buoyant density [155]. Alternatively, the particles are purified using iodixanol-based density gradients with a ratio full/empty inferior to the CsCl gradient method. Since performing ultracentrifugation is a rupture in the chain of downstream processes it is complicated for producing clinical grade AAV. This is the reason for the development of alternative approaches using chromatography, a process that is performed in a pipeline from upstream to downstream process [156]. Affinity chromatography cannot distinguish between the full and empty capsids, but empirical assays led to the development of chromatographic methods to enrich for full particles [157]. The proportion of full, empty, and partially encapsidated particles is analyzed by analytical ultracentrifugation [158]. Another important aspect is the measurement of the titer of the preparation that is most commonly achieved by quantitative PCR (qPCR). Accurate titration is critical for ensuring correct and reproducible dosing in both preclinical and clinical settings. Attempts to standardize the protocol over platforms have shown little success [159]. A potential problem with qPCR is its dependence on a DNA standard curve that itself can be incorrectly calibrated. Currently, the method of choice is an absolute quantification using droplet digital PCR [160,161]. It remains that the titer of the AAV preparation that is biologically active in the proof-of-concept experiments in a research laboratory has to match that of the clinical-grade production, a task that requires the use of an AAV biological standard. This is complicated when the protocol of purification of AAV differs between the research laboratory and the clinical platform, for example, when the proof-of-concept of the therapeutic benefit has been obtained with research-grade AAV purified by ultracentrifugation on CsCl or an iodixanol gradient to clinical-grade AAV purified by methods more adapted to industrial production [147,162]. It is not only that the latter contains empty particles but potentially a certain proportion of partially encapsidated particles that can be scored as positive in the titration using droplet digital PCR but do not transduce the entire transgene. The quantity of full versus partially encapsidated particles can be assessed using alkaline gel electrophoresis [163].

When the full manufacturing process is fully optimized and the analytical tests for the limited quantities of host cell proteins, host cells, and plasmid DNA, and the absence of production of replicative particles are developed, a first production of the therapeutic AAV is made as a reference for any further comparison. Then, a second production following exactly the same methods provides the pilot material for the proof-of-concept of its activity in the animal model(s) of the disease.

## 6. Preclinical Studies

An important step in the transfer of the treatment of RP using the combination of RdCVF and RdCVFL is to provide a proof-of-concept in an animal mouse model of the disease using the pilot material of the clinical-grade AAV. We can use a mouse model of recessive RP, and the *rd10* mouse is considered to be the most appropriate to test the benefit on the combination of both products of the *NXNL1* gene [103,111]. This model is the most appropriate to test for the benefit of the combination of both products of the *NXNL1* gene. Interestingly, this model can be used to measure cone function using optometry, a behavior assay that relies on head tracking movement that is proportional to acuity and contrast sensitivity [164]. The effect of the treatment of *rd10* is evaluated by several temporal sequences of electroretinogram (ERG) measurements of cone function by photopic and flicker ERG and optometry assays [103,111,137]. At the end of each experiment, the density of the cones is measured using e-conome after the sacrifice of the animals [165]. In this model, those three measures are proportional and provide robustness to the results. The experiment setup is crucial at this stage of the project for several reasons. First, the kinetics of degeneration of photoreceptors in the *rd10* mouse is influenced by light [166]. To avoid any artifactual differences between the individually-treated animals, all the mice should be exposed to the same quantity of light over the entire duration of the experiment. This is achieved through horizontal illumination from light sources fixed on the wall, the only solution to ensure that homogeneity, which is controlled using luxmeters positioned in the rack supporting the cages of the animals. A second concern is the enrichment that retards photoreceptor degeneration in that model [167]. The most practical solution is to remove the enrichment when the experimental mice are born to ensure that they all become blind by post-natal day (PND) 50. A third problem is the difference in vision loss between gender [168]. It is necessary to use only one sex or, alternatively, to homogeneously distribute males and females in each group of animals. Since the onset of rod degeneration is at PND18 and that the onset of expression of the transgene is about one week long, the mice are injected at PND15 [103,111]. It is essential to use a group of *rd10* animals injected with a non-active AAV (usually encoding green fluorescent protein (GFP)] prepared with the same protocol as negative control [96]). Each mouse receives the treatment in one eye and the contralateral eye is sham operated to distinguish the possible effect of the surgery to be separated from that of the drug [100,169].

After the completion of that study, the clinical grade AAV is produced and used for toxicological and biodistribution studies under good laboratory practices (GLP) conditions. Since the products of the *NXNL1* gene are expressed in the retina of patients prior to the degeneration of photoreceptors, an immune response against the transgene products is not expected. Those studies are done according to regulatory guidelines [170,171,172] and the results are used to request the clinical trial authorization to regulatory bodies. While not mandatory, it is advisable to use non-human primates (NHPs), most often macaques, because among mammals only primates have a fovea [173]. Only laboratories with accreditation can perform such analyses with the objective of demonstrating the absence of toxicity at the therapeutic dose and minimal toxic effect at several times this dose to provide a safety margin. This is usually performed at two different time-points after treatment. Biodistribution studies document the spreading of the recombinant AAV particles into the whole organism which is quite limited after subretinal injection. In addition, the tolerance of the surgical procedure is validated. While it is reasonable that RdCVF will be secreted by RPE-infected cells from the site of injection to the cones within the fovea, the cell-autonomous mechanism of action of RdCVFL under the control of a cone-specific promoter implies that the cones are optimally transduced following subretinal or suprachoroid injection [111,174]. The suprachoroidal space is a potential route to the center of the retina. Subretinal injection creates a temporary bullous detachment, separating the photoreceptor outer segments from the RPE layer. Typically, the subretinal injection bleb resolves over the following few days in humans. Lateral spread adjacent to the initial subretinal injection site of transduction was observed in dogs as a result of a continued detachment of the retina as the bleb flattened out over the first thee3 days post injection [175]. Lateral spread could allow subretinal injection in the parafoveal region to produce transduction of the foveal cells while circumventing the deleterious effects of inducing a foveal detachment. The phenomenon was studied in the dog [175]. Two hundred fifty µl of AAV vector at 5 × 10^11^ vg/mL were injected in the subretinal space in the tapetal region, the *area centralis*, of the fundus of the dog retina. By estimating the size of that bleb using the human retina and the known distance separating the optic nerve head and the fovea it is possible to calculate the volume to be injected.

In order to liberate clinical batches of the drug, they must be qualified using a potency assay that demonstrates that the drug is biologically active. In a research laboratory this is performed using primary cells and animal models that cannot be used within an industrial process. At the early clinical stages, the expression of RdCVF and RdCVFL mRNA can be measured using quantitative RT-PCR and the proteins by sandwich ELISA, but both requires the transduction of an immortalized cell line, cells that can be handled in a confined environment. Since RdCVFL is under control of a cone opsin promoter, only retinoblastoma cell lines that originate from cone precursors are expressing this transgene after their transduction with the AAV expressing RdCVF and RdCVFL [176]. At more advanced stages, the biological activities of both transgenes requires the development of tests after the transduction of the drug in such cell lines.

## 7. Clinical Trial

One of the main aspect of a trial for RP treatment is to recruit a population of patients with a homogenous secondary degeneration of cones [41,177,178,179,180,181,182,183,184]. While the protection of cone function using RdCVF and RdCVFL is predicted to be independent of the causative mutation, in a first stage of a clinical trial recruiting a RP cohort representing a full diversity of mutation in the 65 causative genes reported so far (https://sph.uth.edu/retnet/) may result in an enormous phenotype heterogeneity that will mask any possible benefit for vision after statistical analysis. Since we have demonstrated the positive effects of cone vision of a rodent models of recessive and dominant RP, patients carrying a recessive mutation of the *PDE6B* gene (4% of all recessive RP) and those with dominant mutations in the *RHO* gene (25% of all dominant RP) are better suited for this phase of the project [101]. The cohort will enroll such patients without further stratification. A retrospective study of the visual phenotype of such of patients allow to predict, in addition to the absence of advert effects, what can be expected in terms of benefit and how long after treatment this benefit could be recorded. This study is done to document RP natural history on both anatomical and functional parameters. Even if the mode of action of RdCVF suggests that the cone outer segment could be reconstructed [96], in a more realistic assumption, the prevention of the deconstruction of the cone outer segment following rod cell-death is directly proportional to this rate of deconstruction and of cone vision loss during a given period of time. The retrospective study will be used to evaluate the patients that can benefit from the treatment based on criteria that include the identification of fast progressors. The same cohort will be enrolled in a prospective study for which a subset will received an injection in the macula and peri-macula area of the drug. Even if the first patients will receive a sub-optimal dose for security reasons, the total number of full AAV particles to be injected can be calculated from the efficient dose in the *rd10* mouse in the preclinical study to which must be applied a coefficient calculated by the difference in the number of cones in the mouse and human retina: 113,000 cones in the mouse and 6,400,000 cones in the human retina [165,185]. A low dose will be evaluated on a few severe patients, but an efficient dose will then be evaluated on a cohort of patients with moderate to severe RP. This cohort will exclude patients with no detectable ellipsoid zone that reflects the presence of cone outer segments by spectral domain optical coherence tomography (SD-OCT) [186,187].

The absence of adverse effects and the benefit of the treatment will be assessed by comparing the rate of loss of cone vision of the treated eyes to untreated eyes. The time necessary in the trial to see efficacy may be of several years by measuring visual acuity using kinetic perimetry but other parameters, such as an autofluorescent ring, may evolve faster [188,189]. This should encourage a multicentric and international phase III trial which will be standardized using data on the natural history of the cohorts at each site. An example of other possible complications is the prevalence of the P23H dominant mutation of the rhodopsin gene in North America, while the most prevalent mutation in Europe is P347L [40,41]. Patients with the P347L mutation have an early onset and severe disease, but patients with the P23H mutation are slow progressors [43,190]. This difference will have to be taken in consideration during the evaluation of the benefit of the treatment of RP patients with a recombinant AAV encoding for RdCVF and RdCVFL.

## 8. Conclusions

The dominance of rods in the retina of most mammals is the result of an evolutionary constraint known as nocturnal bottleneck [191]. The reasons explaining why, in RP, rods are affected before cones, even in cases of mutations in genes expressed by both types of photoreceptors, is presently unknown. The secondary loss of function of cones is observed irrespective of the type of mutations. The interruption of the metabolic and redox signaling between rods and cones though the loss of expression of RdCVF following rod cell death explains quite well the phenomenon which is initiated by the reduction of cone vision because of the shortening of the cone outer segments [117,118]. Even if this mechanism may not be unique [192], the administration of both products of the *NXNL1* gene provides the rational for a therapy aimed at preventing or delaying the loss of central vision in all genetic forms of RP.

## 9. Patents

This section is not mandatory, but may be added if there are patents resulting from the work reported in this manuscript.

## Supplementary Materials

The following are available online at https://www.mdpi.com/1422-0067/21/5/1625/s1.

## Abbreviations

AAV	Adeno-associated viral vector
ATP	Adenosine triphosphate
BSG	Basigin
BSG1	Basigin-1 with three immunoglobulin domains
BSG2	Basigin-2 with two immunoglobulin domains
CBA	Chicken beta-actin
cGMP	Cyclic guanosine monophosphate
CMV	Cytomegalovirus
CNTF	Ciliary neurotrophic factor
COS-1	Immortalizing CV-1 cell line
CsCl	Cesium chloride
CSNB	Congenital stationary night blindness
CX3CL1	C-X3-C motif chemokine ligand 1 or fractalkine
DHAP	Dihydroxyacetone phosphate
EMA	European Medicines Agency
ERG	Electroretinogram
FDA	Food and Drug Administration
G3P	Glycerol-3-phosphate
G3PDH	Glycerol-3-phosphate dehydrogenase
G6P	Glucose-6-phosphate
GFP	Green fluorescent protein
GLP	Good laboratory practices
GLUT1	Glucose transporter SLC2A1
GMP	Good manufacturing practice
GNAT1	rod transducin alpha subunit
HEK293	Human embryonic kidney 293 cell line
HDAC4	Histone deacetylase 4
Ig	Immunoglobulin domain
ITRs	Invert terminal repeats
KLF15	Krüppel-like factor 15
LACT	Lactate
LCA	Leber congenital amaurosis
LCA2	Leber congenital amaurosis with a mutation in the RPE65 gene
LCR	Locus control region
LDH	Lactate dehydrogenase
LRAT	Lecithin-retinol acyltransferase
MTCs	Lactate transporters
MPC	Mitochondrial pyruvate carrier
NADPH	Nicotinamide adenine dinucleotide phosphate, reduced
NHPs	Non-human primates
NXNL1	Nucleoredoxin-like 1gene
OPN1L/MW	Long and middle wave opsin gene
OXPHO	Oxidative phosphorylation
PDE6B	Phosphodiesterase 6B
PPP	Pentose phosphate pathway
PRPF31	Pre-mRNA processing factor 31
PUFA	Poly-unsaturated fatty acids
PYR	Pyruvate
qPCR	Quantitative polymerase chain reaction
RB1	Tumor suppressor retinoblastoma 1
*rd1*	Retinal degeneration 1 mutant
RdCVF	Rod-derived cone viability factor
RdCVFL	Thioredoxin enzyme rod-derived cone viability factor long
ROS	Reactive oxygen species
RP	Retinitis pigmentosa
RPE	Retinal pigmented epithelium
RPE65	Retinal pigment epithelium protein of 65 kDa
SD-OCT	Spectral domain optical coherence tomography
SV40T	Simian virus 40 large T antigen
TCA	Tricarboxylic acid cycle
TXNRD	Thioredoxin reductase

## References

[1] Takahashi V.K.L., Takiuti J.T., Jauregui R., Tsang S.H. (2018). Gene therapy in inherited retinal degenerative diseases, a review. Ophthalmic Genet..

[2] Kumaran N., Michaelides M., Smith A.J., Ali R.R., Bainbridge J.W.B. (2018). Retinal gene therapy. Br. Med. Bull..

[3] Trapani I., Auricchio A. (2019). Has retinal gene therapy come of age? From bench to bedside and back to bench. Hum. Mol. Genet..

[4] Apte R.S. (2018). Gene Therapy for Retinal Degeneration. Cell.

[5] Kaiser J. (2011). Clinical research. Gene therapists celebrate a decade of progress. Science.

[6] Hamel C.P., Tsilou E., Pfeffer B.A., Hooks J.J., Detrick B., Redmond T.M. (1993). Molecular cloning and expression of RPE65, a novel retinal pigment epithelium-specific microsomal protein that is post-transcriptionally regulated in vitro. J. Biol. Chem..

[7] Marlhens F., Bareil C., Griffoin J.M., Zrenner E., Amalric P., Eliaou C., Liu S.Y., Harris E., Redmond T.M., Arnaud B. (1997). Mutations in RPE65 cause Leber’s congenital amaurosis. Nat. Genet..

[8] Redmond T.M., Yu S., Lee E., Bok D., Hamasaki D., Chen N., Goletz P., Ma J.X., Crouch R.K., Pfeifer K. (1998). Rpe65 is necessary for production of 11-cis-vitamin A in the retinal visual cycle. Nat. Genet..

[9] Aguirre G.D., Baldwin V., Pearce-Kelling S., Narfstrom K., Ray K., Acland G.M. (1998). Congenital stationary night blindness in the dog: Common mutation in the RPE65 gene indicates founder effect. Mol. Vis..

[10] Acland G.M., Aguirre G.D., Ray J., Zhang Q., Aleman T.S., Cideciyan A.V., Pearce-Kelling S.E., Anand V., Zeng Y., Maguire A.M. (2001). Gene therapy restores vision in a canine model of childhood blindness. Nat. Genet..

[11] Lehrman S. (1999). Virus treatment questioned after gene therapy death. Nature.

[12] Marshall E. (1999). Gene therapy death prompts review of adenovirus vector. Science.

[13] Barbour V. (2000). The balance of risk and benefit in gene-therapy trials. Lancet.

[14] Maguire A.M., Simonelli F., Pierce E.A., Pugh E.N., Mingozzi F., Bennicelli J., Banfi S., Marshall K.A., Testa F., Surace E.M. (2008). Safety and efficacy of gene transfer for Leber’s congenital amaurosis. N. Engl. J. Med..

[15] Bainbridge J.W., Smith A.J., Barker S.S., Robbie S., Henderson R., Balaggan K., Viswanathan A., Holder G.E., Stockman A., Tyler N. (2008). Effect of gene therapy on visual function in Leber’s congenital amaurosis. N. Engl. J. Med..

[16] Cideciyan A.V., Aleman T.S., Boye S.L., Schwartz S.B., Kaushal S., Roman A.J., Pang J.J., Sumaroka A., Windsor E.A., Wilson J.M. (2008). Human gene therapy for RPE65 isomerase deficiency activates the retinoid cycle of vision but with slow rod kinetics. Proc. Natl. Acad. Sci. USA.

[17] Ledford H. (2015). Success against blindness encourages gene therapy researchers. Nature.

[18] Bennett J., Ashtari M., Wellman J., Marshall K.A., Cyckowski L.L., Chung D.C., McCague S., Pierce E.A., Chen Y., Bennicelli J.L. (2012). AAV2 gene therapy readministration in three adults with congenital blindness. Sci. Transl. Med..

[19] Cideciyan A.V., Jacobson S.G., Beltran W.A., Sumaroka A., Swider M., Iwabe S., Roman A.J., Olivares M.B., Schwartz S.B., Komaromy A.M. (2013). Human retinal gene therapy for Leber congenital amaurosis shows advancing retinal degeneration despite enduring visual improvement. Proc. Natl. Acad. Sci. USA.

[20] Jacobson S.G., Cideciyan A.V., Roman A.J., Sumaroka A., Schwartz S.B., Heon E., Hauswirth W.W. (2015). Improvement and decline in vision with gene therapy in childhood blindness. N. Engl. J. Med..

[21] Bainbridge J.W., Mehat M.S., Sundaram V., Robbie S.J., Barker S.E., Ripamonti C., Georgiadis A., Mowat F.M., Beattie S.G., Gardner P.J. (2015). Long-term effect of gene therapy on Leber’s congenital amaurosis. N. Engl. J. Med..

[22] Boye S.E., Huang W.C., Roman A.J., Sumaroka A., Boye S.L., Ryals R.C., Olivares M.B., Ruan Q., Tucker B.A., Stone E.M. (2014). Natural history of cone disease in the murine model of Leber congenital amaurosis due to CEP290 mutation: Determining the timing and expectation of therapy. PLoS ONE.

[23] Beltran W.A., Cideciyan A.V., Iwabe S., Swider M., Kosyk M.S., McDaid K., Martynyuk I., Ying G.S., Shaffer J., Deng W.T. (2015). Successful arrest of photoreceptor and vision loss expands the therapeutic window of retinal gene therapy to later stages of disease. Proc. Natl. Acad. Sci. USA.

[24] Wright C.B., Redmond T.M., Nickerson J.M. (2015). A History of the Classical Visual Cycle. Prog. Mol. Biol. Transl. Sci..

[25] Caruso R.C., Aleman T.S., Cideciyan A.V., Roman A.J., Sumaroka A., Mullins C.L., Boye S.L., Hauswirth W.W., Jacobson S.G. (2010). Retinal disease in Rpe65-deficient mice: Comparison to human leber congenital amaurosis due to RPE65 mutations. Investig. Ophthalmol. Vis. Sci..

[26] Zhang T., Zhang N., Baehr W., Fu Y. (2011). Cone opsin determines the time course of cone photoreceptor degeneration in Leber congenital amaurosis. Proc. Natl. Acad. Sci. USA.

[27] Russell S., Bennett J., Wellman J.A., Chung D.C., Yu Z.F., Tillman A., Wittes J., Pappas J., Elci O., McCague S. (2017). Efficacy and safety of voretigene neparvovec (AAV2-hRPE65v2) in patients with RPE65-mediated inherited retinal dystrophy: A randomised, controlled, open-label, phase 3 trial. Lancet.

[28] Frison-Roche R. (1941). Premier de Cordée: Roman.

[29] Darrow J.J. (2019). Luxturna: FDA documents reveal the value of a costly gene therapy. Drug Discov. Today.

[30] Blond F., Leveillard T. (2019). Functional Genomics of the Retina to Elucidate its Construction and Deconstruction. Int. J. Mol. Sci..

[31] Vithana E.N., Abu-Safieh L., Allen M.J., Carey A., Papaioannou M., Chakarova C., Al-Maghtheh M., Ebenezer N.D., Willis C., Moore A.T. (2001). A human homolog of yeast pre-mRNA splicing gene, PRP31, underlies autosomal dominant retinitis pigmentosa on chromosome 19q13.4 (RP11). Mol. Cell.

[32] Rose A.M., Bhattacharya S.S. (2016). Variant haploinsufficiency and phenotypic non-penetrance in PRPF31-associated retinitis pigmentosa. Clin. Genet..

[33] Dryja T.P., McGee T.L., Hahn L.B., Cowley G.S., Olsson J.E., Reichel E., Sandberg M.A., Berson E.L. (1990). Mutations within the rhodopsin gene in patients with autosomal dominant retinitis pigmentosa. N. Engl. J. Med..

[34] Mendes H.F., van der Spuy J., Chapple J.P., Cheetham M.E. (2005). Mechanisms of cell death in rhodopsin retinitis pigmentosa: Implications for therapy. Trends Mol. Med..

[35] Gorbatyuk M.S., Knox T., LaVail M.M., Gorbatyuk O.S., Noorwez S.M., Hauswirth W.W., Lin J.H., Muzyczka N., Lewin A.S. (2010). Restoration of visual function in P23H rhodopsin transgenic rats by gene delivery of BiP/Grp78. Proc. Natl. Acad. Sci. USA.

[36] Lewin A.S., Drenser K.A., Hauswirth W.W., Nishikawa S., Yasumura D., Flannery J.G., LaVail M.M. (1998). Ribozyme rescue of photoreceptor cells in a transgenic rat model of autosomal dominant retinitis pigmentosa. Nat. Med..

[37] Phylactou L.A., Kilpatrick M.W., Wood M.J. (1998). Ribozymes as therapeutic tools for genetic disease. Hum. Mol. Genet..

[38] Hauswirth W.W., Lewin A.S. (2000). Ribozyme uses in retinal gene therapy. Prog. Retin. Eye Res..

[39] Malanson K.M., Lem J. (2009). Rhodopsin-mediated retinitis pigmentosa. Prog. Mol. Biol. Transl. Sci..

[40] Audo I., Manes G., Mohand-Said S., Friedrich A., Lancelot M.E., Antonio A., Moskova-Doumanova V., Poch O., Zanlonghi X., Hamel C.P. (2010). Spectrum of rhodopsin mutations in French autosomal dominant rod-cone dystrophy patients. Investig. Ophthalmol. Vis. Sci..

[41] Fernandez-San Jose P., Blanco-Kelly F., Corton M., Trujillo-Tiebas M.J., Gimenez A., Avila-Fernandez A., Garcia-Sandoval B., Lopez-Molina M.I., Hernan I., Carballo M. (2015). Prevalence of Rhodopsin mutations in autosomal dominant Retinitis Pigmentosa in Spain: Clinical and analytical review in 200 families. Acta Ophthalmol..

[42] Oh K.T., Longmuir R., Oh D.M., Stone E.M., Kopp K., Brown J., Fishman G.A., Sonkin P., Gehrs K.M., Weleber R.G. (2003). Comparison of the clinical expression of retinitis pigmentosa associated with rhodopsin mutations at codon 347 and codon 23. Am. J. Ophthalmol..

[43] Bonilha V.L., Rayborn M.E., Bell B.A., Marino M.J., Beight C.D., Pauer G.J., Traboulsi E.I., Hollyfield J.G., Hagstrom S.A. (2015). Retinal histopathology in eyes from patients with autosomal dominant retinitis pigmentosa caused by rhodopsin mutations. Graefe’s Arch. Clin. Exp. Ophthalmol..

[44] O’Reilly M., Palfi A., Chadderton N., Millington-Ward S., Ader M., Cronin T., Tuohy T., Auricchio A., Hildinger M., Tivnan A. (2007). RNA interference-mediated suppression and replacement of human rhodopsin in vivo. Am. J. Hum. Genet..

[45] Cideciyan A.V., Sudharsan R., Dufour V.L., Massengill M.T., Iwabe S., Swider M., Lisi B., Sumaroka A., Marinho L.F., Appelbaum T. (2018). Mutation-independent rhodopsin gene therapy by knockdown and replacement with a single AAV vector. Proc. Natl. Acad. Sci. USA.

[46] Kiang A.S., Palfi A., Ader M., Kenna P.F., Millington-Ward S., Clark G., Kennan A., O’Reilly M., Tam L.C., Aherne A. (2005). Toward a gene therapy for dominant disease: Validation of an RNA interference-based mutation-independent approach. Mol. Ther. J. Am. Soc. Gene Ther..

[47] Botta S., de Prisco N., Marrocco E., Renda M., Sofia M., Curion F., Bacci M.L., Ventrella D., Wilson C., Gesualdo C. (2017). Targeting and silencing of rhodopsin by ectopic expression of the transcription factor KLF15. JCI Insight.

[48] Suzuki K., Tsunekawa Y., Hernandez-Benitez R., Wu J., Zhu J., Kim E.J., Hatanaka F., Yamamoto M., Araoka T., Li Z. (2016). In vivo genome editing via CRISPR/Cas9 mediated homology-independent targeted integration. Nature.

[49] Vagni P., Perlini L.E., Chenais N.A.L., Marchetti T., Parrini M., Contestabile A., Cancedda L., Ghezzi D. (2019). Gene Editing Preserves Visual Functions in a Mouse Model of Retinal Degeneration. Front. Neurosci..

[50] Moreno A.M., Fu X., Zhu J., Katrekar D., Shih Y.-R.V., Marlett J., Cabotaje J., Tat J., Naughton J., Lisowski L. (2018). In Situ Gene Therapy via AAV-CRISPR-Cas9-Mediated Targeted Gene Regulation. Mol. Ther. J. Am. Soc. Gene Ther..

[51] Samardzija M., Wenzel A., Thiersch M., Frigg R., Reme C., Grimm C. (2006). Caspase-1 ablation protects photoreceptors in a model of autosomal dominant retinitis pigmentosa. Investig. Ophthalmol. Vis. Sci..

[52] Marigo V. (2007). Programmed cell death in retinal degeneration: Targeting apoptosis in photoreceptors as potential therapy for retinal degeneration. Cell Cycle.

[53] Guo X., Wang S.B., Xu H., Ribic A., Mohns E.J., Zhou Y., Zhu X., Biederer T., Crair M.C., Chen B. (2015). A short N-terminal domain of HDAC4 preserves photoreceptors and restores visual function in retinitis pigmentosa. Nat. Commun..

[54] Lipinski D.M., Barnard A.R., Singh M.S., Martin C., Lee E.J., Davies W.I.L., MacLaren R.E. (2015). CNTF Gene Therapy Confers Lifelong Neuroprotection in a Mouse Model of Human Retinitis Pigmentosa. Mol. Ther. J. Am. Soc. Gene Ther..

[55] Birch D.G., Bennett L.D., Duncan J.L., Weleber R.G., Pennesi M.E. (2016). Long-term Follow-up of Patients with Retinitis Pigmentosa Receiving Intraocular Ciliary Neurotrophic Factor Implants. Am. J. Ophthalmol..

[56] Karali M., Guadagnino I., Marrocco E., De Cegli R., Carissimo A., Pizzo M., Casarosa S., Conte I., Surace E.M., Banfi S. (2019). AAV-miR-204 Protects from Retinal Degeneration by Attenuation of Microglia Activation and Photoreceptor Cell Death. Mol. Ther. Nucleic Acids.

[57] Wang S.K., Xue Y., Rana P., Hong C.M., Cepko C.L. (2019). Soluble CX3CL1 gene therapy improves cone survival and function in mouse models of retinitis pigmentosa. Proc. Natl. Acad. Sci. USA.

[58] Xiong W., MacColl Garfinkel A.E., Li Y., Benowitz L.I., Cepko C.L. (2015). NRF2 promotes neuronal survival in neurodegeneration and acute nerve damage. J. Clin. Investig..

[59] Picard E., Jonet L., Sergeant C., Vesvres M.-H., Behar-Cohen F., Courtois Y., Jeanny J.-C. (2010). Overexpressed or intraperitoneally injected human transferrin prevents photoreceptor degeneration in rd10 mice. Mol. Vis..

[60] Petit L., Punzo C. (2015). mTORC1 sustains vision in retinitis pigmentosa. Oncotarget.

[61] Zhang L., Du J., Justus S., Hsu C.-W., Bonet-Ponce L., Wu W.-H., Tsai Y.-T., Wu W.-P., Jia Y., Duong J.K. (2016). Reprogramming metabolism by targeting sirtuin 6 attenuates retinal degeneration. J. Clin. Investig..

[62] Portera-Cailliau C., Sung C.H., Nathans J., Adler R. (1994). Apoptotic photoreceptor cell death in mouse models of retinitis pigmentosa. Proc. Natl. Acad. Sci. USA.

[63] Hartong D.T., Berson E.L., Dryja T.P. (2006). Retinitis pigmentosa. Lancet.

[64] Snyder C. (1963). Jean Nougaret, the butcher from Provence, and his family. Arch. Ophthalmol..

[65] Dryja T.P., Hahn L.B., Reboul T., Arnaud B. (1996). Missense mutation in the gene encoding the alpha subunit of rod transducin in the Nougaret form of congenital stationary night blindness. Nat. Genet..

[66] Rosenberg T., Haim M., Piczenik Y., Simonsen S.E. (1991). Autosomal Dominant Stationary Night-Blindness—A Large Family Rediscovered. Acta Ophthalmol..

[67] Zeitz C., Robson A.G., Audo I. (2015). Congenital stationary night blindness: An analysis and update of genotype-phenotype correlations and pathogenic mechanisms. Prog. Retin. Eye Res..

[68] Wright A.F. (1997). A searchlight through the fog. Nat. Genet..

[69] Kaplan H.J., Wang W., Dean D.C. (2017). Restoration of Cone Photoreceptor Function in Retinitis Pigmentosa. Transl. Vis. Sci. Technol..

[70] Geller A.M., Sieving P.A. (1993). Assessment of foveal cone photoreceptors in Stargardt’s macular dystrophy using a small dot detection task. Vis. Res..

[71] Petersen-Jones S.M., Occelli L.M., Winkler P.A., Lee W., Sparrow J.R., Tsukikawa M., Boye S.L., Chiodo V., Capasso J.E., Becirovic E. (2017). Patients and animal models of CNGβ1-deficient retinitis pigmentosa support gene augmentation approach. J. Clin. Investig..

[72] Cronin T., Leveillard T., Sahel J.A. (2007). Retinal degenerations: From cell signaling to cell therapy; pre-clinical and clinical issues. Curr. Gene Ther..

[73] Applebury M.L. (1990). Molecular-Genetics—Insight into Blindness. Nature.

[74] Daiger S.P., Humphries M.M., Giesenschlag N., Sharp E., McWilliam P., Farrer J., Bradley D., Kenna P., McConnell D.J., Sparkes R.S. (1989). Linkage analysis of human chromosome 4: Exclusion of autosomal dominant retinitis pigmentosa (ADRP) and detection of new linkage groups. Cytogenet. Cell Genet..

[75] Dryja T.P., McGee T.L., Reichel E., Hahn L.B., Cowley G.S., Yandell D.W., Sandberg M.A., Berson E.L. (1990). A point mutation of the rhodopsin gene in one form of retinitis pigmentosa. Nature.

[76] Kolb H., Gouras P. (1974). Electron microscopic observations of human retinitis pigmentosa, dominantly inherited. Investig. Ophthalmol..

[77] Berson E.L. (1990). Ocular findings in a form of retinitis pigmentosa with a rhodopsin gene defect. Trans. Am. Ophthalmol. Soc..

[78] Cideciyan A.V., Hood D.C., Huang Y., Banin E., Li Z.Y., Stone E.M., Milam A.H., Jacobson S.G. (1998). Disease sequence from mutant rhodopsin allele to rod and cone photoreceptor degeneration in man. Proc. Natl. Acad. Sci. USA.

[79] Carter-Dawson L.D., LaVail M.M., Sidman R.L. (1978). Differential effect of the rd mutation on rods and cones in the mouse retina. Investig. Ophthalmol. Vis. Sci..

[80] Bowes C., Li T., Danciger M., Baxter L.C., Applebury M.L., Farber D.B. (1990). Retinal degeneration in the rd mouse is caused by a defect in the beta subunit of rod cGMP-phosphodiesterase. Nature.

[81] McLaughlin M.E., Sandberg M.A., Berson E.L., Dryja T.P. (1993). Recessive mutations in the gene encoding the beta-subunit of rod phosphodiesterase in patients with retinitis pigmentosa. Nat. Genet..

[82] Usukura J., Khoo W., Abe T., Breitman M.L., Shinohara T. (1994). Cone cells fail to develop normally in transgenic mice showing ablation of rod photoreceptor cells. Cell Tissue Res..

[83] McCall M.A., Gregg R.G., Merriman K., Goto Y., Peachey N.S., Stanford L.R. (1996). Morphological and physiological consequences of the selective elimination of rod photoreceptors in transgenic mice. Exp. Eye Res..

[84] Scott P.A., de Castro J.P., DeMarco P.J., Ross J.W., Njoka J., Walters E., Prather R.S., McCall M.A., Kaplan H.J. (2017). Progression of Pro23His Retinopathy in a Miniature Swine Model of Retinitis Pigmentosa. Transl. Vis. Sci. Technol..

[85] Mieziewska K., Van Veen T., Aguirre G.D. (1993). Development and fate of interphotoreceptor matrix components during dysplastic photoreceptor differentiation: A lectin cytochemical study of rod-cone dysplasia 1. Exp. Eye Res..

[86] Choi R.Y., Engbretson G.A., Solessio E.C., Jones G.A., Coughlin A., Aleksic I., Zuber M.E. (2011). Cone degeneration following rod ablation in a reversible model of retinal degeneration. Investig. Ophthalmol. Vis. Sci..

[87] Punzo C., Kornacker K., Cepko C.L. (2009). Stimulation of the insulin/mTOR pathway delays cone death in a mouse model of retinitis pigmentosa. Nat. Neurosci..

[88] Mohand-Said S., Hicks D., Simonutti M., Tran-Minh D., Deudon-Combe A., Dreyfus H., Silverman M.S., Ogilvie J.M., Tenkova T., Sahel J. (1997). Photoreceptor transplants increase host cone survival in the retinal degeneration (rd) mouse. Ophthalmic Res..

[89] Mohand-Said S., Hicks D., Dreyfus H., Sahel J.A. (2000). Selective transplantation of rods delays cone loss in a retinitis pigmentosa model. Arch. Ophthalmol..

[90] Mohand-Said S., Deudon-Combe A., Hicks D., Simonutti M., Forster V., Fintz A.C., Leveillard T., Dreyfus H., Sahel J.A. (1998). Normal retina releases a diffusible factor stimulating cone survival in the retinal degeneration mouse. Proc. Natl. Acad. Sci. USA.

[91] Fintz A.C., Audo I., Hicks D., Mohand-Said S., Leveillard T., Sahel J. (2003). Partial characterization of retina-derived cone neuroprotection in two culture models of photoreceptor degeneration. Investig. Ophthalmol. Vis. Sci..

[92] Adler R., Hatlee M. (1989). Plasticity and differentiation of embryonic retinal cells after terminal mitosis. Science.

[93] Hewitt A.T., Lindsey J.D., Carbott D., Adler R. (1990). Photoreceptor survival-promoting activity in interphotoreceptor matrix preparations: Characterization and partial purification. Exp. Eye Res..

[94] Weleber R.G., Gregory-Evans K., Hinton D.R., Schachat A.P., Wilkinson C.P. (2006). Retinitis Pigmentosa and Allied Disorders A2—Ryan, Stephen, J. Retina.

[95] Leveillard T., Mohand-Said S., Lorentz O., Hicks D., Fintz A.C., Clerin E., Simonutti M., Forster V., Cavusoglu N., Chalmel F. (2004). Identification and characterization of rod-derived cone viability factor. Nat. Genet..

[96] Ait-Ali N., Fridlich R., Millet-Puel G., Clerin E., Delalande F., Jaillard C., Blond F., Perrocheau L., Reichman S., Byrne L.C. (2015). Rod-derived cone viability factor promotes cone survival by stimulating aerobic glycolysis. Cell.

[97] Lambard S., Reichman S., Berlinicke C., Niepon M.L., Goureau O., Sahel J.A., Leveillard T., Zack D.J. (2010). Expression of rod-derived cone viability factor: Dual role of CRX in regulating promoter activity and cell-type specificity. PLoS ONE.

[98] Reichman S., Kalathur R.K., Lambard S., Ait-Ali N., Yang Y., Lardenois A., Ripp R., Poch O., Zack D.J., Sahel J.A. (2010). The homeobox gene CHX10/VSX2 regulates RdCVF promoter activity in the inner retina. Hum. Mol. Genet..

[99] Delyfer M.N., Raffelsberger W., Mercier D., Korobelnik J.F., Gaudric A., Charteris D.G., Tadayoni R., Metge F., Caputo G., Barale P.O. (2011). Transcriptomic analysis of human retinal detachment reveals both inflammatory response and photoreceptor death. PLoS ONE.

[100] Yang Y., Mohand-Said S., Danan A., Simonutti M., Fontaine V., Clerin E., Picaud S., Leveillard T., Sahel J.A. (2009). Functional cone rescue by RdCVF protein in a dominant model of retinitis pigmentosa. Mol. Ther. J. Am. Soc. Gene Ther..

[101] Leveillard T., Sahel J.A. (2010). Rod-derived cone viability factor for treating blinding diseases: From clinic to redox signaling. Sci. Transl. Med..

[102] Sahel J.A., Leveillard T., Picaud S., Dalkara D., Marazova K., Safran A., Paques M., Duebel J., Roska B., Mohand-Said S. (2013). Functional rescue of cone photoreceptors in retinitis pigmentosa. Graefe’s Arch. Clin. Exp. Ophthalmol..

[103] Byrne L.C., Dalkara D., Luna G., Fisher S.K., Clerin E., Sahel J.A., Leveillard T., Flannery J.G. (2015). Viral-mediated RdCVF and RdCVFL expression protects cone and rod photoreceptors in retinal degeneration. J. Clin. Investig..

[104] Ochrietor J.D., Moroz T.P., van Ekeris L., Clamp M.F., Jefferson S.C., deCarvalho A.C., Fadool J.M., Wistow G., Muramatsu T., Linser P.J. (2003). Retina-specific expression of 5A11/Basigin-2, a member of the immunoglobulin gene superfamily. Investig. Ophthalmol. Vis. Sci..

[105] Leveillard T. (2015). Cancer metabolism of cone photoreceptors. Oncotarget.

[106] Chinchore Y., Begaj T., Wu D., Drokhlyansky E., Cepko C.L. (2017). Glycolytic reliance promotes anabolism in photoreceptors. eLife.

[107] Chalmel F., Leveillard T., Jaillard C., Lardenois A., Berdugo N., Morel E., Koehl P., Lambrou G., Holmgren A., Sahel J.A. (2007). Rod-derived Cone Viability Factor-2 is a novel bifunctional-thioredoxin-like protein with therapeutic potential. BMC Mol. Biol..

[108] Brennan L.A., Lee W., Kantorow M. (2010). TXNL6 is a novel oxidative stress-induced reducing system for methionine sulfoxide reductase a repair of alpha-crystallin and cytochrome C in the eye lens. PLoS ONE.

[109] Cronin T., Raffelsberger W., Lee-Rivera I., Jaillard C., Niepon M.L., Kinzel B., Clerin E., Petrosian A., Picaud S., Poch O. (2010). The disruption of the rod-derived cone viability gene leads to photoreceptor dysfunction and susceptibility to oxidative stress. Cell Death Differ..

[110] Sahel J. (2016). Metabolic and redox signaling in the retina. Cell. Mol. Life Sci. CMLS.

[111] Mei X., Chaffiol A., Kole C., Yang Y., Millet-Puel G., Clerin E., Ait-Ali N., Bennett J., Dalkara D., Sahel J.A. (2016). The Thioredoxin Encoded by the Rod-Derived Cone Viability Factor Gene Protects Cone Photoreceptors Against Oxidative Stress. Antioxid. Redox Signal..

[112] Anastasiou D., Poulogiannis G., Asara J.M., Boxer M.B., Jiang J.K., Shen M., Bellinger G., Sasaki A.T., Locasale J.W., Auld D.S. (2011). Inhibition of pyruvate kinase M2 by reactive oxygen species contributes to cellular antioxidant responses. Science.

[113] Hildebrandt T., Knuesting J., Berndt C., Morgan B., Scheibe R. (2015). Cytosolic thiol switches regulating basic cellular functions: GAPDH as an information hub?. Biol. Chem..

[114] Lopez-Grueso M.J., Gonzalez-Ojeda R., Requejo-Aguilar R., McDonagh B., Fuentes-Almagro C.A., Muntane J., Barcena J.A., Padilla C.A. (2019). Thioredoxin and glutaredoxin regulate metabolism through different multiplex thiol switches. Redox Biol..

[115] Ren X., Zou L., Lu J., Holmgren A. (2018). Selenocysteine in mammalian thioredoxin reductase and application of ebselen as a therapeutic. Free Radic. Biol. Med..

[116] Camacho E.T., Brager D., Elachouri G., Korneyeva T., Millet-Puel G., Sahel J.A., Leveillard T. (2019). A Mathematical Analysis of Aerobic Glycolysis Triggered by Glucose Uptake in Cones. Sci. Rep..

[117] Leveillard T., Sahel J.A. (2017). Metabolic and redox signaling in the retina. Cell. Mol. Life Sci. CMLS.

[118] Leveillard T., Ait-Ali N. (2017). Cell Signaling with Extracellular Thioredoxin and Thioredoxin-Like Proteins: Insight into Their Mechanisms of Action. Oxidative Med. Cell. Longev..

[119] Domenger C., Grimm D. (2019). Next-generation AAV vectors-do not judge a virus (only) by its cover. Hum. Mol. Genet..

[120] Martinez-Fernandez De La Camara C., Cehajic-Kapetanovic J., MacLaren R.E. (2019). RPGR gene therapy presents challenges in cloning the coding sequence. Expert Opin. Biol. Ther..

[121] Bell P., Wang L., Chen S.J., Yu H., Zhu Y., Nayal M., He Z., White J., Lebel-Hagan D., Wilson J.M. (2016). Effects of Self-Complementarity, Codon Optimization, Transgene, and Dose on Liver Transduction with AAV8. Hum. Gene Ther. Methods.

[122] Dai Y., Roman M., Naviaux R.K., Verma I.M. (1992). Gene therapy via primary myoblasts: Long-term expression of factor IX protein following transplantation in vivo. Proc. Natl. Acad. Sci. USA.

[123] Miyazaki J., Takaki S., Araki K., Tashiro F., Tominaga A., Takatsu K., Yamamura K. (1989). Expression vector system based on the chicken beta-actin promoter directs efficient production of interleukin-5. Gene.

[124] Elachouri G., Lee-Rivera I., Clerin E., Argentini M., Fridlich R., Blond F., Ferracane V., Yang Y., Raffelsberger W., Wan J. (2015). Thioredoxin rod-derived cone viability factor protects against photooxidative retinal damage. Free Radic. Biol. Med..

[125] Zack D.J., Bennett J., Wang Y., Davenport C., Klaunberg B., Gearhart J., Nathans J. (1991). Unusual topography of bovine rhodopsin promoter-lacZ fusion gene expression in transgenic mouse retinas. Neuron.

[126] Mancuso K., Hauswirth W.W., Li Q., Connor T.B., Kuchenbecker J.A., Mauck M.C., Neitz J., Neitz M. (2009). Gene therapy for red-green colour blindness in adult primates. Nature.

[127] Tian J., Andreadis S.T. (2009). Independent and high-level dual-gene expression in adult stem-progenitor cells from a single lentiviral vector. Gene Ther..

[128] Nojima T., Dienstbier M., Murphy S., Proudfoot N.J., Dye M.J. (2013). Definition of RNA polymerase II CoTC terminator elements in the human genome. Cell Rep..

[129] Parrish C., Berns K. (2007). Parvoviridae. Fields Virology.

[130] Dong J.Y., Fan P.D., Frizzell R.A. (1996). Quantitative analysis of the packaging capacity of recombinant adeno-associated virus. Hum. Gene Ther..

[131] Grieger J.C., Samulski R.J. (2005). Packaging capacity of adeno-associated virus serotypes: Impact of larger genomes on infectivity and postentry steps. J. Virol..

[132] Wu Z., Yang H., Colosi P. (2010). Effect of genome size on AAV vector packaging. Mol. Ther. J. Am. Soc. Gene Ther..

[133] Bennicelli J., Wright J.F., Komaromy A., Jacobs J.B., Hauck B., Zelenaia O., Mingozzi F., Hui D., Chung D., Rex T.S. (2008). Reversal of blindness in animal models of leber congenital amaurosis using optimized AAV2-mediated gene transfer. Mol. Ther. J. Am. Soc. Gene Ther..

[134] Cheng S.W., Court D.L., Friedman D.I. (1995). Transcription termination signals in the nin region of bacteriophage lambda: Identification of Rho-dependent termination regions. Genetics.

[135] Chadeuf G., Ciron C., Moullier P., Salvetti A. (2005). Evidence for encapsidation of prokaryotic sequences during recombinant adeno-associated virus production and their in vivo persistence after vector delivery. Mol. Ther. J. Am. Soc. Gene Ther..

[136] Wang X.S., Qing K., Ponnazhagan S., Srivastava A. (1997). Adeno-associated virus type 2 DNA replication in vivo: Mutation analyses of the D sequence in viral inverted terminal repeats. J. Virol..

[137] Kole C., Klipfel L., Yang Y., Ferracane V., Blond F., Reichman S., Millet-Puel G., Clerin E., Ait-Ali N., Pagan D. (2018). Otx2-Genetically Modified Retinal Pigment Epithelial Cells Rescue Photoreceptors after Transplantation. Mol. Ther. J. Am. Soc. Gene Ther..

[138] Schmeer M., Buchholz T., Schleef M. (2017). Plasmid DNA Manufacturing for Indirect and Direct Clinical Applications. Hum. Gene Ther..

[139] Penaud-Budloo M., Francois A., Clement N., Ayuso E. (2018). Pharmacology of Recombinant Adeno-associated Virus Production. Mol. Ther. Methods Clin. Dev..

[140] Lebherz C., Maguire A., Tang W., Bennett J., Wilson J.M. (2008). Novel AAV serotypes for improved ocular gene transfer. J. Gene Med..

[141] Naso M.F., Tomkowicz B., Perry W.L., Strohl W.R. (2017). Adeno-Associated Virus (AAV) as a Vector for Gene Therapy. Biodrugs Clin. Immunother. Biopharm. Gene Ther..

[142] Vandenberghe L.H., Bell P., Maguire A.M., Xiao R., Hopkins T.B., Grant R., Bennett J., Wilson J.M. (2013). AAV9 targets cone photoreceptors in the nonhuman primate retina. PLoS ONE.

[143] Boye S.E., Alexander J.J., Boye S.L., Witherspoon C.D., Sandefer K.J., Conlon T.J., Erger K., Sun J., Ryals R., Chiodo V.A. (2012). The human rhodopsin kinase promoter in an AAV5 vector confers rod- and cone-specific expression in the primate retina. Hum. Gene Ther..

[144] Virag T., Cecchini S., Kotin R.M. (2009). Producing recombinant adeno-associated virus in foster cells: Overcoming production limitations using a baculovirus-insect cell expression strategy. Hum. Gene Ther..

[145] Kotin R.M., Snyder R.O. (2017). Manufacturing Clinical Grade Recombinant Adeno-Associated Virus Using Invertebrate Cell Lines. Hum. Gene Ther..

[146] Kondratov O., Marsic D., Crosson S.M., Mendez-Gomez H.R., Moskalenko O., Mietzsch M., Heilbronn R., Allison J.R., Green K.B., Agbandje-McKenna M. (2017). Direct Head-to-Head Evaluation of Recombinant Adeno-associated Viral Vectors Manufactured in Human versus Insect Cells. Mol. Ther. J. Am. Soc. Gene Ther..

[147] Robert M.A., Chahal P.S., Audy A., Kamen A., Gilbert R., Gaillet B. (2017). Manufacturing of recombinant adeno-associated viruses using mammalian expression platforms. Biotechnol. J..

[148] Graham F.L., Smiley J., Russell W.C., Nairn R. (1977). Characteristics of a human cell line transformed by DNA from human adenovirus type 5. J. Gen. Virol..

[149] Egan C., Bayley S.T., Branton P.E. (1989). Binding of the Rb1 protein to E1A products is required for adenovirus transformation. Oncogene.

[150] Hacker D.L., Bertschinger M., Baldi L., Wurm F.M. (2004). Reduction of adenovirus E1A mRNA by RNAi results in enhanced recombinant protein expression in transiently transfected HEK293 cells. Gene.

[151] Pear W.S., Nolan G.P., Scott M.L., Baltimore D. (1993). Production of high-titer helper-free retroviruses by transient transfection. Proc. Natl. Acad. Sci. USA.

[152] Lin J.Y., Simmons D.T. (1991). The ability of large T antigen to complex with p53 is necessary for the increased life span and partial transformation of human cells by simian virus 40. J. Virol..

[153] Emmerling V.V., Pegel A., Milian E.G., Venereo-Sanchez A., Kunz M., Wegele J., Kamen A.A., Kochanek S., Hoerer M. (2016). Rational plasmid design and bioprocess optimization to enhance recombinant adeno-associated virus (AAV) productivity in mammalian cells. Biotechnol. J..

[154] World Health Organization (2006). WHO Guidelines on Tissue Infectivity Distribution in Transmissible Spongiform Encephalopathies.

[155] Strobel B., Miller F.D., Rist W., Lamla T. (2015). Comparative Analysis of Cesium Chloride- and Iodixanol-Based Purification of Recombinant Adeno-Associated Viral Vectors for Preclinical Applications. Hum. Gene Ther. Methods.

[156] Nass S.A., Mattingly M.A., Woodcock D.A., Burnham B.L., Ardinger J.A., Osmond S.E., Frederick A.M., Scaria A., Cheng S.H., O’Riordan C.R. (2018). Universal Method for the Purification of Recombinant AAV Vectors of Differing Serotypes. Mol. Ther. Methods Clin. Dev..

[157] Wang C., Mulagapati S.H.R., Chen Z., Du J., Zhao X., Xi G., Chen L., Linke T., Gao C., Schmelzer A.E. (2019). Developing an Anion Exchange Chromatography Assay for Determining Empty and Full Capsid Contents in AAV6.2. Mol. Ther. Methods Clin. Dev..

[158] Burnham B., Nass S., Kong E., Mattingly M., Woodcock D., Song A., Wadsworth S., Cheng S.H., Scaria A., O’Riordan C.R. (2015). Analytical Ultracentrifugation as an Approach to Characterize Recombinant Adeno-Associated Viral Vectors. Hum. Gene Ther. Methods.

[159] D’Costa S., Blouin V., Broucque F., Penaud-Budloo M., Francois A., Perez I.C., Le Bec C., Moullier P., Snyder R.O., Ayuso E. (2016). Practical utilization of recombinant AAV vector reference standards: Focus on vector genomes titration by free ITR qPCR. Mol. Ther. Methods Clin. Dev..

[160] Lock M., Alvira M.R., Chen S.J., Wilson J.M. (2014). Absolute determination of single-stranded and self-complementary adeno-associated viral vector genome titers by droplet digital PCR. Hum. Gene Ther. Methods.

[161] Strain M.C., Lada S.M., Luong T., Rought S.E., Gianella S., Terry V.H., Spina C.A., Woelk C.H., Richman D.D. (2013). Highly precise measurement of HIV DNA by droplet digital PCR. PLoS ONE.

[162] Clement N., Grieger J.C. (2016). Manufacturing of recombinant adeno-associated viral vectors for clinical trials. Mol. Ther. Methods Clin. Dev..

[163] Lai Y., Yue Y., Duan D. (2010). Evidence for the failure of adeno-associated virus serotype 5 to package a viral genome > or = 8.2 kb. Mol. Ther. J. Am. Soc. Gene Ther..

[164] Prusky G.T., Alam N.M., Beekman S., Douglas R.M. (2004). Rapid quantification of adult and developing mouse spatial vision using a virtual optomotor system. Investig. Ophthalmol. Vis. Sci..

[165] Clerin E., Wicker N., Mohand-Said S., Poch O., Sahel J.A., Leveillard T. (2011). e-conome: An automated tissue counting platform of cone photoreceptors for rodent models of retinitis pigmentosa. BMC Ophthalmol..

[166] Cronin T., Lyubarsky A., Bennett J. (2012). Dark-rearing the rd10 mouse: Implications for therapy. Adv. Exp. Med. Biol..

[167] Barone I., Novelli E., Piano I., Gargini C., Strettoi E. (2012). Environmental enrichment extends photoreceptor survival and visual function in a mouse model of retinitis pigmentosa. PLoS ONE.

[168] Li B., Gografe S., Munchow A., Lopez-Toledano M., Pan Z.H., Shen W. (2019). Sex-related differences in the progressive retinal degeneration of the rd10 mouse. Exp. Eye Res..

[169] Narayan D.S., Chidlow G., Wood J.P.M., Casson R.J. (2019). Investigations into Bioenergetic Neuroprotection of Cone Photoreceptors: Relevance to Retinitis Pigmentosa. Front. Neurosci..

[170] The International Council for Harmonisation of Technical Requirements for Pharmaceuticals for Human Use (2009). Guideline ICH. Guidance on nonclinical safety studies for the conduct of human clinical trials and marketing authorization for pharmaceuticals M3 (R2). International Conference on Harmonisation of Technical Requirements for Registration of Pharmaceuticals for Human Use.

[171] European Medicines Agency (2008). Guideline on the Non-Clinical Studies Required Before First Clinical Use of Gene Therapy Medicinal Products.

[172] The Food and Drug Administration (2018). Gene Therapy Guidance Document: Guidance for Industry: Preclinical Assessment of Investigational Cellular and Gene Therapy Products (2013).

[173] Weed L., Ammar M.J., Zhou S., Wei Z., Serrano L.W., Sun J., Lee V., Maguire A.M., Bennett J., Aleman T.S. (2019). Safety of Same-Eye Subretinal Sequential Readministration of AAV2-hRPE65v2 in Non-human Primates. Mol. Ther. Methods Clin. Dev..

[174] Ding K., Shen J., Hafiz Z., Hackett S.F., Silva R.L.E., Khan M., Lorenc V.E., Chen D., Chadha R., Zhang M. (2019). AAV8-vectored suprachoroidal gene transfer produces widespread ocular transgene expression. J. Clin. Investig..

[175] Bruewer A.R., Mowat F.M., Bartoe J.T., Boye S.L., Hauswirth W.W., Petersen-Jones S.M. (2013). Evaluation of lateral spread of transgene expression following subretinal AAV-mediated gene delivery in dogs. PLoS ONE.

[176] Singh H.P., Wang S., Stachelek K., Lee S., Reid M.W., Thornton M.E., Craft C.M., Grubbs B.H., Cobrinik D. (2018). Developmental stage-specific proliferation and retinoblastoma genesis in RB-deficient human but not mouse cone precursors. Proc. Natl. Acad. Sci. USA.

[177] Jauregui R., Park K.S., Duong J.K., Mahajan V.B., Tsang S.H. (2018). Quantitative progression of retinitis pigmentosa by optical coherence tomography angiography. Sci. Rep..

[178] Cabral T., Sengillo J.D., Duong J.K., Justus S., Boudreault K., Schuerch K., Belfort R., Mahajan V.B., Sparrow J.R., Tsang S.H. (2017). Retrospective Analysis of Structural Disease Progression in Retinitis Pigmentosa Utilizing Multimodal Imaging. Sci. Rep..

[179] Yusuf I.H., Birtel J., Shanks M.E., Clouston P., Downes S.M., Charbel Issa P., MacLaren R.E. (2019). Clinical Characterization of Retinitis Pigmentosa Associated With Variants in SNRNP200. JAMA Ophthalmol..

[180] Fujiwara K., Ikeda Y., Murakami Y., Tachibana T., Funatsu J., Koyanagi Y., Nakatake S., Yoshida N., Nakao S., Hisatomi T. (2018). Assessment of Central Visual Function in Patients with Retinitis Pigmentosa. Sci. Rep..

[181] Comander J., Weigel-DiFranco C., Sandberg M.A., Berson E.L. (2015). Visual Function in Carriers of X-Linked Retinitis Pigmentosa. Ophthalmology.

[182] Tee J.J.L., Yang Y., Kalitzeos A., Webster A., Bainbridge J., Michaelides M. (2019). Natural History Study of Retinal Structure, Progression, and Symmetry Using Ellipzoid Zone Metrics in RPGR-Associated Retinopathy. Am. J. Ophthalmol..

[183] Cideciyan A.V., Charng J., Roman A.J., Sheplock R., Garafalo A.V., Heon E., Jacobson S.G. (2018). Progression in X-linked Retinitis Pigmentosa Due to ORF15-RPGR Mutations: Assessment of Localized Vision Changes Over 2 Years. Investig. Ophthalmol. Vis. Sci..

[184] Chaumet-Riffaud A.E., Chaumet-Riffaud P., Cariou A., Devisme C., Audo I., Sahel J.A., Mohand-Said S. (2017). Impact of Retinitis Pigmentosa on Quality of Life, Mental Health, and Employment Among Young Adults. Am. J. Ophthalmol..

[185] Osterberg G. (1935). Topography of the Layer of Rods and Cones in the Human Retina.

[186] Staurenghi G., Sadda S., Chakravarthy U., Spaide R.F. (2014). International Nomenclature for Optical Coherence Tomography. Proposed lexicon for anatomic landmarks in normal posterior segment spectral-domain optical coherence tomography: The IN*OCT consensus. Ophthalmology.

[187] Smith T.B., Parker M.A., Steinkamp P.N., Romo A., Erker L.R., Lujan B.J., Smith N. (2019). Reliability of Spectral-Domain OCT Ellipsoid Zone Area and Shape Measurements in Retinitis Pigmentosa. Transl. Vis. Sci. Technol..

[188] Nowomiejska K., Brzozowska A., Koss M.J., Weleber R.G., Schiefer U., Rejdak K., Juenemann A.G., Maciejewski R., Rejdak R. (2016). Quantification of the Visual Field Loss in Retinitis Pigmentosa Using Semi-Automated Kinetic Perimetry. Curr. Eye Res..

[189] Schuerch K., Woods R.L., Lee W., Duncker T., Delori F.C., Allikmets R., Tsang S.H., Sparrow J.R. (2017). Quantifying Fundus Autofluorescence in Patients with Retinitis Pigmentosa. Investig. Ophthalmol. Vis. Sci..

[190] Sandberg M.A., Weigel-DiFranco C., Dryja T.P., Berson E.L. (1995). Clinical expression correlates with location of rhodopsin mutation in dominant retinitis pigmentosa. Investig. Ophthalmol. Vis. Sci..

[191] Gerkema M.P., Davies W.I., Foster R.G., Menaker M., Hut R.A. (2013). The nocturnal bottleneck and the evolution of activity patterns in mammals. Proc. Biol. Sci. R. Soc..

[192] Jaillard C., Mouret A., Niepon M.L., Clerin E., Yang Y., Lee-Rivera I., Ait-Ali N., Millet-Puel G., Cronin T., Sedmak T. (2012). Nxnl2 splicing results in dual functions in neuronal cell survival and maintenance of cell integrity. Hum. Mol. Genet..

