# Supplementary material for: Metabolic and Redox Signaling of the Nucleoredoxin-Like-1 Gene for the Treatment of Genetic Retinal Diseases
Source: Int J Mol Sci. 2020 Feb 27;21(5):1625. doi: 10.3390/ijms21051625 (PMC7084304; doi:10.3390/ijms21051625)
Supplement: Supplementary file 1 [file ijms-21-01625-s001.pdf]

November 8th 2019

Supplementary table I: The expression of genes mutated in retinitis pigmentosa, Bardet-Biedl syndrome, Leber congenital amaurosis and Usher syndrome.

N	Disease and Inheritance	Gene	*rd1* [1]	RD [2]	Mouse Tissue	RPE/ PR [3]	Freq.	Other Ret. Dis.	Other OMIM	Mouse Models	Other Models.
1	Autosomal dominant retinitis pigmentosa	*ARL3*	Rod-like	PR-death	Neurons	NF	Rare [4]	No	No	[5]	No
2		*ADIPOR1*	Rod-like	PR-death	Bone mar.	Ret./RPE	Rare [6]	One	No	[7]	No
3		*BEST1*	Hom	Infl.	Testis	NF	Rare [8]	Four	No	[9]	Dog [10, 11]
4		*CA4*	NE	NOp	Intestine	NF	Rare [12]	No	No	No	No
5		*CRX*	Rod-like	PR-death	Ret./RPE	Ret./RPE	1% [13]	Four	No	[14]	Cat [15]
6		*FSCN2*	Rod-like	NOp	Ret./RPE	Retina	3% [16]	One	No	[17]	No
7		*GUCA1B*	Rod-like	PR-death	Ret./RPE	Ret./RPE	0-5% [16]	One	No	[18]	No
8		*HK1*	Rod-like	Hom	Testis	Retina	Rare [19]	No	142600	No	No
9		*IMPDH1*	Rod-like	PR-death	Ret./RPE	Retina	2-3% [16]	One	No	[20]	No
10		*IMPG1*	Rod-like	PR-death	Ret./RPE	Retina	Rare [21]	Two	No	No	No
11		*KLHL7*	Hom	Hom	Ub	Ret./RPE	1-2% [22]	No	611119	No	No
12		*NR2E3*	Rod-like	PR-death	Ret./RPE	Retina	3.5% [23]	Four	No	[24]	No
13		*NRL*	Rod-like	PR-death	Ret./RPE	Retina	2% [16]	One	No	[25]	No

14		*PRPF3*	Hom	PR-death	Ub	Ret./RPE	1% [16]	No	No	[26]	No
15		*PRPF4*	Hom	Hom	Ub	RPE	Rare [27]	No	No	No	ZebF [28]
16		*PRPF6*	Hom	Hom	Ub	Ret./RPE	Rare [27]	No	No	No	No
17		*PRPF8*	Hom	PR-death	Ub	Ret./RPE	2% [16]	No	No	[26]	No
18		*PRPF31*	Rod-like	Hom	Ub	NF	2-4% [16]	No	No	[29]	No
19		*PRPH2*	Rod-like	PR-death	Ret./RPE	Ret./RPE	1-8% [16]	Six	No	[30, 31]	No
20		*RDH12*	Rod-like	PR-death	Ret./RPE	Ret./RPE	Rare [32]	One	No	[33]	No
21		*RHO*	Rod-like	PR-death	Ret./RPE	Ret./RPE	2-26%[16]	Two	No	[34-36]	[37, 38]
22		*ROM1*	Rod-like	PR-death	Ret./RPE	Ret./RPE	1% [16]	One	No	[39]	No
23		*RP1*	Rod-like	PR-death	Ret./RPE	RPE	4-8% [16]	No	No	[40] [41]	No
24		*RP9*	Cone-L.	PR-death	Ub	Retina	Rare [16]	No	No	No	No
25		*RPE65*	Hom	NE	RPE	NF	Rare	Two	No	[27]	Dog [42]
26		*SEMA4A*	Cone-L.	NOp	Ub	Ret./RPE	Rare [43]	One	No	[44]	No
27		*SNRNP200*	Hom	Hom	Ub	NF	<2% [45]	No	No	No	No
28		*SPP2*	NE	NOp	Kidn. Liv.	NF	Rare [46]	No	No	No	No
29		*TOPORS*	Hom	Hom	Ub	RPE	Rare [47]	No	No	[48]	No

30	**Autosomal recessive retinitis pigmentosa**	*ABCA4*	Rod-like	PR-death	Ret./RPE	Ret./RPE	5-6% [13]	Four	No	[49]	Dog [50]
31		*ADIPOR1*	Rod-like	PR-death	Bone Mar.	Ret./RPE	Rare	One	No	[7]	No
32		*AGBL5*	Hom	Hom	Testis	Retina	Rare [51]	No	No	No	No
33		*AHR*	Rod-like	Hom	Mast cells / Ret.	NF	Rare [52]	No	No	[53]	No
34		*ARHGEF18*	Hom	Hom	White cells / RPE	Ret./RPE	Rare [54]	No	No	No	No
35		*ARL6*	Rod-like	PR-death	Ub	Retina	1% [13]	One	No	[55]	No
36		*ARL2BP*	Hom	Hom	Testis	Ret./RPE	Rare [56]	No	No	No	No
37		*BBS1*	Rod-like	Hom	Ret./RPE	RPE	2-3% [13]	One	No	[57]	Zeb [58]
38		*BBS2*	Rod-like	Hom	Ub	Ret./RPE	0.8% [13]	One	No	[59]	No
39		*BEST1*	Hom	Infl.	Testis	NF	Rare [8]	Four	No	[9]	Dog [10, 11]
40		*C2orf71*	NF	PR-death	NF	NF	1% [60]	No	No	[61]	No
41		*C8ORF37*	No ortho	NOp	No ortho	No ortho	Rare [62]	Two	No	[63]	Zeb [64]
42		*CERKL*	Rod-like	NF	Ub	NF	1% [13]	One	No	[65]	No
43		*CLCC1*	Hom	Hom	Ub	Retina	Rare [66]	No	No	[66]	No
44		*CLRN1*	NOp	Infl.	NOp	NF	1% [13]	One	No	[67]	ZebF [68]
45		*CNGA1*	Rod-like	PR-death	Ret./RPE	Retina	1% [13]	No	No	No	Xen [69]

46	*CNGB1*	NF	PR-death	NF	Ret./RPE	4% [13]	No	No	*[70]*	Dog [71]
47	*CRB1*	Rod-like	Hom	Ret./RPE	NF	1% [13]	Three	No	[72]	Rat [73]
48	*CYP4V2*	Cone-L.	Hom	Liver	NF	Rare [74]	One	No	[75]	No
49	*DHDDS*	Hom	PR-death	Ub	Ret./RPE	1-8% [76]	No	No	No	ZebF [77]
50	*DHX38*	Hom	Hom	Ub	Retina	Rare [78]	One	No	No	No
51	*EMC1*	NF	Hom	NF	NF	Rare [79]	No	616846	No	No
52	*EYS*	No orth.	NOp	No ortho	No ortho	[80, 81]	No	No	No ortho	ZebF [82]
53	*FAM161A*	Rod-like	PR-death	Ret./RPE	NF	2% [83]	No	No	[84]	Dog [85]
54	*GPR125*	Hom	Hom	Epidermis	NF	Rare [79]	No	No	No	No
55	*HGSNAT*	Cone-L.	Hom	Microglia	Retina	Rare [86]	No	610453	No	No
56	*IDH3B*	Hom	Hom	Adipo.	Ret./RPE	Rare [87]	No	No	No	No
57	*IFT140*	Hom	Hom	Testis, Bone mar.	NF	Rare [88]	Two	614620	[89]	No
58	*IFT172*	Hom	PR-death	Testis	NF	Rare [90]	One	607386	[91]	No
59	*IMPG2*	NF	Hom	NF	RPE	Rare [92]	One	No	No	No
60	*KIAA1549*	No orth.	PR-death	No ortho	NF	Rare [79]	No	No	No	No
61	*KIZ*	Rod-like	NF	Testis	NF	Rare [93]	No	No	No	No

62	*LRAT*	Infl.	NOp	RPE	RPE	1% [13, 94]	One	No	[95]	No
63	*MAK*	Rod-like	PR-death	Ret./RPE	RPE	[76, 96]	No	No	[97]	No
64	*MERTK*	Cone-L.	Hom	Ub	RPE	1% [13]	No	No	[98]	[99]
65	*MVK*	Hom	Hom	Ub	Retina	Rare [100]	No	251170	[101]	No
66	*NEK2*	Cone-L.	NOp	Ub	NF	Rare [102]	No	No	[103]	ZebF [102]
67	*NEUROD1*	Rod-like	Hom	Cerebel.	NF	Rare [104]	No	601724	[105]	No
68	*NRL*	Rod-like	PR-death	Ret./RPE	Retina	2% [16]	One	No	[25]	No
69	*PDE6A*	Rod-like	PR-death	Ret./RPE	Retina	3-4% [13]	No	No	[106]	Dog [107]
70	*PDE6B*	Rod-like	PR-death	Ret./RPE	RPE	4-5% [13]	One	No	*rd1, rd10*	Dog [108]
71	*PDE6G*	Rod-like	PR-death	Ret./RPE	NF	Rare [109]	No	No	[110]	No
72	*POMGNT1*	Hom	Hom	Saliv. gl.	RPE	Rare [111]	No	606822	[112]	No
73	*PRCD*	No orth.	PR-death	No ortho	No ortho	Rare [113]	No	No	No ortho	Dog [114]
74	*PROM1*	Rod-like	PR-death	Ub	Ret./RPE	Rare [115]	Four	No	[113]	No
75	*RBP3*	Rod-like	PR-death	Ret./RPE	Ret./RPE	Rare [116]	No	No	[117]	No
76	*REEP6*	Rod-like	PR-death	Liver/Ret./RPE/Testis	RPE	Rare [118]	No	No	[119]	No
77	*RGR*	Infl.	PR-death	RPE	RPE	0.5% [13]	One	No	[120]	No

78		*RHO*	Rod-like	PR-death	Ret./RPE	Ret./RPE	1% [13]	Two	No	*Rho-/-*	No
79		*RLBP1*	Cone-L.	PR-death	Ret./RPE	Ret./RPE	1% [13]	Three	No	[121]	No
80		*RP1L1*	Rod-like	PR-death	Ret./RPE	NF	0.5% [122]	One	No	[123]	No
81		*RPE65*	Hom	NE	RPE	NF	2% [13]	Two	No	[27]	Dog [42]
82		*SAG*	Rod-like	PR-death	Ret./RPE	Ret./RPE	>1% [13]	One	No	[124]	Dog [125]
83		*SAMD11*	Rod-like	Hom	Bone mar. Ret./RPE	RPE	Rare [126]	No	No	No	No
84		*SLC7A14*	Cone-L.	PR-death	Brain	NF	Rare [127]	No	No	[127]	No
85		*SPATA7*	Hom	Hom	Testis	RPE	Rare [128]	One	No	[129]	No
86		*TRNT1*	Hom	NOp	Ub	NF	Rare [130]	No	612907	No	ZebF [131]
87		*TTC8*	Rod-like	PR-death	Ub	NF	>1% [13]	One	No	[132]	Dog [133]
88		*TULP1*	Rod-like	PR-death	Ret./RPE	Retina	1% [13]	One	No	[134]	No
89		*USH2A*	Rod-like	PR-death	Ub	Retina	17% [13]	One	No	[135]	ZebF [136]
90		*ZNF408*	No orth.	Infl.	No ortho	No ortho	Rare [137]	One	No	No	ZebF [138]
91		*ZNF513*	No orth.	Hom	No ortho	No ortho	Rare [139]	No	No	No	ZebF [139]
92	**X-linked retinitis pigmentosa**	*OFD1*	Hom	PR-death	Ub	RPE	Rare [140]	Two	300170	*[141]*	ZebF [142]
93		*RP2*	Hom	NOp	Ub	NF	7-10% [12]	No	No	[143]	No

94		*RPGR*	NF	PR-death	NF	NF	80% [12]	Three	No	[144]	Dog [145]
95	**Bardet-Biedl syndrome**	*ADIPOR1*	Rod-like	PR-death	Bone Mar.	Ret./RPE	Rare [6]	One	No	[7]	No
96		*ARL6*	Rod-like	PR-death	Ub	Retina	Rare [146]	One	No	[55]	No
97		*BBIP1*	NF	NF	NF	NF	Rare [146]	No	No	No	ZebF [147]
98		*BBS1*	Rod-like	Hom	Ret./RPE	RPE	23.2% [148]	One	No	[57]	ZebF [58]
99		*BBS2*	Rod-like	Hom	Ub	Ret./RPE	8.1% [148]	No	No	[59]	No
100		*BBS4*	Rod-like	PR-death	Brain	RPE	2.3% [146]	No	No	[149]	Dog [150]
101		*BBS5*	Rod-like	PR-death	Testis	NF	Rare [146]	No	No	[151]	No
102		*BBS7*	Rod-like	PR-death	Testis, Ret./RPE	RPE	1.5% [146]	No	No	[152]	NHP [153]
103		*BBS9*	Rod-like	PR-death	Ub	Ret.	6% [154]	No	No	No	ZebF [155]
104		*BBS10*	Hom	Hom	Mast cells	Ret./RPE	20% [156]	No	No	[157]	ZebF [156]
105		*BBS12*	Rod-like	PR-death	Mast cells, Ret./RPE	NF	5% [158]	No	No	[159]	No
106		*C8ORF37*	No ortho	NOp	No ortho	No ortho	Rare [160]	Two	No	[63]	ZebF [64]
107		*CEP19*	NF	NF	NF	NF	Rare [161]	No	No	[162]	No
108		*CEP290*	Rod-like	PR-death	Ret./RPE	NF	Rare [163]	Four	No	*rd16* [164] [165]	Cat [166]
109		*IFT172*	Hom	PR-death	Testis	NF	Rare [167]	One	No	[168]	ZebF [167] [169]

110		*IFT27*	NF	NF	NF	NF	Rare [170]	No	No	[171]	ZebF [170]
111		*INPP5E*	Hom	Hom	Testis	NF	Rare [172]	One	No	[172]	No
112		*KCNJ13*	Infl.	PR-death	RPE, Iris	RPE	Rare [173]	Two	No	[174]	ZebF [175]
113		*LZTFL1*	Rod-like	Hom	Testis, Bone mar.	NF	Rare [176]	No	No	[177]	No
114		*MKKS*	Rod-like	Hom	Muscle	NF	5.8% [146]	No	604896	[151, 178]	No
115		*MKS1*	Rod-like	Hom	Ub	Ret./RPE	4.5% [179]	One	609883	[180]	No
116		*NPHP1*	Rod-like	PR-death	Testis	RPE	Rare [176]	One	607100	[181]	No
117		*SDCCAG8*	Hom	Hom	Ub	Retina	Rare [176]	One	613524	[182]	ZebF [183]
118		*TRIM32*	Infl.	Infl.	Ub	RPE	Rare [176]	No	602290	[184]	No
119		*TTC8*	Rod-like	PR-death	Ub	NF	1.2% [148]	One	No	[132]	Dog [133]
120	**Autosomal dominant Leber congenital amaurosis**	*CRX*	Rod-like	PR-death	Ret./RPE	Ret./RPE	Rare [185]	Four	No	[14]	Cat [15]
121		*IMPDH1*	Rod-like	PR-death	Ret./RPE	Retina	Rare [186]	One	No	[20]	No
122		*OTX2*	Hom	PR-death	Ret./RPE	Ret./RPE	Rare [187]	One	No	[188]	No
123	**Autosomal recessive**	*AIPL1*	Rod-like	PR-death	Ret./RPE	Retina	4%-8% [189]	Two	No	[190]	No
124		*CABP4*	Rod-like	PR-death	Ret./RPE	Ret./RPE	Rare [191]	Two	No	[192]	No

125	**Leber congenital amaurosis**	*CCT2*	Hom	Hom	Ub	Ret./RPE	Rare [193]	No	No	No	ZebF [194]
126		*CEP290*	Rod-like	PR-death	Ret./RPE	NF	15%-20% [189]	Four	No	*rd16* [164] [165]	Cat [166]
127		*CLUAP1*	Hom	Hom	Testis	NF	Rare [195]	No	No	[196]	ZebF [197]
128		*CRB1*	Rod-like	Hom	Ret./RPE	NF	10% [189]	Three	No	[72]	Rat [73]
129		*CRX*	Rod-like	PR-death	Ret./RPE	Ret./RPE	Rare [185]	Four	No	[14]	Cat [15]
130		*DTHD1*	NF	NF	NF	NF	Rare [198]	No	No	No	No
131		*GDF6*	NF	NF	NF	NF	Rare [199]	Two	601147	[199]	ZebF [200]
132		*GUCY2D*	NF	PR-death	NF	NF	10%-20% [189]	No	No	[201]	Chick [202]
133		*IFT140*	Hom	Hom	Testis, Bone mar.	NF	Rare [203]	Two	614620	[89]	No
134		*IQCB1*	Hom	Hom	Ub	RPE	Rare [204]	No	609237	[205]	ZebF [206] Dog [207]
135		*KCNJ13*	Infl.	PR-death	RPE, Iris	RPE	Rare [208]	Two	No	[174]	ZebF [175]
136		*LCA5*	Rod-like	PR-death	Ret./RPE	RPE	Rare [209]	No	No	[210]	ZebF [211]
137		*LRAT*	Infl.	NOp	RPE	RPE	<1% [212]	One	No	[95]	No
138		*NMNAT1*	Hom	Hom	Ub	NF	Rare [213] [214] [215]	No	No	*Wld(S)* [216]	Droso [217]
139		*PRPH2*	Rod-like	PR-death	Ret./RPE	Ret./RPE	Rare [218]	Six	No	[30, 31]	No
140		*RD3*	Rod-like	Hom	Testis, Ret./RPE	Retina	Rare [212]	No	No	*rd3* [219]	No

141		*RDH12*	Rod-like	PR-death	Ret./RPE	Ret./RPE	4%-5% [189]	One	No	[33]	No
142		*RPE65*	Hom	NE	RPE	NF	5%–10% [189]	Two	No	[27]	Dog [42]
143		*RPGRIP1*	Rod-like	PR-death	Ub	Retina	5% [189]	One	No	[220]	Dog [221]
144		*SPATA7*	Hom	Hom	Testis	RPE	Rare [222]	One	No	[129]	No
145		*TULP1*	Rod-like	PR-death	Ret./RPE	Retina	Rare [223]	One	No	[134]	No
146	**Autosomal recessive Usher syndrome**	*ABHD12*	Infl.	Infl.	Ub	Ret./RPE	Rare [224]	No	No	[225]	No
147		*ADGRV1*	NF	NF	NF	NF	6.6%-19%* [226]	No	602851	[227] [228]	No
148		*ARSG*	Rod-like	NOp	RPE, Spinal c.	RPE	Rare [229]	No	No	[230]	Dog [231]
149		*CDH23*	NOp	Infl.	NOp	NF	7%-20%** [226]	No	605516	[232] [233]	Dog [234]
150		*CEP250*	Rod-like	Hom	Ub	Retina	Rare [235]	No	No	No	Cow [236]
151		*CEP78*	Hom	PR-death	Ub	Retina	Rare [237]	No	No	No	No
152		*CIB2*	Infl.	Infl.	Skel. m., Adipo.	Ret./RPE	Rare [235]	No	No	[238] [239]	ZebF [240]
153		*CLRN1*	NOp	Infl.	NOp	NF	Rare [235]	One	No	[67]	ZebF [68]
154		*DFNB31*	NF	PR-death	NF	RPE	0%-9.5%** [241]	No	No	*wi* [242]	No
155		*ESPN*	Infl.	Infl.	Intestine	Retina	Rare [243]	No	No	*je* [244]	No

156		*HARS*	Hom	Hom	Ub	RPE	Rare [235]	No	142810	No	No
157		*MYO7A*	Rod-like	Hom	Adrenal gland	RPE	53%-63%** [245]	No	No	*sh1* [246]	ZebF [247]
158		*PCDH15*	Rod-like	Hom	Retina	Retina	7%-12%** [245]	No	No	*av* [248]	Rat [249]
159		*USH1C*	Hom	Hom	Intestine	NF	1%-15%** [245]	No	No	*dfcr* [250] [251]	No
160		*USH1G*	NF	NOp	NF	NF	0%-4%** [245]	No	No	*js* [252]	No
		USH2A	Rod-like	PR-death	Ub	Retina	57%-79%* [226]	One	No	[135]	ZebF [136]

Legend: Adipo.: Adipocytes, Bone mar.: Bone Marrow, Cerebel.: Cerebellum, Cone-L.: Cone-like: increase in the *rd1* retina (after rod degeneration) and decrease in human retinal detachment (after death of both rods an cones), Droso: Drosophila, Freq: Frequency from Retnet (https://sph.uth.edu/Retnet/) and Pubmed, Hom: Homogenous, Infl.: Inflammatory cells, Kidn.: Kidney, Liv.: Liver, NE: Not expressed, NF: Not found, NHP: non-human primate, NOp: Probeset non-operational, No ortho: No orthologue, PR-death: Decrease in human retinal detachment, Ret.: retina, Rod-like: decrease in the *rd1* retina. Rod-like expression pattern corresponds to an expression profile that march that of the rhodopsin gene (*Rho*) that decrease tin the *rd1* retina during the course of rod degeneration., RPE: retinal pigmented epithelium, Saliv. gl.: Salivary gland, Skel. m.: skeleton muscle, Spinal c.: Spinal cord Ub: Ubiquitous, Xen: Xenopus and ZebF: Zebrafish, *: Percentage of Usher syndrome type II, **: Percentage of Usher syndrome type I.

References:

1. Ait-Ali, N.; Fridlich, R.; Millet-Puel, G.; Clerin, E.; Delalande, F.; Jaillard, C.; Blond, F.; Perrocheau, L.; Reichman, S.; Byrne, L. C.; Olivier-Bandini, A.; Bellalou, J.; Moyse, E.; Bouillaud, F.; Nicol, X.; Dalkara, D.; van Dorsselaer, A.; Sahel, J. A.; Leveillard, T., Rod-derived cone viability factor promotes cone survival by stimulating aerobic glycolysis. *Cell* **2015,** 161, (4), 817-32.
2. Delyfer, M. N.; Raffelsberger, W.; Mercier, D.; Korobelnik, J. F.; Gaudric, A.; Charteris, D. G.; Tadayoni, R.; Metge, F.; Caputo, G.; Barale, P. O.; Ripp, R.; Muller, J. D.; Poch, O.; Sahel, J. A.; Leveillard, T., Transcriptomic analysis of human retinal detachment reveals both inflammatory response and photoreceptor death. *PloS one* **2011,** 6, (12), e28791.

3. Kole, C.; Berdugo, N.; Da Silva, C.; Ait-Ali, N.; Millet-Puel, G.; Pagan, D.; Blond, F.; Poidevin, L.; Ripp, R.; Fontaine, V.; Wincker, P.; Zack, D. J.; Sahel, J. A.; Poch, O.; Leveillard, T., Identification of an Alternative Splicing Product of the Otx2 Gene Expressed in the Neural Retina and Retinal Pigmented Epithelial Cells. *PloS one* **2016,** 11, (3), e0150758.
4. Strom, S. P.; Clark, M. J.; Martinez, A.; Garcia, S.; Abelazeem, A. A.; Matynia, A.; Parikh, S.; Sullivan, L. S.; Bowne, S. J.; Daiger, S. P.; Gorin, M. B., De Novo Occurrence of a Variant in ARL3 and Apparent Autosomal Dominant Transmission of Retinitis Pigmentosa. *PloS one* **2016,** 11, (3), e0150944.
5. Wright, Z. C.; Singh, R. K.; Alpino, R.; Goldberg, A. F.; Sokolov, M.; Ramamurthy, V., ARL3 regulates trafficking of prenylated phototransduction proteins to the rod outer segment. *Human molecular genetics* **2016**.
6. Xu, M.; Eblimit, A.; Wang, J.; Li, J.; Wang, F.; Zhao, L.; Wang, X.; Xiao, N.; Li, Y.; Wong, L. J.; Lewis, R. A.; Chen, R., ADIPOR1 Is Mutated in Syndromic Retinitis Pigmentosa. *Hum Mutat* **2016,** 37, (3), 246-9.
7. Sluch, V. M.; Banks, A.; Li, H.; Crowley, M. A.; Davis, V.; Xiang, C.; Yang, J.; Demirs, J. T.; Vrouvlianis, J.; Leehy, B.; Hanks, S.; Hyman, A. M.; Aranda, J.; Chang, B.; Bigelow, C. E.; Rice, D. S., ADIPOR1 is essential for vision and its RPE expression is lost in the Mfrp(rd6) mouse. *Sci Rep* **2018,** 8, (1), 14339.
8. Davidson, A. E.; Millar, I. D.; Urquhart, J. E.; Burgess-Mullan, R.; Shweikh, Y.; Parry, N.; O'Sullivan, J.; Maher, G. J.; McKibbin, M.; Downes, S. M.; Lotery, A. J.; Jacobson, S. G.; Brown, P. D.; Black, G. C.; Manson, F. D., Missense mutations in a retinal pigment epithelium protein, bestrophin-1, cause retinitis pigmentosa. *American journal of human genetics* **2009,** 85, (5), 581-92.
9. Zhang, Y.; Stanton, J. B.; Wu, J.; Yu, K.; Hartzell, H. C.; Peachey, N. S.; Marmorstein, L. Y.; Marmorstein, A. D., Suppression of Ca2+ signaling in a mouse model of Best disease. *Human molecular genetics* **2010,** 19, (6), 1108-18.
10. Guziewicz, K. E.; Zangerl, B.; Lindauer, S. J.; Mullins, R. F.; Sandmeyer, L. S.; Grahn, B. H.; Stone, E. M.; Acland, G. M.; Aguirre, G. D., Bestrophin gene mutations cause canine multifocal retinopathy: a novel animal model for best disease. *Investigative ophthalmology & visual science* **2007,** 48, (5), 1959-67.
11. Zangerl, B.; Wickstrom, K.; Slavik, J.; Lindauer, S. J.; Ahonen, S.; Schelling, C.; Lohi, H.; Guziewicz, K. E.; Aguirre, G. D., Assessment of canine BEST1 variations identifies new mutations and establishes an independent bestrophinopathy model (cmr3). *Molecular vision* **2010,** 16, 2791-804.
12. Datta, R.; Waheed, A.; Bonapace, G.; Shah, G. N.; Sly, W. S., Pathogenesis of retinitis pigmentosa associated with apoptosis-inducing mutations in carbonic anhydrase IV. *Proceedings of the National Academy of Sciences of the United States of America* **2009,** 106, (9), 3437-42.
13. Hartong, D. T.; Berson, E. L.; Dryja, T. P., Retinitis pigmentosa. *Lancet* **2006,** 368, (9549), 1795-809.
14. Tran, N. M.; Zhang, A.; Zhang, X.; Huecker, J. B.; Hennig, A. K.; Chen, S., Mechanistically distinct mouse models for CRX-associated retinopathy. *PLoS genetics* **2014,** 10, (2), e1004111.
15. Occelli, L. M.; Tran, N. M.; Narfstrom, K.; Chen, S.; Petersen-Jones, S. M., CrxRdy Cat: A Large Animal Model for CRX-Associated Leber Congenital Amaurosis. *Investigative ophthalmology & visual science* **2016,** 57, (8), 3780-92.

16. Sullivan, L. S.; Bowne, S. J.; Birch, D. G.; Hughbanks-Wheaton, D.; Heckenlively, J. R.; Lewis, R. A.; Garcia, C. A.; Ruiz, R. S.; Blanton, S. H.; Northrup, H.; Gire, A. I.; Seaman, R.; Duzkale, H.; Spellicy, C. J.; Zhu, J.; Shankar, S. P.; Daiger, S. P., Prevalence of disease-causing mutations in families with autosomal dominant retinitis pigmentosa: a screen of known genes in 200 families. *Investigative ophthalmology & visual science* **2006,** 47, (7), 3052-64.

17. Yokokura, S.; Wada, Y.; Nakai, S.; Sato, H.; Yao, R.; Yamanaka, H.; Ito, S.; Sagara, Y.; Takahashi, M.; Nakamura, Y.; Tamai, M.; Noda, T., Targeted disruption of FSCN2 gene induces retinopathy in mice. *Investigative ophthalmology & visual science* **2005,** 46, (8), 2905-15.

18. Pennesi, M. E.; Howes, K. A.; Baehr, W.; Wu, S. M., Guanylate cyclase-activating protein (GCAP) 1 rescues cone recovery kinetics in GCAP1/GCAP2 knockout mice. *Proceedings of the National Academy of Sciences of the United States of America* **2003,** 100, (11), 6783-8.

19. Sullivan, L. S.; Koboldt, D. C.; Bowne, S. J.; Lang, S.; Blanton, S. H.; Cadena, E.; Avery, C. E.; Lewis, R. A.; Webb-Jones, K.; Wheaton, D. H.; Birch, D. G.; Coussa, R.; Ren, H. A.; Lopez, I.; Chakarova, C.; Koenekoop, R. K.; Garcia, C. A.; Fulton, R. S.; Wilson, R. K.; Weinstock, G. M.; Daiger, S. P., A Dominant Mutation in Hexokinase 1 (HK1) Causes Retinitis Pigmentosa. *Investigative ophthalmology & visual science* **2014,** 55, (11), 7147-7158.

20. Tam, L. C.; Kiang, A. S.; Kennan, A.; Kenna, P. F.; Chadderton, N.; Ader, M.; Palfi, A.; Aherne, A.; Ayuso, C.; Campbell, M.; Reynolds, A.; McKee, A.; Humphries, M. M.; Farrar, G. J.; Humphries, P., Therapeutic benefit derived from RNAi-mediated ablation of IMPDH1 transcripts in a murine model of autosomal dominant retinitis pigmentosa (RP10). *Human molecular genetics* **2008,** 17, (14), 2084-100.

21. Manes, G.; Meunier, I.; Avila-Fernandez, A.; Banfi, S.; Le Meur, G.; Zanlonghi, X.; Corton, M.; Simonelli, F.; Brabet, P.; Labesse, G.; Audo, I.; Mohand-Said, S.; Zeitz, C.; Sahel, J. A.; Weber, M.; Dollfus, H.; Dhaenens, C. M.; Allorge, D.; De Baere, E.; Koenekoop, R. K.; Kohl, S.; Cremers, F. P.; Hollyfield, J. G.; Senechal, A.; Hebrard, M.; Bocquet, B.; Ayuso Garcia, C.; Hamel, C. P., Mutations in IMPG1 cause vitelliform macular dystrophies. *Am J Hum Genet* **2013,** 93, (3), 571-8.

22. Friedman, J. S.; Ray, J. W.; Waseem, N.; Johnson, K.; Brooks, M. J.; Hugosson, T.; Breuer, D.; Branham, K. E.; Krauth, D. S.; Bowne, S. J.; Sullivan, L. S.; Ponjavic, V.; Granse, L.; Khanna, R.; Trager, E. H.; Gieser, L. M.; Hughbanks-Wheaton, D.; Cojocaru, R. I.; Ghiasvand, N. M.; Chakarova, C. F.; Abrahamson, M.; Goring, H. H.; Webster, A. R.; Birch, D. G.; Abecasis, G. R.; Fann, Y.; Bhattacharya, S. S.; Daiger, S. P.; Heckenlively, J. R.; Andreasson, S.; Swaroop, A., Mutations in a BTB-Kelch protein, KLHL7, cause autosomal-dominant retinitis pigmentosa. *American journal of human genetics* **2009,** 84, (6), 792-800.

23. Blanco-Kelly, F.; Garcia Hoyos, M.; Lopez Martinez, M. A.; Lopez-Molina, M. I.; Riveiro-Alvarez, R.; Fernandez-San Jose, P.; Avila-Fernandez, A.; Corton, M.; Millan, J. M.; Garcia Sandoval, B.; Ayuso, C., Dominant Retinitis Pigmentosa, p.Gly56Arg Mutation in NR2E3: Phenotype in a Large Cohort of 24 Cases. *PloS one* **2016,** 11, (2), e0149473.

24. Akhmedov, N. B.; Piriev, N. I.; Chang, B.; Rapoport, A. L.; Hawes, N. L.; Nishina, P. M.; Nusinowitz, S.; Heckenlively, J. R.; Roderick, T. H.; Kozak, C. A.; Danciger, M.; Davisson, M. T.; Farber, D. B., A deletion in a photoreceptor-specific nuclear receptor mRNA causes retinal degeneration in the rd7 mouse. *Proceedings of the National Academy of Sciences of the United States of America* **2000,** 97, (10), 5551-6.

25. Mears, A. J.; Kondo, M.; Swain, P. K.; Takada, Y.; Bush, R. A.; Saunders, T. L.; Sieving, P. A.; Swaroop, A., Nrl is required for rod photoreceptor development. *Nat Genet* **2001,** 29, (4), 447-52.

26. Graziotto, J. J.; Farkas, M. H.; Bujakowska, K.; Deramaudt, B. M.; Zhang, Q.; Nandrot, E. F.; Inglehearn, C. F.; Bhattacharya, S. S.; Pierce, E. A., Three Gene-Targeted Mouse Models of RNA Splicing Factor RP Show Late-Onset RPE and Retinal Degeneration. *Investigative ophthalmology & visual science* **2011,** 52, (1), 190-198.
27. Ruzickova, S.; Stanek, D., Mutations in spliceosomal proteins and retina degeneration. *RNA biology* **2016**, 1-9.
28. Chen, X.; Liu, Y.; Sheng, X.; Tam, P. O.; Zhao, K.; Chen, X.; Rong, W.; Liu, Y.; Liu, X.; Pan, X.; Chen, L. J.; Zhao, Q.; Vollrath, D.; Pang, C. P.; Zhao, C., PRPF4 mutations cause autosomal dominant retinitis pigmentosa. *Human molecular genetics* **2014,** 23, (11), 2926-39.
29. Farkas, M. H.; Lew, D. S.; Sousa, M. E.; Bujakowska, K.; Chatagnon, J.; Bhattacharya, S. S.; Pierce, E. A.; Nandrot, E. F., Mutations in pre-mRNA processing factors 3, 8, and 31 cause dysfunction of the retinal pigment epithelium. *The American journal of pathology* **2014,** 184, (10), 2641-52.
30. Kajiwara, K.; Hahn, L. B.; Mukai, S.; Travis, G. H.; Berson, E. L.; Dryja, T. P., Mutations in the human retinal degeneration slow gene in autosomal dominant retinitis pigmentosa. *Nature* **1991,** 354, (6353), 480-3.
31. Hawes, N. L.; Smith, R. S.; Chang, B.; Davisson, M.; Heckenlively, J. R.; John, S. W., Mouse fundus photography and angiography: a catalogue of normal and mutant phenotypes. *Molecular vision* **1999,** 5, 22.
32. Fingert, J. H.; Oh, K.; Chung, M.; Scheetz, T. E.; Andorf, J. L.; Johnson, R. M.; Sheffield, V. C.; Stone, E. M., Association of a novel mutation in the retinol dehydrogenase 12 (RDH12) gene with autosomal dominant retinitis pigmentosa. *Archives of ophthalmology* **2008,** 126, (9), 1301-7.
33. Maeda, A.; Maeda, T.; Sun, W.; Zhang, H.; Baehr, W.; Palczewski, K., Redundant and unique roles of retinol dehydrogenases in the mouse retina. *Proceedings of the National Academy of Sciences of the United States of America* **2007,** 104, (49), 19565-70.
34. Olsson, J. E.; Gordon, J. W.; Pawlyk, B. S.; Roof, D.; Hayes, A.; Molday, R. S.; Mukai, S.; Cowley, G. S.; Berson, E. L.; Dryja, T. P., Transgenic mice with a rhodopsin mutation (Pro23His): a mouse model of autosomal dominant retinitis pigmentosa. *Neuron* **1992,** 9, (5), 815-30.
35. Kennan, A.; Aherne, A.; Palfi, A.; Humphries, M.; McKee, A.; Stitt, A.; Simpson, D. A.; Demtroder, K.; Orntoft, T.; Ayuso, C.; Kenna, P. F.; Farrar, G. J.; Humphries, P., Identification of an IMPDH1 mutation in autosomal dominant retinitis pigmentosa (RP10) revealed following comparative microarray analysis of transcripts derived from retinas of wild-type and Rho(-/-) mice. *Hum Mol Genet* **2002,** 11, (5), 547-57.
36. Orhan, E.; Dalkara, D.; Neuille, M.; Lechauve, C.; Michiels, C.; Picaud, S.; Leveillard, T.; Sahel, J. A.; Naash, M. I.; Lavail, M. M.; Zeitz, C.; Audo, I., Genotypic and phenotypic characterization of P23H line 1 rat model. *PLoS One* **2015,** 10, (5), e0127319.
37. Wang, W.; Lee, S. J.; Scott, P. A.; Lu, X.; Emery, D.; Liu, Y.; Ezashi, T.; Roberts, M. R.; Ross, J. W.; Kaplan, H. J.; Dean, D. C., Two-Step Reactivation of Dormant Cones in Retinitis Pigmentosa. *Cell Rep* **2016,** 15, (2), 372-85.
38. Kostic, C.; Arsenijevic, Y., Animal modelling for inherited central vision loss. *The Journal of pathology* **2016,** 238, (2), 300-10.
39. Clarke, G.; Goldberg, A. F. X.; Vidgen, D.; Collins, L.; Ploder, L.; Schwarz, L.; Molday, L. L.; Rossant, J.; Szel, A.; Molday, R. S.; Birch, D. G.; McInnes, R. R., Rom-1 is required for rod photoreceptor viability and the regulation of disk morphogenesis. *Nature genetics* **2000,** 25, (1), 67-73.

40. Liu, Q.; Zuo, J.; Pierce, E. A., The retinitis pigmentosa 1 protein is a photoreceptor microtubule- associated protein. *Journal of Neuroscience* **2004,** 24, (29), 6427-6436.
41. Song, D.; Grieco, S.; Li, Y.; Hunter, A.; Chu, S.; Zhao, L.; Song, Y.; DeAngelis, R. A.; Shi, L. Y.; Liu, Q.; Pierce, E. A.; Nishina, P. M.; Lambris, J. D.; Dunaief, J. L., A murine RP1 missense mutation causes protein mislocalization and slowly progressive photoreceptor degeneration. *The American journal of pathology* **2014,** 184, (10), 2721-9.
42. Acland, G. M.; Aguirre, G. D.; Ray, J.; Zhang, Q.; Aleman, T. S.; Cideciyan, A. V.; Pearce-Kelling, S. E.; Anand, V.; Zeng, Y.; Maguire, A. M.; Jacobson, S. G.; Hauswirth, W. W.; Bennett, J., Gene therapy restores vision in a canine model of childhood blindness. *Nat Genet* **2001,** 28, (1), 92-5.
43. Abid, A.; Ismail, M.; Mehdi, S. Q.; Khaliq, S., Identification of novel mutations in the SEMA4A gene associated with retinal degenerative diseases. *Journal of medical genetics* **2006,** 43, (4), 378-81.
44. Rice, D. S.; Huang, W.; Jones, H. A.; Hansen, G.; Ye, G. L.; Xu, N.; Wilson, E. A.; Troughton, K.; Vaddi, K.; Newton, R. C.; Zambrowicz, B. P.; Sands, A. T., Severe retinal degeneration associated with disruption of semaphorin 4A. *Investigative ophthalmology & visual science* **2004,** 45, (8), 2767-77.
45. Coussa, R. G.; Chakarova, C.; Ajlan, R.; Taha, M.; Kavalec, C.; Gomolin, J.; Khan, A.; Lopez, I.; Ren, H.; Waseem, N.; Kamenarova, K.; Bhattacharya, S. S.; Koenekoop, R. K., Genotype and Phenotype Studies in Autosomal Dominant Retinitis Pigmentosa (adRP) of the French Canadian Founder Population. *Investigative ophthalmology & visual science* **2015,** 56, (13), 8297-305.
46. Liu, Y.; Chen, X.; Xu, Q.; Gao, X.; Tam, P. O.; Zhao, K.; Zhang, X.; Chen, L. J.; Jia, W.; Zhao, Q.; Vollrath, D.; Pang, C. P.; Zhao, C., SPP2 Mutations Cause Autosomal Dominant Retinitis Pigmentosa. *Scientific reports* **2015,** 5, 14867.
47. Schob, C.; Orth, U.; Gal, A.; Kindler, S.; Chakarova, C. F.; Bhattacharya, S. S.; Ruther, K., Mutations in TOPORS: a rare cause of autosomal dominant retinitis pigmentosa in continental Europe? *Ophthalmic genetics* **2009,** 30, (2), 96-8.
48. Marshall, H.; Bhaumik, M.; Aviv, H.; Moore, D.; Yao, M.; Dutta, J.; Rahim, H.; Gounder, M.; Ganesan, S.; Saleem, A.; Rubin, E., Deficiency of the dual ubiquitin/SUMO ligase Topors results in genetic instability and an increased rate of malignancy in mice. *BMC molecular biology* **2010,** 11, 31.
49. Weng, J.; Mata, N. L.; Azarian, S. M.; Tzekov, R. T.; Birch, D. G.; Travis, G. H., Insights into the function of Rim protein in photoreceptors and etiology of Stargardt's disease from the phenotype in abcr knockout mice. *Cell* **1999,** 98, (1), 13-23.
50. Makelainen, S.; Godia, M.; Hellsand, M.; Viluma, A.; Hahn, D.; Makdoumi, K.; Zeiss, C. J.; Mellersh, C.; Ricketts, S. L.; Narfstrom, K.; Hallbook, F.; Ekesten, B.; Andersson, G.; Bergstrom, T. F., An ABCA4 loss-of-function mutation causes a canine form of Stargardt disease. *PLoS Genet* **2019,** 15, (3), e1007873.
51. Branham, K.; Matsui, H.; Biswas, P.; Guru, A. A.; Hicks, M.; Suk, J. J.; Li, H.; Jakubosky, D.; Long, T.; Telenti, A.; Nariai, N.; Heckenlively, J. R.; Frazer, K. A.; Sieving, P. A.; Ayyagari, R., Establishing the involvement of the novel gene AGBL5 in retinitis pigmentosa by whole genome sequencing. *Physiol Genomics* **2016,** 48, (12), 922-927.
52. Zhou, Y.; Li, S.; Huang, L.; Yang, Y.; Zhang, L.; Yang, M.; Liu, W.; Ramasamy, K.; Jiang, Z.; Sundaresan, P.; Zhu, X.; Yang, Z., A splicing mutation in aryl hydrocarbon receptor associated with retinitis pigmentosa. *Hum Mol Genet* **2018,** 27, (14), 2563-2572.

53. Kim, S. Y.; Yang, H. J.; Chang, Y. S.; Kim, J. W.; Brooks, M.; Chew, E. Y.; Wong, W. T.; Fariss, R. N.; Rachel, R. A.; Cogliati, T.; Qian, H.; Swaroop, A., Deletion of aryl hydrocarbon receptor AHR in mice leads to subretinal accumulation of microglia and RPE atrophy. *Invest Ophthalmol Vis Sci* **2014,** 55, (9), 6031-40.

54. Arno, G.; Carss, K. J.; Hull, S.; Zihni, C.; Robson, A. G.; Fiorentino, A.; Consortium, U. K. I. R. D.; Hardcastle, A. J.; Holder, G. E.; Cheetham, M. E.; Plagnol, V.; Consortium, N. B.-R. D.; Moore, A. T.; Raymond, F. L.; Matter, K.; Balda, M. S.; Webster, A. R., Biallelic Mutation of ARHGEF18, Involved in the Determination of Epithelial Apicobasal Polarity, Causes Adult-Onset Retinal Degeneration. *Am J Hum Genet* **2017,** 100, (2), 334-342.

55. Zhang, Q.; Nishimura, D.; Seo, S.; Vogel, T.; Morgan, D. A.; Searby, C.; Bugge, K.; Stone, E. M.; Rahmouni, K.; Sheffield, V. C., Bardet-Biedl syndrome 3 (Bbs3) knockout mouse model reveals common BBS-associated phenotypes and Bbs3 unique phenotypes. *Proceedings of the National Academy of Sciences of the United States of America* **2011,** 108, (51), 20678-83.

56. Davidson, A. E.; Schwarz, N.; Zelinger, L.; Stern-Schneider, G.; Shoemark, A.; Spitzbarth, B.; Gross, M.; Laxer, U.; Sosna, J.; Sergouniotis, P. I.; Waseem, N. H.; Wilson, R.; Kahn, R. A.; Plagnol, V.; Wolfrum, U.; Banin, E.; Hardcastle, A. J.; Cheetham, M. E.; Sharon, D.; Webster, A. R., Mutations in ARL2BP, encoding ADP-ribosylation-factor-like 2 binding protein, cause autosomal-recessive retinitis pigmentosa. *American journal of human genetics* **2013,** 93, (2), 321-9.

57. Davis, R. E.; Swiderski, R. E.; Rahmouni, K.; Nishimura, D. Y.; Mullins, R. F.; Agassandian, K.; Philp, A. R.; Searby, C. C.; Andrews, M. P.; Thompson, S.; Berry, C. J.; Thedens, D. R.; Yang, B.; Weiss, R. M.; Cassell, M. D.; Stone, E. M.; Sheffield, V. C., A knockin mouse model of the Bardet-Biedl syndrome 1 M390R mutation has cilia defects, ventriculomegaly, retinopathy, and obesity. *Proceedings of the National Academy of Sciences of the United States of America* **2007,** 104, (49), 19422-19427.

58. Castro-Sanchez, S.; Suarez-Bregua, P.; Novas, R.; Alvarez-Satta, M.; Badano, J. L.; Rotllant, J.; Valverde, D., Functional analysis of new human Bardet-Biedl syndrome loci specific variants in the zebrafish model. *Sci Rep* **2019,** 9, (1), 12936.

59. Nishimura, D. Y.; Fath, M.; Mullins, R. F.; Searby, C.; Andrews, M.; Davis, R.; Andorf, J. L.; Mykytyn, K.; Swiderski, R. E.; Yang, B. L.; Carmi, R.; Stone, E. M.; Sheffield, V. C., Bbs2-null mice have neurosensory deficits, a defect in social dominance, and retinopathy associated with mislocalization of rhodopsin. *Proceedings of the National Academy of Sciences of the United States of America* **2004,** 101, (47), 16588-16593.

60. Audo, I.; Lancelot, M. E.; Mohand-Said, S.; Antonio, A.; Germain, A.; Sahel, J. A.; Bhattacharya, S. S.; Zeitz, C., Novel C2orf71 Mutations Account for similar to 1% of Cases in a Large French arRP Cohort. *Human mutation* **2011,** 32, (4), E2091-E2103.

61. Kevany, B. M.; Zhang, N.; Jastrzebska, B.; Palczewski, K., Animals deficient in C2Orf71, an autosomal recessive retinitis pigmentosa-associated locus, develop severe early-onset retinal degeneration. *Human molecular genetics* **2015,** 24, (9), 2627-2640.

62. Ravesh, Z.; El Asrag, M. E.; Weisschuh, N.; McKibbin, M.; Reuter, P.; Watson, C. M.; Baumann, B.; Poulter, J. A.; Sajid, S.; Panagiotou, E. S.; O'Sullivan, J.; Abdelhamed, Z.; Bonin, M.; Soltanifar, M.; Black, G. C. M.; Din, M. A.; Toomes, C.; Ansar, M.; Inglehearn, C. F.; Wissinger, B.; Ali, M., Novel C8orf37 mutations cause retinitis pigmentosa in consanguineous families of Pakistani origin. *Molecular vision* **2015,** 21, 236-243.

63. Sharif, A. S.; Yu, D.; Loertscher, S.; Austin, R.; Nguyen, K.; Mathur, P. D.; Clark, A. M.; Zou, J.; Lobanova, E. S.; Arshavsky, V. Y.; Yang, J., C8ORF37 Is Required for Photoreceptor Outer Segment Disc Morphogenesis by Maintaining Outer Segment Membrane Protein Homeostasis. *J Neurosci* **2018,** 38, (13), 3160-3176.
64. Heon, E.; Kim, G.; Qin, S.; Garrison, J. E.; Tavares, E.; Vincent, A.; Nuangchamnong, N.; Scott, C. A.; Slusarski, D. C.; Sheffield, V. C., Mutations in C8ORF37 cause Bardet Biedl syndrome (BBS21). *Hum Mol Genet* **2016,** 25, (11), 2283-2294.
65. Garanto, A.; Mandal, N. A.; Egido-Gabas, M.; Marfany, G.; Fabrias, G.; Anderson, R. E.; Casas, J.; Gonzalez-Duarte, R., Specific sphingolipid content decrease in Cerkl knockdown mouse retinas. *Experimental eye research* **2013,** 110, 96-106.
66. Li, L.; Jiao, X.; D'Atri, I.; Ono, F.; Nelson, R.; Chan, C. C.; Nakaya, N.; Ma, Z.; Ma, Y.; Cai, X.; Zhang, L.; Lin, S.; Hameed, A.; Chioza, B. A.; Hardy, H.; Arno, G.; Hull, S.; Khan, M. I.; Fasham, J.; Harlalka, G. V.; Michaelides, M.; Moore, A. T.; Coban Akdemir, Z. H.; Jhangiani, S.; Lupski, J. R.; Cremers, F. P. M.; Qamar, R.; Salman, A.; Chilton, J.; Self, J.; Ayyagari, R.; Kabir, F.; Naeem, M. A.; Ali, M.; Akram, J.; Sieving, P. A.; Riazuddin, S.; Baple, E. L.; Riazuddin, S. A.; Crosby, A. H.; Hejtmancik, J. F., Mutation in the intracellular chloride channel CLCC1 associated with autosomal recessive retinitis pigmentosa. *PLoS Genet* **2018,** 14, (8), e1007504.
67. Tian, G.; Lee, R.; Ropelewski, P.; Imanishi, Y., Impairment of Vision in a Mouse Model of Usher Syndrome Type III. *Investigative ophthalmology & visual science* **2016,** 57, (3), 866-875.
68. Gopal, S. R.; Chen, D. H. C.; Chou, S. W.; Zang, J. J.; Neuhauss, S. C. F.; Stepanyan, R.; McDermott, B. M.; Alagramam, K. N., Zebrafish Models for the Mechanosensory Hair Cell Dysfunction in Usher Syndrome 3 Reveal That Clarin-1 Is an Essential Hair Bundle Protein. *Journal of Neuroscience* **2015,** 35, (28), 10188-10201.
69. Trudeau, M. C.; Zagotta, W. N., An intersubunit interaction regulates trafficking of rod cyclic nucleotide-gated channels and is disrupted in an inherited form of blindness. *Neuron* **2002,** 34, (2), 197-207.
70. Huttl, S.; Michalakis, S.; Seeliger, M.; Luo, D. G.; Acar, N.; Geiger, H.; Hudl, K.; Mader, R.; Haverkamp, S.; Moser, M.; Pfeifer, A.; Gerstner, A.; Yau, K. W.; Biel, M., Impaired channel targeting and retinal degeneration in mice lacking the cyclic nucleotide-gated channel subunit CNGB1. *Journal of Neuroscience* **2005,** 25, (1), 130-138.
71. Winkler, P. A.; Ekenstedt, K. J.; Occelli, L. M.; Frattaroli, A. V.; Bartoe, J. T.; Venta, P. J.; Petersen-Jones, S. M., A Large Animal Model for CNGB1 Autosomal Recessive Retinitis Pigmentosa. *PloS one* **2013,** 8, (8).
72. Mehalow, A. K.; Kameya, S.; Smith, R. S.; Hawes, N. L.; Denegre, J. M.; Young, J. A.; Bechtold, L.; Haider, N. B.; Tepass, U.; Heckenlively, J. R.; Chang, B.; Naggert, J. K.; Nishina, P. M., CRB1 is essential for external limiting membrane integrity and photoreceptor morphogenesis in the mammalian retina. *Human molecular genetics* **2003,** 12, (17), 2179-2189.
73. Zhao, M.; Andrieu-Soler, C.; Kowalczuk, L.; Paz Cortes, M.; Berdugo, M.; Dernigoghossian, M.; Halili, F.; Jeanny, J. C.; Goldenberg, B.; Savoldelli, M.; El Sanharawi, M.; Naud, M. C.; van Ijcken, W.; Pescini-Gobert, R.; Martinet, D.; Maass, A.; Wijnholds, J.; Crisanti, P.; Rivolta, C.; Behar-Cohen, F., A new CRB1 rat mutation links Muller glial cells to retinal telangiectasia. *The Journal of neuroscience : the official journal of the Society for Neuroscience* **2015,** 35, (15), 6093-106.

74. Wang, Y.; Guo, L.; Cai, S. P.; Dai, M.; Yang, Q.; Yu, W.; Yan, N.; Zhou, X.; Fu, J.; Guo, X.; Han, P.; Wang, J.; Liu, X., Exome sequencing identifies compound heterozygous mutations in CYP4V2 in a pedigree with retinitis pigmentosa. *PloS one* **2012,** 7, (5), e33673.

75. Lockhart, C. M.; Nakano, M.; Rettie, A. E.; Kelly, E. J., Generation and characterization of a murine model of Bietti crystalline dystrophy. *Investigative ophthalmology & visual science* **2014,** 55, (9), 5572-81.

76. Venturini, G.; Koskiniemi-Kuendig, H.; Harper, S.; Berson, E. L.; Rivolta, C., Two specific mutations are prevalent causes of recessive retinitis pigmentosa in North American patients of Jewish ancestry. *Genetics in medicine : official journal of the American College of Medical Genetics* **2015,** 17, (4), 285-90.

77. Zuchner, S.; Dallman, J.; Wen, R.; Beecham, G.; Naj, A.; Farooq, A.; Kohli, M. A.; Whitehead, P. L.; Hulme, W.; Konidari, I.; Edwards, Y. J.; Cai, G.; Peter, I.; Seo, D.; Buxbaum, J. D.; Haines, J. L.; Blanton, S.; Young, J.; Alfonso, E.; Vance, J. M.; Lam, B. L.; Pericak-Vance, M. A., Whole-exome sequencing links a variant in DHDDS to retinitis pigmentosa. *American journal of human genetics* **2011,** 88, (2), 201-6.

78. Ajmal, M.; Khan, M. I.; Neveling, K.; Khan, Y. M.; Azam, M.; Waheed, N. K.; Hamel, C. P.; Ben-Yosef, T.; De Baere, E.; Koenekoop, R. K.; Collin, R. W. J.; Qamar, R.; Cremers, F. P. M., A missense mutation in the splicing factor gene DHX38 is associated with early-onset retinitis pigmentosa with macular coloboma. *Journal of medical genetics* **2014,** 51, (7), 444-448.

79. Abu-Safieh, L.; Alrashed, M.; Anazi, S.; Alkuraya, H.; Khan, A. O.; Al-Owain, M.; Al-Zahrani, J.; Al-Abdi, L.; Hashem, M.; Al-Tarimi, S.; Sebai, M. A.; Shamia, A.; Ray-zack, M. D.; Nassan, M.; Al-Hassnan, Z. N.; Rahbeeni, Z.; Waheeb, S.; Alkharashi, A.; Abboud, E.; Al-Hazzaa, S. A. F.; Alkuraya, F. S., Autozygome-guided exome sequencing in retinal dystrophy patients reveals pathogenetic mutations and novel candidate disease genes. *Genome research* **2013,** 23, (2), 236-247.

80. Littink, K. W.; van den Born, L. I.; Koenekoop, R. K.; Collin, R. W. J.; Zonneveld, M. N.; Blokland, E. A. W.; Khan, H.; Theelen, T.; Hoyng, C. B.; Cremers, F. P. M.; den Hollander, A. I.; Klevering, B. J., Mutations in the EYS Gene Account for Approximately 5% of Autosomal Recessive Retinitis Pigmentosa and Cause a Fairly Homogeneous Phenotype. *Ophthalmology* **2010,** 117, (10), 2026-U216.

81. Arai, Y.; Maeda, A.; Hirami, Y.; Ishigami, C.; Kosugi, S.; Mandai, M.; Kurimoto, Y.; Takahashi, M., Retinitis Pigmentosa with EYS Mutations Is the Most Prevalent Inherited Retinal Dystrophy in Japanese Populations. *Journal of ophthalmology* **2015**.

82. Yu, M.; Liu, Y.; Li, J.; Natale, B. N.; Cao, S. Q.; Wang, D. L.; Amack, J. D.; Hu, H. Y., Eyes shut homolog is required for maintaining the ciliary pocket and survival of photoreceptors in zebrafish. *Biology open* **2016,** 5, (11), 1662-1673.

83. Van Schil, K.; Klevering, B. J.; Leroy, B. P.; Pott, J. W. R.; Bandah-Rozenfeld, D.; Zonneveld-Vrieling, M. N.; Sharon, D.; den Hollander, A. I.; Cremers, F. P. M.; De Baere, E.; Collin, R. W. J.; van den Born, L. I., A Nonsense Mutation in FAM161A Is a Recurrent Founder Allele in Dutch and Belgian Individuals With Autosomal Recessive Retinitis Pigmentosa. *Investigative ophthalmology & visual science* **2015,** 56, (12), 7418-7426.

84. Karlstetter, M.; Sorusch, N.; Caramoy, A.; Dannhausen, K.; Aslanidis, A.; Fauser, S.; Boesl, M. R.; Nagel-Wolfrum, K.; Tamm, E. R.; Jagle, H.; Stoehr, H.; Wolfrum, U.; Langmann, T., Disruption of the retinitis pigmentosa 28 gene Fam161a in mice affects photoreceptor ciliary structure and leads to progressive retinal degeneration. *Human molecular genetics* **2014,** 23, (19), 5197-5210.

85. Downs, L. M.; Mellersh, C. S., An Intronic SINE Insertion in FAM161A that Causes Exon-Skipping Is Associated with Progressive Retinal Atrophy in Tibetan Spaniels and Tibetan Terriers. *PloS one* **2014,** 9, (4).

86. Haer-Wigman, L.; Newman, H.; Leibu, R.; Bax, N. M.; Baris, H. N.; Rizel, L.; Banin, E.; Massarweh, A.; Roosing, S.; Lefeber, D. J.; Zonneveld-Vrieling, M. N.; Isakov, O.; Shomron, N.; Sharon, D.; Den Hollander, A. I.; Hoyng, C. B.; Cremers, F. P. M.; Ben-Yosef, T., Non-syndromic retinitis pigmentosa due to mutations in the mucopolysaccharidosis type IIIC gene, heparan-alpha-glucosaminide N-acetyltransferase (HGSNAT). *Human molecular genetics* **2015,** 24, (13), 3742-3751.

87. Hartong, D. T.; Dange, M.; Mcgee, T. L.; Berson, E. L.; Dryja, T. P.; Colman, R. F., Insights from retinitis pigmentosa into the roles of isocitrate dehydrogenases in the Krebs cycle. *Nature genetics* **2008,** 40, (10), 1230-1234.

88. Hull, S.; Owen, N.; Islam, F.; Tracey-White, D.; Plagnol, V.; Holder, G. E.; Michaelides, M.; Carss, K.; Raymond, F. L.; Rozet, J. M.; Ramsden, S. C.; Black, G. C. M.; Perrault, I.; Sarkar, A.; Moosajee, M.; Webster, A. R.; Arno, G.; Moore, A. T., Nonsyndromic Retinal Dystrophy due to Bi-Allelic Mutations in the Ciliary Transport Gene IFT140. *Investigative ophthalmology & visual science* **2016,** 57, (3), 1053-1062.

89. Miller, K. A.; Ah-Cann, C. J.; Welfare, M. F.; Tan, T. Y.; Pope, K.; Caruana, G.; Freckmann, M. L.; Savarirayan, R.; Bertram, J. F.; Dobbie, M. S.; Bateman, J. F.; Farlie, P. G., Cauli: A Mouse Strain with an Ift140 Mutation That Results in a Skeletal Ciliopathy Modelling Jeune Syndrome. *PLoS genetics* **2013,** 9, (8).

90. Bujakowska, K. M.; Zhang, Q.; Siemiatkowska, A. M.; Liu, Q.; Place, E.; Falk, M. J.; Consugar, M.; Lancelot, M. E.; Antonio, A.; Lonjou, C.; Carpentier, W.; Mohand-Said, S.; den Hollander, A. I.; Cremers, F. P. M.; Leroy, B. P.; Gai, X. W.; Sahel, J. A.; van den Born, L. I.; Collin, R. W. J.; Zeitz, C.; Audo, I.; Pierce, E. A., Mutations in IFT172 cause isolated retinal degeneration and Bardet-Biedl syndrome. *Human molecular genetics* **2015,** 24, (1), 230-242.

91. Huangfu, D. W.; Liu, A. M.; Rakeman, A. S.; Murcia, N. S.; Niswander, L.; Anderson, K. V., Hedgehog signalling in the mouse requires intraflagellar transport proteins. *Nature* **2003,** 426, (6962), 83-87.

92. van Huet, R. A. C.; Collin, R. W. J.; Siemiatkowska, A. M.; Klaver, C. C. W.; Hoyng, C. B.; Simonelli, F.; Khan, M. I.; Qamar, R.; Banin, E.; Cremers, F. P. M.; Theelen, T.; Den Hollander, A. I.; van den Born, L. I.; Klevering, B. J., IMPG2-Associated Retinitis Pigmentosa Displays Relatively Early Macular Involvement. *Investigative ophthalmology & visual science* **2014,** 55, (6), 3939-3953.

93. El Shamieh, S.; Neuille, M.; Terray, A.; Orhan, E.; Condroyer, C.; Demontant, V.; Michiels, C.; Antonio, A.; Boyard, F.; Lancelot, M. E.; Letexier, M.; Saraiva, J. P.; Leveillard, T.; Mohand-Said, S.; Goureau, O.; Sahel, J. A.; Zeitz, C.; Audo, I., Whole-Exome Sequencing Identifies KIZ as a Ciliary Gene Associated with Autosomal-Recessive Rod-Cone Dystrophy. *American Journal of Human Genetics* **2014,** 94, (4), 625-633.

94. Thompson, D. A.; Li, Y.; McHenry, C. L.; Carlson, T. J.; Ding, X.; Sieving, P. A.; Apfelstedt-Sylla, E.; Gal, A., Mutations in the gene encoding lecithin retinol acyltransferase are associated with early-onset severe retinal dystrophy. *Nature genetics* **2001,** 28, (2), 123-4.

95. Batten, M. L.; Imanishi, Y.; Maeda, T.; Tu, D. C.; Moise, A. R.; Bronson, D.; Possin, D.; Van Gelder, R. N.; Baehr, W.; Palczewski, K., Lecithin-retinol acyltransferase is essential for accumulation of all-trans-retinyl esters in the eye and in the liver. *Journal of Biological Chemistry* **2004,** 279, (11), 10422-10432.

96. van Huet, R. A.; Siemiatkowska, A. M.; Ozgul, R. K.; Yucel, D.; Hoyng, C. B.; Banin, E.; Blumenfeld, A.; Rotenstreich, Y.; Riemslag, F. C.; den Hollander, A. I.; Theelen, T.; Collin, R. W.; van den Born, L. I.; Klevering, B. J., Retinitis pigmentosa caused by mutations in the ciliary MAK gene is relatively mild and is not associated with apparent extra-ocular features. *Acta ophthalmologica* **2015,** 93, (1), 83-94.

97. Omori, Y.; Chaya, T.; Katoh, K.; Kajimura, N.; Sato, S.; Muraoka, K.; Ueno, S.; Koyasu, T.; Kondo, M.; Furukawa, T., Negative regulation of ciliary length by ciliary male germ cell-associated kinase (Mak) is required for retinal photoreceptor survival. *Proceedings of the National Academy of Sciences of the United States of America* **2010,** 107, (52), 22671-22676.

98. Scott, R. S.; McMahon, E. J.; Pop, S. M.; Reap, E. A.; Caricchio, R.; Cohen, P. L.; Earp, H. S.; Matsushima, G. K., Phagocytosis and clearance of apoptotic cells is mediated by MER. *Nature* **2001,** 411, (6834), 207-211.

99. Everson, R.; Pettitt, L.; Forman, O. P.; Dower-Tylee, O.; McLaughlin, B.; Ahonen, S.; Kaukonen, M.; Komaromy, A. M.; Lohi, H.; Mellersh, C. S.; Sansom, J.; Ricketts, S. L., An intronic LINE-1 insertion in MERTK is strongly associated with retinopathy in Swedish Vallhund dogs. *PLoS One* **2017,** 12, (8), e0183021.

100. Siemiatkowska, A. M.; van den Born, L. I.; van Hagen, P. M.; Stoffels, M.; Neveling, K.; Henkes, A.; Kipping-Geertsema, M.; Hoefsloot, L. H.; Hoyng, C. B.; Simon, A.; den Hollander, A. I.; Cremers, F. P. M.; Collin, R. W. J., Mutations in the Mevalonate Kinase (MVK) Gene Cause Nonsyndromic Retinitis Pigmentosa. *Ophthalmology* **2013,** 120, (12), 2697-2705.

101. Hager, E. J.; Tse, H. M.; Piganelli, J. D.; Gupta, M.; Baetscher, M.; Tse, T. E.; Pappu, A. S.; Steiner, R. D.; Hoffmann, G. F.; Gibson, K. M., Deletion of a single mevalonate kinase (Mvk) allele yields a murine model of hyper-IgD syndrome. *Journal of inherited metabolic disease* **2007,** 30, (6), 888-895.

102. Nishiguchi, K. M.; Tearle, R. G.; Liu, Y. F. P.; Ohd, E. C.; Miyake, N.; Benaglio, P.; Harper, S.; Koskiniemi-Kuendig, H.; Venturini, G.; Sharon, D.; Koenekoop, R. K.; Nakamura, M.; Kondo, M.; Ueno, S.; Yasuma, T. R.; Beckmann, J. S.; Ikegawa, S.; Matsumoto, N.; Terasaki, H.; Berson, E. L.; Katsanis, N.; Rivolta, C., Whole genome sequencing in patients with retinitis pigmentosa reveals pathogenic DNA structural changes and NEK2 as a new disease gene. *Proceedings of the National Academy of Sciences of the United States of America* **2013,** 110, (40), 16139-16144.

103. Sonn, S.; Khang, I.; Kim, K.; Rhee, K., Suppression of Nek2A in mouse early embryos confirms its requirement for chromosome segregation. *Journal of cell science* **2004,** 117, (23), 5557-5566.

104. Wang, F.; Li, H. J.; Xu, M. C.; Li, H.; Zhao, L.; Yang, L. Z.; Zaneveld, J. E.; Wang, K. Q.; Li, Y. M.; Sui, R. F.; Chen, R., A Homozygous Missense Mutation in NEUROD1 Is Associated With Nonsyndromic Autosomal Recessive Retinitis Pigmentosa. *Investigative ophthalmology & visual science* **2015,** 56, (1), 150-155.

105. Liu, M.; Pereira, F. A.; Price, S. D.; Chu, M. J.; Shope, C.; Himes, D.; Eatock, R. A.; Brownell, W. E.; Lysakowski, A.; Tsai, M. J., Essential role of BETA2/NeuroD1 in development of the vestibular and auditory systems. *Genes & development* **2000,** 14, (22), 2839-2854.

106. Sakamoto, K.; McCluskey, M.; Wensel, T. G.; Naggert, J. K.; Nishina, P. M., New mouse models for recessive retinitis pigmentosa caused by mutations in the Pde6a gene. *Human molecular genetics* **2009,** 18, (1), 178-192.

107. Petersen-Jones, S. M.; Entz, D. D.; Sargan, D. R., CGMP phosphodiesterase-alpha mutation causes progressive retinal atrophy in the Cardigan Welsh corgi dog. *Investigative ophthalmology & visual science* **1999,** 40, (8), 1637-1644.

108. Pichard, V.; Provost, N.; Mendes-Madeira, A.; Libeau, L.; Hulin, P.; Tshilenge, K. T.; Biget, M.; Ameline, B.; Deschamps, J. Y.; Weber, M.; Le Meur, G.; Colle, M. A.; Moullier, P.; Rolling, F., AAV-mediated Gene Therapy Halts Retinal Degeneration in PDE6beta-deficient Dogs. *Molecular therapy : the journal of the American Society of Gene Therapy* **2016,** 24, (5), 867-76.

109. Dvir, L.; Srour, G.; Abu-Ras, R.; Miller, B.; Shalev, S. A.; Ben-Yosef, T., Autosomal-recessive early-onset retinitis pigmentosa caused by a mutation in PDE6G, the gene encoding the gamma subunit of rod cGMP phosphodiesterase. *American journal of human genetics* **2010,** 87, (2), 258-64.

110. Tsang, S. H.; Gouras, P.; Yamashita, C. K.; Kjeldbye, H.; Fisher, J.; Farber, D. B.; Goff, S. P., Retinal degeneration in mice lacking the gamma subunit of the rod cGMP phosphodiesterase. *Science* **1996,** 272, (5264), 1026-9.

111. Xu, M.; Yamada, T.; Sun, Z.; Eblimit, A.; Lopez, I.; Wang, F.; Manya, H.; Xu, S.; Zhao, L.; Li, Y.; Kimchi, A.; Sharon, D.; Sui, R.; Endo, T.; Koenekoop, R. K.; Chen, R., Mutations in POMGNT1 cause non-syndromic retinitis pigmentosa. *Human molecular genetics* **2016,** 25, (8), 1479-88.

112. Liu, J.; Ball, S. L.; Yang, Y.; Mei, P.; Zhang, L.; Shi, H.; Kaminski, H. J.; Lemmon, V. P.; Hu, H., A genetic model for muscle-eye-brain disease in mice lacking protein O-mannose 1,2-N-acetylglucosaminyltransferase (POMGnT1). *Mechanisms of development* **2006,** 123, (3), 228-40.

113. Nevet, M. J.; Shalev, S. A.; Zlotogora, J.; Mazzawi, N.; Ben-Yosef, T., Identification of a prevalent founder mutation in an Israeli Muslim Arab village confirms the role of PRCD in the aetiology of retinitis pigmentosa in humans. *Journal of medical genetics* **2010,** 47, (8), 533-537.

114. Zangerl, B.; Goldstein, O.; Philp, A. R.; Lindauer, S. J. P.; Pearce-Kelling, S. E.; Mullins, R. F.; Graphodatsky, A. S.; Ripoll, D.; Felix, J. S.; Stone, E. M.; Acland, G. M.; Aguirre, G. D., Identical mutation in a novel retinal gene causes progressive rod-cone degeneration in dogs and retinitis pigmentosa in humans. *Genomics* **2006,** 88, (5), 551-563.

115. Permanyer, J.; Navarro, R.; Friedman, J.; Pomares, E.; Castro-Navarro, J.; Marfany, G.; Swaroop, A.; Gonzalez-Duarte, R., Autosomal Recessive Retinitis Pigmentosa with Early Macular Affectation Caused by Premature Truncation in PROM1. *Investigative ophthalmology & visual science* **2010,** 51, (5), 2656-2663.

116. den Hollander, A. I.; McGee, T. L.; Ziviello, C.; Banfi, S.; Dryja, T. P.; Gonzalez-Fernandez, F.; Ghosh, D.; Berson, E. L., A Homozygous Missense Mutation in the IRBP Gene (RBP3) Associated with Autosomal Recessive Retinitis Pigmentosa. *Investigative ophthalmology & visual science* **2009,** 50, (4), 1864-1872.

117. Liou, G. I.; Fei, Y. J.; Peachey, N. S.; Matragoon, S.; Wei, S. H.; Blaner, W. S.; Wang, Y. X.; Liu, C. Y.; Gottesman, M. E.; Ripps, H., Early onset photoreceptor abnormalities induced by targeted disruption of the interphotoreceptor retinoid-binding protein gene. *Journal of Neuroscience* **1998,** 18, (12), 4511-4520.

118. Arno, G.; Agrawal, S. A.; Eblimit, A.; Bellingham, J.; Xu, M.; Wang, F.; Chakarova, C.; Parfitt, D. A.; Lane, A.; Burgoyne, T.; Hull, S.; Carss, K. J.; Fiorentino, A.; Hayes, M. J.; Munro, P. M.; Nicols, R.; Pontikos, N.; Holder, G. E.; Ukirdc; Asomugha, C.; Raymond, F. L.; Moore, A. T.; Plagnol, V.; Michaelides, M.; Hardcastle, A. J.; Li, Y.; Cukras, C.; Webster, A. R.; Cheetham, M. E.; Chen, R., Mutations in REEP6 Cause Autosomal-Recessive Retinitis Pigmentosa. *Am J Hum Genet* **2016,** 99, (6), 1305-1315.

119. Agrawal, S. A.; Burgoyne, T.; Eblimit, A.; Bellingham, J.; Parfitt, D. A.; Lane, A.; Nichols, R.; Asomugha, C.; Hayes, M. J.; Munro, P. M.; Xu, M.; Wang, K.; Futter, C. E.; Li, Y.; Chen, R.; Cheetham, M. E., REEP6 deficiency leads to retinal degeneration through disruption of ER homeostasis and protein trafficking. *Hum Mol Genet* **2017,** 26, (14), 2667-2677.

120. Maeda, T.; Van Hooser, J. P.; Driessen, C. A. G. G.; Filipek, S.; Janssen, J. J. M.; Palczewski, K., Evaluation of the role of the retinal G protein-coupled receptor (RGR) in the vertebrate retina in vivo. *Journal of neurochemistry* **2003,** 85, (4), 944-956.

121. Saari, J. C.; Nawrot, M.; Kennedy, B. N.; Garwin, G. G.; Hurley, J. B.; Huang, J.; Possin, D. E.; Crabb, J. W., Visual cycle impairment in cellular retinaldehyde binding protein (CRALBP) knockout mice results in delayed dark adaptation. *Neuron* **2001,** 29, (3), 739-748.

122. Davidson, A. E.; Sergouniotis, P. I.; Mackay, D. S.; Wright, G. A.; Waseem, N. H.; Michaelides, M.; Holder, G. E.; Robson, A. G.; Moore, A. T.; Plagnol, V.; Webster, A. R., RP1L1 Variants are Associated with a Spectrum of Inherited Retinal Diseases Including Retinitis Pigmentosa and Occult Macular Dystrophy. *Human mutation* **2013,** 34, (3), 506-514.

123. Yamashita, T.; Liu, J. W.; Gao, J. G.; LeNoue, S.; Wang, C. G.; Kaminoh, J.; Bowne, S. J.; Sullivan, L. S.; Daiger, S. P.; Zhang, K.; Fitzgerald, M. E. C.; Kefalov, V. J.; Zuo, J., Essential and Synergistic Roles of RP1 and RP1L1 in Rod Photoreceptor Axoneme and Retinitis Pigmentosa. *Journal of Neuroscience* **2009,** 29, (31), 9748-9760.

124. Chen, J.; Simon, M. I.; Matthes, M. T.; Yasumura, D.; LaVail, M. M., Increased susceptibility to light damage in an arrestin knockout mouse model of Oguchi disease (stationary night blindness). *Investigative ophthalmology & visual science* **1999,** 40, (12), 2978-82.

125. Goldstein, O.; Jordan, J. A.; Aguirre, G. D.; Acland, G. M., A non-stop S-antigen gene mutation is associated with late onset hereditary retinal degeneration in dogs. *Molecular vision* **2013,** 19, 1871-1884.

126. Corton, M.; Avila-Fernandez, A.; Campello, L.; Sanchez, M.; Benavides, B.; Lopez-Molina, M. I.; Fernandez-Sanchez, L.; Sanchez-Alcudia, R.; da Silva, L. R. J.; Reyes, N.; Martin-Garrido, E.; Zurita, O.; Fernandez-San Jose, P.; Perez-Carro, R.; Garcia-Garcia, F.; Dopazo, J.; Garcia-Sandoval, B.; Cuenca, N.; Ayuso, C., Identification of the Photoreceptor Transcriptional Co-Repressor SAMD11 as Novel Cause of Autosomal Recessive Retinitis Pigmentosa. *Sci Rep* **2016,** 6, 35370.

127. Jin, Z. B.; Huang, X. F.; Lv, J. N.; Xiang, L.; Li, D. Q.; Chen, J. F.; Huang, C. J.; Wu, J. Y.; Lu, F.; Qu, J., SLC7A14 linked to autosomal recessive retinitis pigmentosa. *Nature communications* **2014,** 5.

128. Kannabiran, C.; Palavalli, L.; Jalali, S., Mutation of SPATA7 in a family with autosomal recessive early-onset retinitis pigmentosa. *J Mol Genet Med* **2012,** 6, 301-3.

129. Zhong, H.; Eblimit, A.; Moayedi, Y.; Boye, S. L.; Chiodo, V. A.; Chen, Y.; Li, Y.; Nichols, R. M.; Hauswirth, W. W.; Chen, R.; Mardon, G., AAV8(Y733F)-mediated gene therapy in a Spata7 knockout mouse model of Leber congenital amaurosis and retinitis pigmentosa. *Gene therapy* **2015,** 22, (8), 619-627.

130. Wedatilake, Y.; Niazi, R.; Fassone, E.; Powell, C. A.; Pearce, S.; Plagnol, V.; Saldanha, J. W.; Kleta, R.; Chong, W. K.; Footitt, E.; Mills, P. B.; Taanman, J. W.; Minczuk, M.; Clayton, P. T.; Rahman, S., TRNT1 deficiency: clinical, biochemical and molecular genetic features. *Orphanet journal of rare diseases* **2016,** 11, (1), 90.

131. DeLuca, A. P.; Whitmore, S. S.; Barnes, J.; Sharma, T. P.; Westfall, T. A.; Scott, C. A.; Weed, M. C.; Wiley, J. S.; Wiley, L. A.; Johnston, R. M.; Schnieders, M. J.; Lentz, S. R.; Tucker, B. A.; Mullins, R. F.; Scheetz, T. E.; Stone, E. M.; Slusarski, D. C., Hypomorphic mutations in TRNT1 cause retinitis pigmentosa with erythrocytic microcytosis. *Human molecular genetics* **2016,** 25, (1), 44-56.

132. Tadenev, A. L.; Kulaga, H. M.; May-Simera, H. L.; Kelley, M. W.; Katsanis, N.; Reed, R. R., Loss of Bardet-Biedl syndrome protein-8 (BBS8) perturbs olfactory function, protein localization, and axon targeting. *Proceedings of the National Academy of Sciences of the United States of America* **2011,** 108, (25), 10320-5.

133. Downs, L. M.; Wallin-Hakansson, B.; Bergstrom, T.; Mellersh, C. S., A novel mutation in TTC8 is associated with progressive retinal atrophy in the golden retriever. *Canine Genet Epidemiol* **2014,** 1, 4.

134. Ikeda, S.; Shiva, N.; Ikeda, A.; Smith, R. S.; Nusinowitz, S.; Yan, G.; Lin, T. R.; Chu, S.; Heckenlively, J. R.; North, M. A.; Naggert, J. K.; Nishina, P. M.; Duyao, M. P., Retinal degeneration but not obesity is observed in null mutants of the tubby-like protein 1 gene. *Human molecular genetics* **2000,** 9, (2), 155-63.

135. Liu, X. Q.; Bulgakov, O. V.; Darrow, K. N.; Pawlyk, B.; Adamian, M.; Liberman, M. C.; Li, T. S., Usherin is required for maintenance of retinal photoreceptors and normal development of cochlear hair cells. *Proceedings of the National Academy of Sciences of the United States of America* **2007,** 104, (11), 4413-4418.

136. Han, S.; Liu, X.; Xie, S.; Gao, M.; Liu, F.; Yu, S.; Sun, P.; Wang, C.; Archacki, S.; Lu, Z.; Hu, X.; Qin, Y.; Qu, Z.; Huang, Y.; Lv, Y.; Tu, J.; Li, J.; Yimer, T. A.; Jiang, T.; Tang, Z.; Luo, D.; Chen, F.; Liu, M., Knockout of ush2a gene in zebrafish causes hearing impairment and late onset rod-cone dystrophy. *Hum Genet* **2018,** 137, (10), 779-794.

137. Avila-Fernandez, A.; Perez-Carro, R.; Corton, M.; Lopez-Molina, M. I.; Campello, L.; Garanto, A.; Fernandez-Sanchez, L.; Duijkers, L.; Lopez-Martinez, M. A.; Riveiro-Alvarez, R.; Da Silva, L. R. J.; Sanchez-Alcudia, R.; Martin-Garrido, E.; Reyes, N.; Garcia-Garcia, F.; Dopazo, J.; Garcia-Sandoval, B.; Collin, R. W. J.; Cuenca, N.; Ayuso, C., Whole-exome sequencing reveals ZNF408 as a new gene associated with autosomal recessive retinitis pigmentosa with vitreal alterations. *Human molecular genetics* **2015,** 24, (14), 4037-4048.

138. Collin, R. W. J.; Nikopoulos, K.; Dona, M.; Gilissen, C.; Hoischen, A.; Boonstra, F. N.; Poulter, J. A.; Kondo, H.; Berger, W.; Toomes, C.; Tahira, T.; Mohn, L. R.; Blokland, E. A.; Hetterschijt, L.; Ali, M.; Groothuismink, J. M.; Duijkers, L.; Inglehearn, C. F.; Sollfrank, L.; Strom, T. M.; Uchio, E.; van Nouhuys, C. E.; Kremer, H.; Veltman, J. A.; van Wijk, E.; Cremers, F. P. M., ZNF408 is mutated in familial exudative vitreoretinopathy and is crucial for the development of zebrafish retinal vasculature. *Proceedings of the National Academy of Sciences of the United States of America* **2013,** 110, (24), 9856-9861.

139. Li, L.; Nakaya, N.; Chavali, V. R. M.; Ma, Z. W.; Jiao, X. D.; Sieving, P. A.; Riazuddin, S.; Tomarev, S. I.; Ayyagari, R.; Riazuddin, S. A.; Hejtmancik, J. F., A Mutation in ZNF513, a Putative Regulator of Photoreceptor Development, Causes Autosomal-Recessive Retinitis Pigmentosa. *American journal of human genetics* **2010,** 87, (3), 400-409.

140. Webb, T. R.; Parfitt, D. A.; Gardner, J. C.; Martinez, A.; Bevilacqua, D.; Davidson, A. E.; Zito, I.; Thiselton, D. L.; Ressa, J. H. C.; Apergi, M.; Schwarz, N.; Kanuga, N.; Michaelides, M.; Cheetham, M. E.; Gorin, M. B.; Hardcastle, A. J., Deep intronic mutation in OFD1, identified by targeted genomic next-generation sequencing, causes a severe form of X-linked retinitis pigmentosa (RP23). *Human molecular genetics* **2012,** 21, (16), 3647-3654.

141. Ferrante, M. I.; Zullo, A.; Barra, A.; Bimonte, S.; Messaddeq, N.; Studer, M.; Dolle, P.; Franco, B., Oral-facial-digital type I protein is required for primary cilia formation and left-right axis specification. *Nature genetics* **2006,** 38, (1), 112-117.

142. Zullo, A.; Iaconis, D.; Barra, A.; Cantone, A.; Messaddeq, N.; Capasso, G.; Dolle, P.; Igarashi, P.; Franco, B., Kidney-specific inactivation of Ofd1 leads to renal cystic disease associated with upregulation of the mTOR pathway. *Human molecular genetics* **2010,** 19, (14), 2792-2803.

143. Mookherjee, S.; Hiriyanna, S.; Kaneshiro, K.; Li, L.; Li, Y.; Li, W.; Qian, H.; Li, T.; Khanna, H.; Colosi, P.; Swaroop, A.; Wu, Z., Long-term rescue of cone photoreceptor degeneration in retinitis pigmentosa 2 (RP2)-knockout mice by gene replacement therapy. *Human molecular genetics* **2015,** 24, (22), 6446-58.

144. Thompson, D. A.; Khan, N. W.; Othman, M. I.; Chang, B.; Jia, L.; Grahek, G.; Wu, Z. J.; Hiriyanna, S.; Nellissery, J.; Li, T. S.; Khanna, H.; Colosi, P.; Swaroop, A.; Heckenlively, J. R., Rd9 Is a Naturally Occurring Mouse Model of a Common Form of Retinitis Pigmentosa Caused by Mutations in RPGR-ORF15. *PloS one* **2012,** 7, (5).

145. Appelbaum, T.; Becker, D.; Santana, E.; Aguirre, G. D., Molecular studies of phenotype variation in canine RPGR-XLPRA1. *Molecular vision* **2016,** 22, 319-331.

146. Forsythe, E.; Beales, P. L., Bardet-Biedl Syndrome. In *GeneReviews((R))*, Adam, M. P.; Ardinger, H. H.; Pagon, R. A.; Wallace, S. E.; Bean, L. J. H.; Stephens, K.; Amemiya, A., Eds. Seattle (WA), 1993.

147. Loktev, A. V.; Zhang, Q.; Beck, J. S.; Searby, C. C.; Scheetz, T. E.; Bazan, J. F.; Slusarski, D. C.; Sheffield, V. C.; Jackson, P. K.; Nachury, M. V., A BBSome subunit links ciliogenesis, microtubule stability, and acetylation. *Dev Cell* **2008,** 15, (6), 854-65.

148. Katsanis, N., The oligogenic properties of Bardet-Biedl syndrome. *Hum Mol Genet* **2004,** 13 Spec No 1, R65-71.

149. Swiderski, R. E.; Nishimura, D. Y.; Mullins, R. F.; Olvera, M. A.; Ross, J. L.; Huang, J.; Stone, E. M.; Sheffield, V. C., Gene expression analysis of photoreceptor cell loss in bbs4-knockout mice reveals an early stress gene response and photoreceptor cell damage. *Invest Ophthalmol Vis Sci* **2007,** 48, (7), 3329-40.

150. Chew, T.; Haase, B.; Bathgate, R.; Willet, C. E.; Kaukonen, M. K.; Mascord, L. J.; Lohi, H. T.; Wade, C. M., A Coding Variant in the Gene Bardet-Biedl Syndrome 4 (BBS4) Is Associated with a Novel Form of Canine Progressive Retinal Atrophy. *G3 (Bethesda)* **2017,** 7, (7), 2327-2335.

151. Kretschmer, V.; Patnaik, S. R.; Kretschmer, F.; Chawda, M. M.; Hernandez-Hernandez, V.; May-Simera, H. L., Progressive Characterization of Visual Phenotype in Bardet-Biedl Syndrome Mutant Mice. *Invest Ophthalmol Vis Sci* **2019,** 60, (4), 1132-1143.

152. Zhang, Q.; Nishimura, D.; Vogel, T.; Shao, J.; Swiderski, R.; Yin, T.; Searby, C.; Carter, C. S.; Kim, G.; Bugge, K.; Stone, E. M.; Sheffield, V. C., BBS7 is required for BBSome formation and its absence in mice results in Bardet-Biedl syndrome phenotypes and selective abnormalities in membrane protein trafficking. *J Cell Sci* **2013,** 126, (Pt 11), 2372-80.

153. Peterson, S. M.; McGill, T. J.; Puthussery, T.; Stoddard, J.; Renner, L.; Lewis, A. D.; Colgin, L. M. A.; Gayet, J.; Wang, X.; Prongay, K.; Cullin, C.; Dozier, B.; Ferguson, B.; Neuringer, M., Bardet-Biedl Syndrome in rhesus macaques: A nonhuman primate model of retinitis pigmentosa. *Exp Eye Res* **2019**, 107825.

154. Nishimura, D. Y.; Swiderski, R. E.; Searby, C. C.; Berg, E. M.; Ferguson, A. L.; Hennekam, R.; Merin, S.; Weleber, R. G.; Biesecker, L. G.; Stone, E. M.; Sheffield, V. C., Comparative genomics and gene expression analysis identifies BBS9, a new Bardet-Biedl syndrome gene. *Am J Hum Genet* **2005,** 77, (6), 1021-33.

155. Veleri, S.; Bishop, K.; Dalle Nogare, D. E.; English, M. A.; Foskett, T. J.; Chitnis, A.; Sood, R.; Liu, P.; Swaroop, A., Knockdown of Bardet-Biedl syndrome gene BBS9/PTHB1 leads to cilia defects. *PLoS One* **2012,** 7, (3), e34389.

156. Stoetzel, C.; Laurier, V.; Davis, E. E.; Muller, J.; Rix, S.; Badano, J. L.; Leitch, C. C.; Salem, N.; Chouery, E.; Corbani, S.; Jalk, N.; Vicaire, S.; Sarda, P.; Hamel, C.; Lacombe, D.; Holder, M.; Odent, S.; Holder, S.; Brooks, A. S.; Elcioglu, N. H.; Silva, E. D.; Rossillion, B.; Sigaudy, S.; de Ravel, T. J.; Lewis, R. A.; Leheup, B.; Verloes, A.; Amati-Bonneau, P.; Megarbane, A.; Poch, O.; Bonneau, D.; Beales, P. L.; Mandel, J. L.; Katsanis, N.; Dollfus, H., BBS10 encodes a vertebrate-specific chaperonin-like protein and is a major BBS locus. *Nat Genet* **2006,** 38, (5), 521-4.

157. Cognard, N.; Scerbo, M. J.; Obringer, C.; Yu, X.; Costa, F.; Haser, E.; Le, D.; Stoetzel, C.; Roux, M. J.; Moulin, B.; Dollfus, H.; Marion, V., Comparing the Bbs10 complete knockout phenotype with a specific renal epithelial knockout one highlights the link between renal defects and systemic inactivation in mice. *Cilia* **2015,** 4, 10.

158. Stoetzel, C.; Muller, J.; Laurier, V.; Davis, E. E.; Zaghloul, N. A.; Vicaire, S.; Jacquelin, C.; Plewniak, F.; Leitch, C. C.; Sarda, P.; Hamel, C.; de Ravel, T. J.; Lewis, R. A.; Friederich, E.; Thibault, C.; Danse, J. M.; Verloes, A.; Bonneau, D.; Katsanis, N.; Poch, O.; Mandel, J. L.; Dollfus, H., Identification of a novel BBS gene (BBS12) highlights the major role of a vertebrate-specific branch of chaperonin-related proteins in Bardet-Biedl syndrome. *Am J Hum Genet* **2007,** 80, (1), 1-11.

159. Marion, V.; Mockel, A.; De Melo, C.; Obringer, C.; Claussmann, A.; Simon, A.; Messaddeq, N.; Durand, M.; Dupuis, L.; Loeffler, J. P.; King, P.; Mutter-Schmidt, C.; Petrovsky, N.; Stoetzel, C.; Dollfus, H., BBS-induced ciliary defect enhances adipogenesis, causing paradoxical higher-insulin sensitivity, glucose usage, and decreased inflammatory response. *Cell Metab* **2012,** 16, (3), 363-77.

160. Khan, A. O.; Decker, E.; Bachmann, N.; Bolz, H. J.; Bergmann, C., C8orf37 is mutated in Bardet-Biedl syndrome and constitutes a locus allelic to non-syndromic retinal dystrophies. *Ophthalmic Genet* **2016,** 37, (3), 290-3.

161. Yildiz Bolukbasi, E.; Mumtaz, S.; Afzal, M.; Woehlbier, U.; Malik, S.; Tolun, A., Homozygous mutation in CEP19, a gene mutated in morbid obesity, in Bardet-Biedl syndrome with predominant postaxial polydactyly. *J Med Genet* **2018,** 55, (3), 189-197.

162. Shalata, A.; Ramirez, M. C.; Desnick, R. J.; Priedigkeit, N.; Buettner, C.; Lindtner, C.; Mahroum, M.; Abdul-Ghani, M.; Dong, F.; Arar, N.; Camacho-Vanegas, O.; Zhang, R.; Camacho, S. C.; Chen, Y.; Ibdah, M.; DeFronzo, R.; Gillespie, V.; Kelley, K.; Dynlacht, B. D.; Kim, S.; Glucksman, M. J.; Borochowitz, Z. U.; Martignetti, J. A., Morbid obesity resulting from inactivation of the ciliary protein CEP19 in humans and mice. *Am J Hum Genet* **2013,** 93, (6), 1061-71.

163. Zhang, Y.; Seo, S.; Bhattarai, S.; Bugge, K.; Searby, C. C.; Zhang, Q.; Drack, A. V.; Stone, E. M.; Sheffield, V. C., BBS mutations modify phenotypic expression of CEP290-related ciliopathies. *Hum Mol Genet* **2014,** 23, (1), 40-51.

164. Chang, B.; Khanna, H.; Hawes, N.; Jimeno, D.; He, S.; Lillo, C.; Parapuram, S. K.; Cheng, H.; Scott, A.; Hurd, R. E.; Sayer, J. A.; Otto, E. A.; Attanasio, M.; O'Toole, J. F.; Jin, G.; Shou, C.; Hildebrandt, F.; Williams, D. S.; Heckenlively, J. R.; Swaroop, A., In-frame deletion in a novel centrosomal/ciliary protein CEP290/NPHP6 perturbs its interaction with RPGR and results in early-onset retinal degeneration in the rd16 mouse. *Hum Mol Genet* **2006,** 15, (11), 1847-57.

165. Lancaster, M. A.; Gopal, D. J.; Kim, J.; Saleem, S. N.; Silhavy, J. L.; Louie, C. M.; Thacker, B. E.; Williams, Y.; Zaki, M. S.; Gleeson, J. G., Defective Wnt-dependent cerebellar midline fusion in a mouse model of Joubert syndrome. *Nat Med* **2011,** 17, (6), 726-31.

166. Menotti-Raymond, M.; David, V. A.; Schaffer, A. A.; Stephens, R.; Wells, D.; Kumar-Singh, R.; O'Brien, S. J.; Narfstrom, K., Mutation in CEP290 discovered for cat model of human retinal degeneration. *J Hered* **2007,** 98, (3), 211-20.

167. Bujakowska, K. M.; Zhang, Q.; Siemiatkowska, A. M.; Liu, Q.; Place, E.; Falk, M. J.; Consugar, M.; Lancelot, M. E.; Antonio, A.; Lonjou, C.; Carpentier, W.; Mohand-Said, S.; den Hollander, A. I.; Cremers, F. P.; Leroy, B. P.; Gai, X.; Sahel, J. A.; van den Born, L. I.; Collin, R. W.; Zeitz, C.; Audo, I.; Pierce, E. A., Mutations in IFT172 cause isolated retinal degeneration and Bardet-Biedl syndrome. *Hum Mol Genet* **2015,** 24, (1), 230-42.

168. Huangfu, D.; Liu, A.; Rakeman, A. S.; Murcia, N. S.; Niswander, L.; Anderson, K. V., Hedgehog signalling in the mouse requires intraflagellar transport proteins. *Nature* **2003,** 426, (6962), 83-7.

169. Halbritter, J.; Bizet, A. A.; Schmidts, M.; Porath, J. D.; Braun, D. A.; Gee, H. Y.; McInerney-Leo, A. M.; Krug, P.; Filhol, E.; Davis, E. E.; Airik, R.; Czarnecki, P. G.; Lehman, A. M.; Trnka, P.; Nitschke, P.; Bole-Feysot, C.; Schueler, M.; Knebelmann, B.; Burtey, S.; Szabo, A. J.; Tory, K.; Leo, P. J.; Gardiner, B.; McKenzie, F. A.; Zankl, A.; Brown, M. A.; Hartley, J. L.; Maher, E. R.; Li, C.; Leroux, M. R.; Scambler, P. J.; Zhan, S. H.; Jones, S. J.; Kayserili, H.; Tuysuz, B.; Moorani, K. N.; Constantinescu, A.; Krantz, I. D.; Kaplan, B. S.; Shah, J. V.; Consortium, U. K.; Hurd, T. W.; Doherty, D.; Katsanis, N.; Duncan, E. L.; Otto, E. A.; Beales, P. L.; Mitchison, H. M.; Saunier, S.; Hildebrandt, F., Defects in the IFT-B component IFT172 cause Jeune and Mainzer-Saldino syndromes in humans. *Am J Hum Genet* **2013,** 93, (5), 915-25.

170. Aldahmesh, M. A.; Li, Y.; Alhashem, A.; Anazi, S.; Alkuraya, H.; Hashem, M.; Awaji, A. A.; Sogaty, S.; Alkharashi, A.; Alzahrani, S.; Al Hazzaa, S. A.; Xiong, Y.; Kong, S.; Sun, Z.; Alkuraya, F. S., IFT27, encoding a small GTPase component of IFT particles, is mutated in a consanguineous family with Bardet-Biedl syndrome. *Hum Mol Genet* **2014,** 23, (12), 3307-15.

171. Zhang, Y.; Liu, H.; Li, W.; Zhang, Z.; Shang, X.; Zhang, D.; Li, Y.; Zhang, S.; Liu, J.; Hess, R. A.; Pazour, G. J.; Zhang, Z., Intraflagellar transporter protein (IFT27), an IFT25 binding partner, is essential for male fertility and spermiogenesis in mice. *Dev Biol* **2017,** 432, (1), 125-139.

172. Jacoby, M.; Cox, J. J.; Gayral, S.; Hampshire, D. J.; Ayub, M.; Blockmans, M.; Pernot, E.; Kisseleva, M. V.; Compere, P.; Schiffmann, S. N.; Gergely, F.; Riley, J. H.; Perez-Morga, D.; Woods, C. G.; Schurmans, S., INPP5E mutations cause primary cilium signaling defects, ciliary instability and ciliopathies in human and mouse. *Nat Genet* **2009,** 41, (9), 1027-31.

173. Lindstrand, A.; Davis, E. E.; Carvalho, C. M.; Pehlivan, D.; Willer, J. R.; Tsai, I. C.; Ramanathan, S.; Zuppan, C.; Sabo, A.; Muzny, D.; Gibbs, R.; Liu, P.; Lewis, R. A.; Banin, E.; Lupski, J. R.; Clark, R.; Katsanis, N., Recurrent CNVs and SNVs at the NPHP1 locus contribute pathogenic alleles to Bardet-Biedl syndrome. *Am J Hum Genet* **2014,** 94, (5), 745-54.

174. Zhong, H.; Chen, Y.; Li, Y.; Chen, R.; Mardon, G., CRISPR-engineered mosaicism rapidly reveals that loss of Kcnj13 function in mice mimics human disease phenotypes. *Sci Rep* **2015,** 5, 8366.

175. Toms, M.; Dubis, A. M.; Lim, W. S.; Webster, A. R.; Gorin, M. B.; Moosajee, M., Missense variants in the conserved transmembrane M2 protein domain of KCNJ13 associated with retinovascular changes in humans and zebrafish. *Exp Eye Res* **2019,** 189, 107852.

176. Marion, V.; Stutzmann, F.; Gerard, M.; De Melo, C.; Schaefer, E.; Claussmann, A.; Helle, S.; Delague, V.; Souied, E.; Barrey, C.; Verloes, A.; Stoetzel, C.; Dollfus, H., Exome sequencing identifies mutations in LZTFL1, a BBSome and smoothened trafficking regulator, in a family with Bardet--Biedl syndrome with situs inversus and insertional polydactyly. *J Med Genet* **2012,** 49, (5), 317-21.

177. Jiang, J.; Promchan, K.; Jiang, H.; Awasthi, P.; Marshall, H.; Harned, A.; Natarajan, V., Depletion of BBS Protein LZTFL1 Affects Growth and Causes Retinal Degeneration in Mice. *J Genet Genomics* **2016,** 43, (6), 381-91.

178. Fath, M. A.; Mullins, R. F.; Searby, C.; Nishimura, D. Y.; Wei, J.; Rahmouni, K.; Davis, R. E.; Tayeh, M. K.; Andrews, M.; Yang, B.; Sigmund, C. D.; Stone, E. M.; Sheffield, V. C., Mkks-null mice have a phenotype resembling Bardet-Biedl syndrome. *Hum Mol Genet* **2005,** 14, (9), 1109-18.

179. Leitch, C. C.; Zaghloul, N. A.; Davis, E. E.; Stoetzel, C.; Diaz-Font, A.; Rix, S.; Alfadhel, M.; Lewis, R. A.; Eyaid, W.; Banin, E.; Dollfus, H.; Beales, P. L.; Badano, J. L.; Katsanis, N., Hypomorphic mutations in syndromic encephalocele genes are associated with Bardet-Biedl syndrome. *Nat Genet* **2008,** 40, (4), 443-8.

180. Weatherbee, S. D.; Niswander, L. A.; Anderson, K. V., A mouse model for Meckel syndrome reveals Mks1 is required for ciliogenesis and Hedgehog signaling. *Hum Mol Genet* **2009,** 18, (23), 4565-75.

181. Jiang, S. T.; Chiou, Y. Y.; Wang, E.; Chien, Y. L.; Ho, H. H.; Tsai, F. J.; Lin, C. Y.; Tsai, S. P.; Li, H., Essential role of nephrocystin in photoreceptor intraflagellar transport in mouse. *Hum Mol Genet* **2009,** 18, (9), 1566-77.

182. Weihbrecht, K.; Goar, W. A.; Carter, C. S.; Sheffield, V. C.; Seo, S., Genotypic and phenotypic characterization of the Sdccag8Tn(sb-Tyr)2161B.CA1C2Ove mouse model. *PLoS One* **2018,** 13, (2), e0192755.

183. Otto, E. A.; Hurd, T. W.; Airik, R.; Chaki, M.; Zhou, W.; Stoetzel, C.; Patil, S. B.; Levy, S.; Ghosh, A. K.; Murga-Zamalloa, C. A.; van Reeuwijk, J.; Letteboer, S. J.; Sang, L.; Giles, R. H.; Liu, Q.; Coene, K. L.; Estrada-Cuzcano, A.; Collin, R. W.; McLaughlin, H. M.; Held, S.; Kasanuki, J. M.; Ramaswami, G.; Conte, J.; Lopez, I.; Washburn, J.; Macdonald, J.; Hu, J.; Yamashita, Y.; Maher, E. R.; Guay-Woodford, L. M.; Neumann, H. P.; Obermuller, N.; Koenekoop, R. K.; Bergmann, C.; Bei, X.; Lewis, R. A.; Katsanis, N.; Lopes, V.; Williams, D. S.; Lyons, R. H.; Dang, C. V.; Brito, D. A.; Dias, M. B.; Zhang, X.; Cavalcoli, J. D.; Nurnberg, G.; Nurnberg, P.; Pierce, E. A.; Jackson, P. K.; Antignac,

C.; Saunier, S.; Roepman, R.; Dollfus, H.; Khanna, H.; Hildebrandt, F., Candidate exome capture identifies mutation of SDCCAG8 as the cause of a retinal-renal ciliopathy. *Nat Genet* **2010,** 42, (10), 840-50.

184. Kudryashova, E.; Wu, J.; Havton, L. A.; Spencer, M. J., Deficiency of the E3 ubiquitin ligase TRIM32 in mice leads to a myopathy with a neurogenic component. *Hum Mol Genet* **2009,** 18, (7), 1353-67.

185. Freund, C. L.; Wang, Q. L.; Chen, S.; Muskat, B. L.; Wiles, C. D.; Sheffield, V. C.; Jacobson, S. G.; McInnes, R. R.; Zack, D. J.; Stone, E. M., De novo mutations in the CRX homeobox gene associated with Leber congenital amaurosis. *Nat Genet* **1998,** 18, (4), 311-2.

186. Bowne, S. J.; Sullivan, L. S.; Mortimer, S. E.; Hedstrom, L.; Zhu, J.; Spellicy, C. J.; Gire, A. I.; Hughbanks-Wheaton, D.; Birch, D. G.; Lewis, R. A.; Heckenlively, J. R.; Daiger, S. P., Spectrum and frequency of mutations in IMPDH1 associated with autosomal dominant retinitis pigmentosa and leber congenital amaurosis. *Invest Ophthalmol Vis Sci* **2006,** 47, (1), 34-42.

187. Roger, J. E.; Hiriyanna, A.; Gotoh, N.; Hao, H.; Cheng, D. F.; Ratnapriya, R.; Kautzmann, M. A.; Chang, B.; Swaroop, A., OTX2 loss causes rod differentiation defect in CRX-associated congenital blindness. *J Clin Invest* **2014,** 124, (2), 631-43.

188. Beby, F.; Housset, M.; Fossat, N.; Le Greneur, C.; Flamant, F.; Godement, P.; Lamonerie, T., Otx2 gene deletion in adult mouse retina induces rapid RPE dystrophy and slow photoreceptor degeneration. *PLoS One* **2010,** 5, (7), e11673.

189. Kondkar, A. A.; Abu-Amero, K. K., Leber congenital amaurosis: Current genetic basis, scope for genetic testing and personalized medicine. *Exp Eye Res* **2019,** 189, 107834.

190. Ramamurthy, V.; Niemi, G. A.; Reh, T. A.; Hurley, J. B., Leber congenital amaurosis linked to AIPL1: a mouse model reveals destabilization of cGMP phosphodiesterase. *Proc Natl Acad Sci U S A* **2004,** 101, (38), 13897-902.

191. Aldahmesh, M. A.; Al-Owain, M.; Alqahtani, F.; Hazzaa, S.; Alkuraya, F. S., A null mutation in CABP4 causes Leber's congenital amaurosis-like phenotype. *Mol Vis* **2010,** 16, 207-12.

192. Haeseleer, F.; Imanishi, Y.; Maeda, T.; Possin, D. E.; Maeda, A.; Lee, A.; Rieke, F.; Palczewski, K., Essential role of Ca2+-binding protein 4, a Cav1.4 channel regulator, in photoreceptor synaptic function. *Nat Neurosci* **2004,** 7, (10), 1079-87.

193. Minegishi, Y.; Sheng, X.; Yoshitake, K.; Sergeev, Y.; Iejima, D.; Shibagaki, Y.; Monma, N.; Ikeo, K.; Furuno, M.; Zhuang, W.; Liu, Y.; Rong, W.; Hattori, S.; Iwata, T., CCT2 Mutations Evoke Leber Congenital Amaurosis due to Chaperone Complex Instability. *Sci Rep* **2016,** 6, 33742.

194. Minegishi, Y.; Nakaya, N.; Tomarev, S. I., Mutation in the Zebrafish cct2 Gene Leads to Abnormalities of Cell Cycle and Cell Death in the Retina: A Model of CCT2-Related Leber Congenital Amaurosis. *Invest Ophthalmol Vis Sci* **2018,** 59, (2), 995-1004.

195. Soens, Z. T.; Li, Y.; Zhao, L.; Eblimit, A.; Dharmat, R.; Li, Y.; Chen, Y.; Naqeeb, M.; Fajardo, N.; Lopez, I.; Sun, Z.; Koenekoop, R. K.; Chen, R., Hypomorphic mutations identified in the candidate Leber congenital amaurosis gene CLUAP1. *Genet Med* **2016,** 18, (10), 1044-51.

196. Botilde, Y.; Yoshiba, S.; Shinohara, K.; Hasegawa, T.; Nishimura, H.; Shiratori, H.; Hamada, H., Cluap1 localizes preferentially to the base and tip of cilia and is required for ciliogenesis in the mouse embryo. *Dev Biol* **2013,** 381, (1), 203-12.

197. Lee, C.; Wallingford, J. B.; Gross, J. M., Cluap1 is essential for ciliogenesis and photoreceptor maintenance in the vertebrate eye. *Invest Ophthalmol Vis Sci* **2014,** 55, (7), 4585-92.

198. Abu-Safieh, L.; Alrashed, M.; Anazi, S.; Alkuraya, H.; Khan, A. O.; Al-Owain, M.; Al-Zahrani, J.; Al-Abdi, L.; Hashem, M.; Al-Tarimi, S.; Sebai, M. A.; Shamia, A.; Ray-Zack, M. D.; Nassan, M.; Al-Hassnan, Z. N.; Rahbeeni, Z.; Waheeb, S.; Alkharashi, A.; Abboud, E.; Al-Hazzaa, S. A.; Alkuraya, F. S., Autozygome-guided exome sequencing in retinal dystrophy patients reveals pathogenetic mutations and novel candidate disease genes. *Genome Res* **2013,** 23, (2), 236-47.

199. Asai-Coakwell, M.; March, L.; Dai, X. H.; Duval, M.; Lopez, I.; French, C. R.; Famulski, J.; De Baere, E.; Francis, P. J.; Sundaresan, P.; Sauve, Y.; Koenekoop, R. K.; Berry, F. B.; Allison, W. T.; Waskiewicz, A. J.; Lehmann, O. J., Contribution of growth differentiation factor 6-dependent cell survival to early-onset retinal dystrophies. *Hum Mol Genet* **2013,** 22, (7), 1432-42.

200. Asai-Coakwell, M.; French, C. R.; Berry, K. M.; Ye, M.; Koss, R.; Somerville, M.; Mueller, R.; van Heyningen, V.; Waskiewicz, A. J.; Lehmann, O. J., GDF6, a novel locus for a spectrum of ocular developmental anomalies. *Am J Hum Genet* **2007,** 80, (2), 306-15.

201. Yang, R. B.; Robinson, S. W.; Xiong, W. H.; Yau, K. W.; Birch, D. G.; Garbers, D. L., Disruption of a retinal guanylyl cyclase gene leads to cone-specific dystrophy and paradoxical rod behavior. *J Neurosci* **1999,** 19, (14), 5889-97.

202. Larkin, P.; Semple-Rowland, S. L., A null mutation in guanylate cyclase-1 alters the temporal dynamics and light entrainment properties of the iodopsin rhythm in cone photoreceptor cells. *Brain Res Mol Brain Res* **2001,** 92, (1-2), 49-57.

203. Xu, M.; Yang, L.; Wang, F.; Li, H.; Wang, X.; Wang, W.; Ge, Z.; Wang, K.; Zhao, L.; Li, H.; Li, Y.; Sui, R.; Chen, R., Mutations in human IFT140 cause non-syndromic retinal degeneration. *Hum Genet* **2015,** 134, (10), 1069-78.

204. Estrada-Cuzcano, A.; Koenekoop, R. K.; Coppieters, F.; Kohl, S.; Lopez, I.; Collin, R. W.; De Baere, E. B.; Roeleveld, D.; Marek, J.; Bernd, A.; Rohrschneider, K.; van den Born, L. I.; Meire, F.; Maumenee, I. H.; Jacobson, S. G.; Hoyng, C. B.; Zrenner, E.; Cremers, F. P.; den Hollander, A. I., IQCB1 mutations in patients with leber congenital amaurosis. *Invest Ophthalmol Vis Sci* **2011,** 52, (2), 834-9.

205. Ronquillo, C. C.; Hanke-Gogokhia, C.; Revelo, M. P.; Frederick, J. M.; Jiang, L.; Baehr, W., Ciliopathy-associated IQCB1/NPHP5 protein is required for mouse photoreceptor outer segment formation. *FASEB J* **2016,** 30, (10), 3400-3412.

206. Schafer, T.; Putz, M.; Lienkamp, S.; Ganner, A.; Bergbreiter, A.; Ramachandran, H.; Gieloff, V.; Gerner, M.; Mattonet, C.; Czarnecki, P. G.; Sayer, J. A.; Otto, E. A.; Hildebrandt, F.; Kramer-Zucker, A.; Walz, G., Genetic and physical interaction between the NPHP5 and NPHP6 gene products. *Hum Mol Genet* **2008,** 17, (23), 3655-62.

207. Downs, L. M.; Scott, E. M.; Cideciyan, A. V.; Iwabe, S.; Dufour, V.; Gardiner, K. L.; Genini, S.; Marinho, L. F.; Sumaroka, A.; Kosyk, M. S.; Swider, M.; Aguirre, G. K.; Jacobson, S. G.; Beltran, W. A.; Aguirre, G. D., Overlap of abnormal photoreceptor development and progressive degeneration in Leber congenital amaurosis caused by NPHP5 mutation. *Hum Mol Genet* **2016,** 25, (19), 4211-4226.

208. Sergouniotis, P. I.; Davidson, A. E.; Mackay, D. S.; Li, Z.; Yang, X.; Plagnol, V.; Moore, A. T.; Webster, A. R., Recessive mutations in KCNJ13, encoding an inwardly rectifying potassium channel subunit, cause leber congenital amaurosis. *Am J Hum Genet* **2011,** 89, (1), 183-90.

209. den Hollander, A. I.; Koenekoop, R. K.; Mohamed, M. D.; Arts, H. H.; Boldt, K.; Towns, K. V.; Sedmak, T.; Beer, M.; Nagel-Wolfrum, K.; McKibbin, M.; Dharmaraj, S.; Lopez, I.; Ivings, L.; Williams, G. A.; Springell, K.; Woods, C. G.; Jafri, H.; Rashid, Y.; Strom, T. M.; van der Zwaag, B.; Gosens, I.; Kersten, F. F.; van Wijk, E.; Veltman, J. A.; Zonneveld, M. N.; van Beersum, S. E.; Maumenee, I. H.; Wolfrum, U.; Cheetham, M. E.; Ueffing, M.; Cremers, F. P.; Inglehearn, C. F.; Roepman, R., Mutations in LCA5, encoding the ciliary protein lebercilin, cause Leber congenital amaurosis. *Nat Genet* **2007,** 39, (7), 889-95.

210. Boldt, K.; Mans, D. A.; Won, J.; van Reeuwijk, J.; Vogt, A.; Kinkl, N.; Letteboer, S. J.; Hicks, W. L.; Hurd, R. E.; Naggert, J. K.; Texier, Y.; den Hollander, A. I.; Koenekoop, R. K.; Bennett, J.; Cremers, F. P.; Gloeckner, C. J.; Nishina, P. M.; Roepman, R.; Ueffing, M., Disruption of intraflagellar protein transport in photoreceptor cilia causes Leber congenital amaurosis in humans and mice. *J Clin Invest* **2011,** 121, (6), 2169-80.

211. Qu, Z.; Yimer, T. A.; Xie, S.; Wong, F.; Yu, S.; Liu, X.; Han, S.; Ma, J.; Lu, Z.; Hu, X.; Qin, Y.; Huang, Y.; Lv, Y.; Li, J.; Tang, Z.; Liu, F.; Liu, M., Knocking out lca5 in zebrafish causes cone-rod dystrophy due to impaired outer segment protein trafficking. *Biochim Biophys Acta Mol Basis Dis* **2019,** 1865, (10), 2694-2705.

212. Sears, A. E.; Palczewski, K., Lecithin:Retinol Acyltransferase: A Key Enzyme Involved in the Retinoid (visual) Cycle. *Biochemistry* **2016,** 55, (22), 3082-91.

213. Falk, M. J.; Zhang, Q.; Nakamaru-Ogiso, E.; Kannabiran, C.; Fonseca-Kelly, Z.; Chakarova, C.; Audo, I.; Mackay, D. S.; Zeitz, C.; Borman, A. D.; Staniszewska, M.; Shukla, R.; Palavalli, L.; Mohand-Said, S.; Waseem, N. H.; Jalali, S.; Perin, J. C.; Place, E.; Ostrovsky, J.; Xiao, R.; Bhattacharya, S. S.; Consugar, M.; Webster, A. R.; Sahel, J. A.; Moore, A. T.; Berson, E. L.; Liu, Q.; Gai, X.; Pierce, E. A., NMNAT1 mutations cause Leber congenital amaurosis. *Nat Genet* **2012,** 44, (9), 1040-5.

214. Koenekoop, R. K.; Wang, H.; Majewski, J.; Wang, X.; Lopez, I.; Ren, H.; Chen, Y.; Li, Y.; Fishman, G. A.; Genead, M.; Schwartzentruber, J.; Solanki, N.; Traboulsi, E. I.; Cheng, J.; Logan, C. V.; McKibbin, M.; Hayward, B. E.; Parry, D. A.; Johnson, C. A.; Nageeb, M.; Finding of Rare Disease Genes Canada, C.; Poulter, J. A.; Mohamed, M. D.; Jafri, H.; Rashid, Y.; Taylor, G. R.; Keser, V.; Mardon, G.; Xu, H.; Inglehearn, C. F.; Fu, Q.; Toomes, C.; Chen, R., Mutations in NMNAT1 cause Leber congenital amaurosis and identify a new disease pathway for retinal degeneration. *Nat Genet* **2012,** 44, (9), 1035-9.

215. Chiang, P. W.; Wang, J.; Chen, Y.; Fu, Q.; Zhong, J.; Chen, Y.; Yi, X.; Wu, R.; Gan, H.; Shi, Y.; Chen, Y.; Barnett, C.; Wheaton, D.; Day, M.; Sutherland, J.; Heon, E.; Weleber, R. G.; Gabriel, L. A.; Cong, P.; Chuang, K.; Ye, S.; Sallum, J. M.; Qi, M., Exome sequencing identifies NMNAT1 mutations as a cause of Leber congenital amaurosis. *Nat Genet* **2012,** 44, (9), 972-4.

216. Avery, M. A.; Sheehan, A. E.; Kerr, K. S.; Wang, J.; Freeman, M. R., Wld S requires Nmnat1 enzymatic activity and N16-VCP interactions to suppress Wallerian degeneration. *J Cell Biol* **2009,** 184, (4), 501-13.

217. Zhai, R. G.; Cao, Y.; Hiesinger, P. R.; Zhou, Y.; Mehta, S. Q.; Schulze, K. L.; Verstreken, P.; Bellen, H. J., Drosophila NMNAT maintains neural integrity independent of its NAD synthesis activity. *PLoS Biol* **2006,** 4, (12), e416.

218. Wang, X.; Wang, H.; Sun, V.; Tuan, H. F.; Keser, V.; Wang, K.; Ren, H.; Lopez, I.; Zaneveld, J. E.; Siddiqui, S.; Bowles, S.; Khan, A.; Salvo, J.; Jacobson, S. G.; Iannaccone, A.; Wang, F.; Birch, D.; Heckenlively, J. R.; Fishman, G. A.; Traboulsi, E. I.; Li, Y.; Wheaton, D.; Koenekoop, R. K.; Chen, R., Comprehensive molecular diagnosis of 179 Leber congenital amaurosis and juvenile retinitis pigmentosa patients by targeted next generation sequencing. *J Med Genet* **2013,** 50, (10), 674-88.

219. Chang, B.; Heckenlively, J. R.; Hawes, N. L.; Roderick, T. H., New mouse primary retinal degeneration (rd-3). *Genomics* **1993,** 16, (1), 45-9.

220. Zhao, Y.; Hong, D. H.; Pawlyk, B.; Yue, G.; Adamian, M.; Grynberg, M.; Godzik, A.; Li, T., The retinitis pigmentosa GTPase regulator (RPGR)- interacting protein: subserving RPGR function and participating in disk morphogenesis. *Proc Natl Acad Sci U S A* **2003,** 100, (7), 3965-70.

221. Mellersh, C. S.; Boursnell, M. E.; Pettitt, L.; Ryder, E. J.; Holmes, N. G.; Grafham, D.; Forman, O. P.; Sampson, J.; Barnett, K. C.; Blanton, S.; Binns, M. M.; Vaudin, M., Canine RPGRIP1 mutation establishes cone-rod dystrophy in miniature longhaired dachshunds as a homologue of human Leber congenital amaurosis. *Genomics* **2006,** 88, (3), 293-301.

222. Wang, H.; den Hollander, A. I.; Moayedi, Y.; Abulimiti, A.; Li, Y.; Collin, R. W.; Hoyng, C. B.; Lopez, I.; Abboud, E. B.; Al-Rajhi, A. A.; Bray, M.; Lewis, R. A.; Lupski, J. R.; Mardon, G.; Koenekoop, R. K.; Chen, R., Mutations in SPATA7 cause Leber congenital amaurosis and juvenile retinitis pigmentosa. *Am J Hum Genet* **2009,** 84, (3), 380-7.

223. Mataftsi, A.; Schorderet, D. F.; Chachoua, L.; Boussalah, M.; Nouri, M. T.; Barthelmes, D.; Borruat, F. X.; Munier, F. L., Novel TULP1 mutation causing leber congenital amaurosis or early onset retinal degeneration. *Invest Ophthalmol Vis Sci* **2007,** 48, (11), 5160-7.

224. Eisenberger, T.; Slim, R.; Mansour, A.; Nauck, M.; Nurnberg, G.; Nurnberg, P.; Decker, C.; Dafinger, C.; Ebermann, I.; Bergmann, C.; Bolz, H. J., Targeted next-generation sequencing identifies a homozygous nonsense mutation in ABHD12, the gene underlying PHARC, in a family clinically diagnosed with Usher syndrome type 3. *Orphanet J Rare Dis* **2012,** 7, 59.

225. Blankman, J. L.; Long, J. Z.; Trauger, S. A.; Siuzdak, G.; Cravatt, B. F., ABHD12 controls brain lysophosphatidylserine pathways that are deregulated in a murine model of the neurodegenerative disease PHARC. *Proc Natl Acad Sci U S A* **2013,** 110, (4), 1500-5.

226. Bonnet, C.; Grati, M.; Marlin, S.; Levilliers, J.; Hardelin, J. P.; Parodi, M.; Niasme-Grare, M.; Zelenika, D.; Delepine, M.; Feldmann, D.; Jonard, L.; El-Amraoui, A.; Weil, D.; Delobel, B.; Vincent, C.; Dollfus, H.; Eliot, M. M.; David, A.; Calais, C.; Vigneron, J.; Montaut-Verient, B.; Bonneau, D.; Dubin, J.; Thauvin, C.; Duvillard, A.; Francannet, C.; Mom, T.; Lacombe, D.; Duriez, F.; Drouin-Garraud, V.; Thuillier-Obstoy, M. F.; Sigaudy, S.; Frances, A. M.; Collignon, P.; Challe, G.; Couderc, R.; Lathrop, M.; Sahel, J. A.; Weissenbach, J.; Petit, C.; Denoyelle, F., Complete exon sequencing of all known Usher syndrome genes greatly improves molecular diagnosis. *Orphanet J Rare Dis* **2011,** 6, 21.

227. McMillan, D. R.; Kayes-Wandover, K. M.; Richardson, J. A.; White, P. C., Very large G protein-coupled receptor-1, the largest known cell surface protein, is highly expressed in the developing central nervous system. *J Biol Chem* **2002,** 277, (1), 785-92.

228. Yan, W.; Long, P.; Chen, T.; Liu, W.; Yao, L.; Ren, Z.; Li, X.; Wang, J.; Xue, J.; Tao, Y.; Zhang, L.; Zhang, Z., A Natural Occurring Mouse Model with Adgrv1 Mutation of Usher Syndrome 2C and Characterization of its Recombinant Inbred Strains. *Cell Physiol Biochem* **2018,** 47, (5), 1883-1897.

229. Khateb, S.; Kowalewski, B.; Bedoni, N.; Damme, M.; Pollack, N.; Saada, A.; Obolensky, A.; Ben-Yosef, T.; Gross, M.; Dierks, T.; Banin, E.; Rivolta, C.; Sharon, D., A homozygous founder missense variant in arylsulfatase G abolishes its enzymatic activity causing atypical Usher syndrome in humans. *Genet Med* **2018,** 20, (9), 1004-1012.

230. Kowalewski, B.; Lamanna, W. C.; Lawrence, R.; Damme, M.; Stroobants, S.; Padva, M.; Kalus, I.; Frese, M. A.; Lubke, T.; Lullmann-Rauch, R.; D'Hooge, R.; Esko, J. D.; Dierks, T., Arylsulfatase G inactivation causes loss of heparan sulfate 3-O-sulfatase activity and mucopolysaccharidosis in mice. *Proc Natl Acad Sci U S A* **2012,** 109, (26), 10310-5.

231. Abitbol, M.; Thibaud, J. L.; Olby, N. J.; Hitte, C.; Puech, J. P.; Maurer, M.; Pilot-Storck, F.; Hedan, B.; Dreano, S.; Brahimi, S.; Delattre, D.; Andre, C.; Gray, F.; Delisle, F.; Caillaud, C.; Bernex, F.; Panthier, J. J.; Aubin-Houzelstein, G.; Blot, S.; Tiret, L., A canine Arylsulfatase G (ARSG) mutation leading to a sulfatase deficiency is associated with neuronal ceroid lipofuscinosis. *Proc Natl Acad Sci U S A* **2010,** 107, (33), 14775-80.

232. Di Palma, F.; Holme, R. H.; Bryda, E. C.; Belyantseva, I. A.; Pellegrino, R.; Kachar, B.; Steel, K. P.; Noben-Trauth, K., Mutations in Cdh23, encoding a new type of cadherin, cause stereocilia disorganization in waltzer, the mouse model for Usher syndrome type 1D. *Nat Genet* **2001,** 27, (1), 103-7.

233. Bahloul, A.; Michel, V.; Hardelin, J. P.; Nouaille, S.; Hoos, S.; Houdusse, A.; England, P.; Petit, C., Cadherin-23, myosin VIIa and harmonin, encoded by Usher syndrome type I genes, form a ternary complex and interact with membrane phospholipids. *Hum Mol Genet* **2010,** 19, (18), 3557-65.

234. Yokoyama, J. S.; Lam, E. T.; Ruhe, A. L.; Erdman, C. A.; Robertson, K. R.; Webb, A. A.; Williams, D. C.; Chang, M. L.; Hytonen, M. K.; Lohi, H.; Hamilton, S. P.; Neff, M. W., Variation in genes related to cochlear biology is strongly associated with adult-onset deafness in border collies. *PLoS Genet* **2012,** 8, (9), e1002898.

235. Fuster-Garcia, C.; Garcia-Garcia, G.; Jaijo, T.; Fornes, N.; Ayuso, C.; Fernandez-Burriel, M.; Sanchez-De la Morena, A.; Aller, E.; Millan, J. M., High-throughput sequencing for the molecular diagnosis of Usher syndrome reveals 42 novel mutations and consolidates CEP250 as Usher-like disease causative. *Sci Rep* **2018,** 8, (1), 17113.

236. Floriot, S.; Vesque, C.; Rodriguez, S.; Bourgain-Guglielmetti, F.; Karaiskou, A.; Gautier, M.; Duchesne, A.; Barbey, S.; Fritz, S.; Vasilescu, A.; Bertaud, M.; Moudjou, M.; Halliez, S.; Cormier-Daire, V.; Hokayem, J. E.; Nigg, E. A.; Manciaux, L.; Guatteo, R.; Cesbron, N.; Toutirais, G.; Eggen, A.; Schneider-Maunoury, S.; Boichard, D.; Sobczak-Thepot, J.; Schibler, L., C-Nap1 mutation affects centriole cohesion and is associated with a Seckel-like syndrome in cattle. *Nat Commun* **2015,** 6, 6894.

237. Namburi, P.; Ratnapriya, R.; Khateb, S.; Lazar, C. H.; Kinarty, Y.; Obolensky, A.; Erdinest, I.; Marks-Ohana, D.; Pras, E.; Ben-Yosef, T.; Newman, H.; Gross, M.; Swaroop, A.; Banin, E.; Sharon, D., Bi-allelic Truncating Mutations in CEP78, Encoding Centrosomal Protein 78, Cause Cone-Rod Degeneration with Sensorineural Hearing Loss. *Am J Hum Genet* **2016,** 99, (5), 1222-1223.

238. Wang, Y.; Li, J.; Yao, X.; Li, W.; Du, H.; Tang, M.; Xiong, W.; Chai, R.; Xu, Z., Loss of CIB2 Causes Profound Hearing Loss and Abolishes Mechanoelectrical Transduction in Mice. *Front Mol Neurosci* **2017,** 10, 401.

239. Michel, V.; Booth, K. T.; Patni, P.; Cortese, M.; Azaiez, H.; Bahloul, A.; Kahrizi, K.; Labbe, M.; Emptoz, A.; Lelli, A.; Degardin, J.; Dupont, T.; Aghaie, A.; Oficjalska-Pham, D.; Picaud, S.; Najmabadi, H.; Smith, R. J.; Bowl, M. R.; Brown, S. D.; Avan, P.; Petit, C.; El-Amraoui, A., CIB2, defective in isolated deafness, is key for auditory hair cell mechanotransduction and survival. *EMBO Mol Med* **2017,** 9, (12), 1711-1731.

240. Riazuddin, S.; Belyantseva, I. A.; Giese, A. P.; Lee, K.; Indzhykulian, A. A.; Nandamuri, S. P.; Yousaf, R.; Sinha, G. P.; Lee, S.; Terrell, D.; Hegde, R. S.; Ali, R. A.; Anwar, S.; Andrade-Elizondo, P. B.; Sirmaci, A.; Parise, L. V.; Basit, S.; Wali, A.; Ayub, M.; Ansar, M.; Ahmad, W.; Khan, S. N.; Akram, J.; Tekin, M.; Riazuddin, S.; Cook, T.; Buschbeck, E. K.; Frolenkov, G. I.; Leal, S. M.; Friedman, T. B.; Ahmed, Z. M., Alterations of the CIB2 calcium- and integrin-binding protein cause Usher syndrome type 1J and nonsyndromic deafness DFNB48. *Nat Genet* **2012,** 44, (11), 1265-71.

241. Lentz, J.; Keats, B., Usher Syndrome Type II. In *GeneReviews((R))*, Adam, M. P.; Ardinger, H. H.; Pagon, R. A.; Wallace, S. E.; Bean, L. J. H.; Stephens, K.; Amemiya, A., Eds. Seattle (WA), 1993.

242. Mburu, P.; Mustapha, M.; Varela, A.; Weil, D.; El-Amraoui, A.; Holme, R. H.; Rump, A.; Hardisty, R. E.; Blanchard, S.; Coimbra, R. S.; Perfettini, I.; Parkinson, N.; Mallon, A. M.; Glenister, P.; Rogers, M. J.; Paige, A. J.; Moir, L.; Clay, J.; Rosenthal, A.; Liu, X. Z.; Blanco, G.; Steel, K. P.; Petit, C.; Brown, S. D., Defects in whirlin, a PDZ domain molecule involved in stereocilia elongation, cause deafness in the whirler mouse and families with DFNB31. *Nat Genet* **2003,** 34, (4), 421-8.

243. Ahmed, Z. M.; Jaworek, T. J.; Sarangdhar, G. N.; Zheng, L.; Gul, K.; Khan, S. N.; Friedman, T. B.; Sisk, R. A.; Bartles, J. R.; Riazuddin, S.; Riazuddin, S., Inframe deletion of human ESPN is associated with deafness, vestibulopathy and vision impairment. *J Med Genet* **2018,** 55, (7), 479-488.

244. Zheng, L.; Sekerkova, G.; Vranich, K.; Tilney, L. G.; Mugnaini, E.; Bartles, J. R., The deaf jerker mouse has a mutation in the gene encoding the espin actin-bundling proteins of hair cell stereocilia and lacks espins. *Cell* **2000,** 102, (3), 377-85.

245. Lentz, J.; Keats, B. J. B., Usher Syndrome Type I. In *GeneReviews((R))*, Adam, M. P.; Ardinger, H. H.; Pagon, R. A.; Wallace, S. E.; Bean, L. J. H.; Stephens, K.; Amemiya, A., Eds. Seattle (WA), 1993.

246. Gibson, F.; Walsh, J.; Mburu, P.; Varela, A.; Brown, K. A.; Antonio, M.; Beisel, K. W.; Steel, K. P.; Brown, S. D., A type VII myosin encoded by the mouse deafness gene shaker-1. *Nature* **1995,** 374, (6517), 62-4.

247. Ernest, S.; Rauch, G. J.; Haffter, P.; Geisler, R.; Petit, C.; Nicolson, T., Mariner is defective in myosin VIIA: a zebrafish model for human hereditary deafness. *Hum Mol Genet* **2000,** 9, (14), 2189-96.

248. Alagramam, K. N.; Murcia, C. L.; Kwon, H. Y.; Pawlowski, K. S.; Wright, C. G.; Woychik, R. P., The mouse Ames waltzer hearing-loss mutant is caused by mutation of Pcdh15, a novel protocadherin gene. *Nat Genet* **2001,** 27, (1), 99-102.

249. Naoi, K.; Kuramoto, T.; Kuwamura, Y.; Gohma, H.; Kuwamura, M.; Serikawa, T., Characterization of the Kyoto circling (KCI) rat carrying a spontaneous nonsense mutation in the protocadherin 15 (Pcdh15) gene. *Exp Anim* **2009,** 58, (1), 1-10.

250. Johnson, K. R.; Gagnon, L. H.; Webb, L. S.; Peters, L. L.; Hawes, N. L.; Chang, B.; Zheng, Q. Y., Mouse models of USH1C and DFNB18: phenotypic and molecular analyses of two new spontaneous mutations of the Ush1c gene. *Hum Mol Genet* **2003,** 12, (23), 3075-86.

251. Lentz, J. J.; Gordon, W. C.; Farris, H. E.; MacDonald, G. H.; Cunningham, D. E.; Robbins, C. A.; Tempel, B. L.; Bazan, N. G.; Rubel, E. W.; Oesterle, E. C.; Keats, B. J., Deafness and retinal degeneration in a novel USH1C knock-in mouse model. *Dev Neurobiol* **2010,** 70, (4), 253-67.

252. Kitamura, K.; Kakoi, H.; Yoshikawa, Y.; Ochikubo, F., Ultrastructural findings in the inner ear of Jackson shaker mice. *Acta Otolaryngol* **1992,** 112, (4), 622-7.
